# SARS-CoV-2 infection of airway organoids reveals conserved use of Tetraspanin-8 by Ancestral, Delta, and Omicron variants

**DOI:** 10.1016/j.stemcr.2023.01.011

**Published:** 2023-02-23

**Authors:** Lisiena Hysenaj, Samantha Little, Kayla Kulhanek, Melia Magnen, Kriti Bahl, Oghenekevwe M. Gbenedio, Morgan Prinz, Lauren Rodriguez, Christopher Andersen, Arjun Arkal Rao, Alan Shen, Jean-Christophe Lone, Leonard C. Lupin-Jimenez, Luke R. Bonser, Nina K. Serwas, Eran Mick, Mir M. Khalid, Taha Y. Taha, Renuka Kumar, Jack Z. Li, Vivianne W. Ding, Shotaro Matsumoto, Mazharul Maishan, Bharath Sreekumar, Camille Simoneau, Irina Nazarenko, Michael G. Tomlinson, Khajida Khan, Anne von Gottberg, Alex Sigal, Mark R. Looney, Gabriela K. Fragiadakis, David M. Jablons, Charles R. Langelier, Michael Matthay, Matthew Krummel, David J. Erle, Alexis J. Combes, Anita Sil, Melanie Ott, Johannes R. Kratz, Jeroen P. Roose

**Affiliations:** 1 Department of Anatomy, University of California, San Francisco, 513 Parnassus Avenue, San Francisco, CA 94143, USA; 2 Department of Microbiology and Immunology, University of California, San Francisco, San Francisco, CA 94143, USA; 3 UCSF CoLabs, University of California, San Francisco, San Francisco, CA 94143, USA; 4 ImmunoX Initiative, University of California, San Francisco, San Francisco, CA, USA; 5 Department of Medicine, University of California, San Francisco, San Francisco, CA 94143, USA; 6 Lung Biology Center, Department of Medicine, University of California, San Francisco, San Francisco, CA, USA; 7 Department of Pathology, University of California, San Francisco, San Francisco, CA 94143, USA; 8 Division of Infectious Diseases, University of California, San Francisco, San Francisco, CA, USA; 9 Division of Pulmonary and Critical Care, San Francisco, San Francisco, CA, USA; 10 Department of Surgery, Division of Cardiothoracic Surgery, University of California, San Francisco, San Francisco, CA, USA; 11 Cardiovascular Research Institute, Departments of Medicine and Anesthesia, University of California, San Francisco, San Francisco, CA 94143, USA; 12 Gladstone Institute of Virology, Department of Medicine, University of California, San Francisco, San Francisco, CA, USA; 13 Department of Medicine, Division of Rheumatology, University of California, San Francisco, San Francisco, CA 94143, USA; 14 Quantitative Biosciences Institute COVID-19 Research Group, University of California, San Francisco, San Francisco, CA, USA; 15 Chan Zuckerberg Biohub, San Francisco, CA 94158, USA; 16 School of Life Science, University of Essex, C04 3SQ Colchester, UK; 17 School of Biosciences, University of Birmingham, Birmingham, UK; 18 Centre of Membrane Proteins and Receptors, Universities of Birmingham and Nottingham, Midlands, UK; 19 Institute for Infection Prevention and Hospital Epidemiology, University of Freiburg, Freiburg, Germany; 20 Faculty of Medicine, University of Freiburg, 79106 Freiburg, Germany; 21 German Cancer Consortium, Partner Site Freiburg and German Cancer Research Center, Heidelberg, Germany; 22 Africa Health Research Institute, Durban, South Africa; 23 School of Laboratory Medicine and Medical Sciences, University of KwaZulu-Natal, Durban, South Africa; 24 Max Planck Institute for Infection Biology, Berlin, Germany; 25 Centre for the AIDS Program of Research, Durban, South Africa; 26 National Institute for Communicable Diseases of the National Health Laboratory Service, Johannesburg, South Africa; 27 SAMRC Antibody Immunity Research Unit, University of the Witwatersrand, Johannesburg, South Africa

**Keywords:** airway organoids, spectral flow, cell composition, H1N1, SARS-CoV-2, virus, variants, single cell RNAseq, TSPAN8, therapeutics

## Abstract

Ancestral SARS coronavirus-2 (SARS-CoV-2) and variants of concern (VOC) caused a global pandemic with a spectrum of disease severity. The mechanistic explaining variations related to airway epithelium are relatively understudied. Here, we biobanked airway organoids (AO) by preserving stem cell function. We optimized viral infection with H1N1/PR8 and comprehensively characterized epithelial responses to SARS-CoV-2 infection in phenotypically stable AO from 20 different subjects. We discovered Tetraspanin-8 (TSPAN8) as a facilitator of SARS-CoV-2 infection. TSPAN8 facilitates SARS-CoV-2 infection rates independently of ACE2-Spike interaction. In head-to-head comparisons with Ancestral SARS-CoV-2, Delta and Omicron VOC displayed lower overall infection rates of AO but triggered changes in epithelial response. All variants shared highest tropism for ciliated and goblet cells. TSPAN8-blocking antibodies diminish SARS-CoV-2 infection and may spur novel avenues for COVID-19 therapy.

## Highlights

• Airway organoids from different donors display distinct compositions of cell types
• Organoid biobank models the spectrum of the epithelium response to pathogens
• TSPAN8 is a conserved mediator of infection for SARS-CoV-2 variants

## Teaser

Roose and colleagues generated a biobank of 20 airway organoids for modeling variations of airway epithelium response to SARS-CoV-2. They discovered Tetraspanin-8 (TSPAN8) as a facilitator of SARS-CoV-2 infection independently of ACE2-Spike interaction. Pre-treatment of airway organoids with a blocking TSPAN8 antibody decreased SARS-CoV-2 infection levels in airway organoids, suggesting TSPAN8 as a therapeutic target for COVID-19.

## Introduction

Severe Acute Respiratory Syndrome Coronavirus-2 (SARS-CoV-2) has caused a global pandemic of coronavirus disease (COVID-19) with more than half a billion cases worldwide (https://coronavirus.jhu.edu). While most SARS-CoV-2-infected individuals develop asymptomatic to mild disease, some develop a severe disease characterized by immune cell dysfunction (Bastard et al., 2021). Elegant work carefully mapped characteristics of SARS-CoV-2 responses in blood- and airway-immune cells. Vaccination programs have resulted in reduced cases of COVID-19 death (Pastorino et al., 2022); however, SARS-CoV-2 variants of concern (VOC) have emerged, such as Alpha (B.1.1.7), Beta (B.1.351), Delta (B.1.617.2), and Omicron (B.1.1.529) (Cobey et al., 2021; Harvey et al., 2021). Vaccinated individuals appear to retain partial T cell responses to VOC; however, Delta and Omicron escape existing neutralizing antibodies (Iketani et al., 2022; Planas et al., 2021) and caused surges in SARS-CoV-2 VOC infections (Simon-Loriere and Schwartz, 2022). The spike (Spike) glycoprotein on SARS-CoV-2 binds to human ACE2 (Yan et al., 2020), mediating membrane fusion and viral entry. Spike cleavage by host cell-type II *trans*-membrane serine proteases (TMPRSS2) results in Spike protein activation and viral entry (Hoffmann et al., 2020a). As such, ACE2 and TMPRSS2 are critical for SARS-CoV-2 entry into the cell (Wang et al., 2021); however, SARS-CoV-2 infected patients display neutralizing antibodies that bind to SARS-CoV-2 but not to Spike’s ACE2-binding domain (Brouwer et al., 2020). These findings indicate that there are likely molecular interactions, in addition to the Spike/ACE2 pair, in the extracellular environment that impact SARS-CoV-2 biology in the airway epithelium.

The lung airway epithelium defends against pollutants, allergens, and pathogens and is composed of a variety of cell types. SARS-CoV-2 reportedly infects mostly ciliated cells, goblet cells, and alveolar type 2 cells, but also basal stem cells (Chua et al., 2020; Fiege et al., 2021; Han et al., 2021; Lamers et al., 2020; Mason, 2020; Ravindra et al., 2020; Robinot et al., 2021; Salahudeen et al., 2020; Shafiee et al., 2021; Youk et al., 2020). SARS-CoV-2 elicits variation in disease spectrum of COVID-19, but the underpinnings of variation related to airway epithelium are largely unknown. Many questions remain regarding lung epithelial responses to SARS-CoV-2 infection in different people, the molecules and mechanisms that enable infection, and whether these mechanisms are conserved or distinct for different SARS-CoV-2 VOC.

Here we generated and characterized a biobank of 20 stable, but unique airway organoids (AOs) derived from adult stem cells of different individuals. We used this biobank to first optimize viral infection of AO with H1N1/PR8 influenza, and next performed a comprehensive analysis of SARS-CoV-2 infection with repeat infections. Spectral flow analysis of infected AO was used to assess cellular and functional responses of the epithelial cell compartment. Single-cell RNA sequencing (scRNA-seq) and Spectral flow enabled the discovery of Tetraspanin-8 (TSPAN8) as a conserved mediator of SARS-CoV-2 Ancestral (WA-1)-, Delta-, and Omicron-variant infection. Reductionist HEK293T cell-pseudo-virus approaches showed that TSPAN8 facilitates viral entry independently of the Spike-ACE2 interaction. We show that TSPAN8 is not an alternative entry receptor. Blocking TSPAN8 in airway epithelial organoids prior to infection is associated with a decrease in the viral load of AOs. Based on our TSPAN8 work in the context of cancer (Nazarenko et al., 2010; Voglstaetter et al., 2019), we propose that TSPAN8 as a potential therapeutic target for controlling the severity of COVID-19 disease.

## Results

### Generation of a comprehensive and stable 3D airway organoid biobank

**Figure 1:**
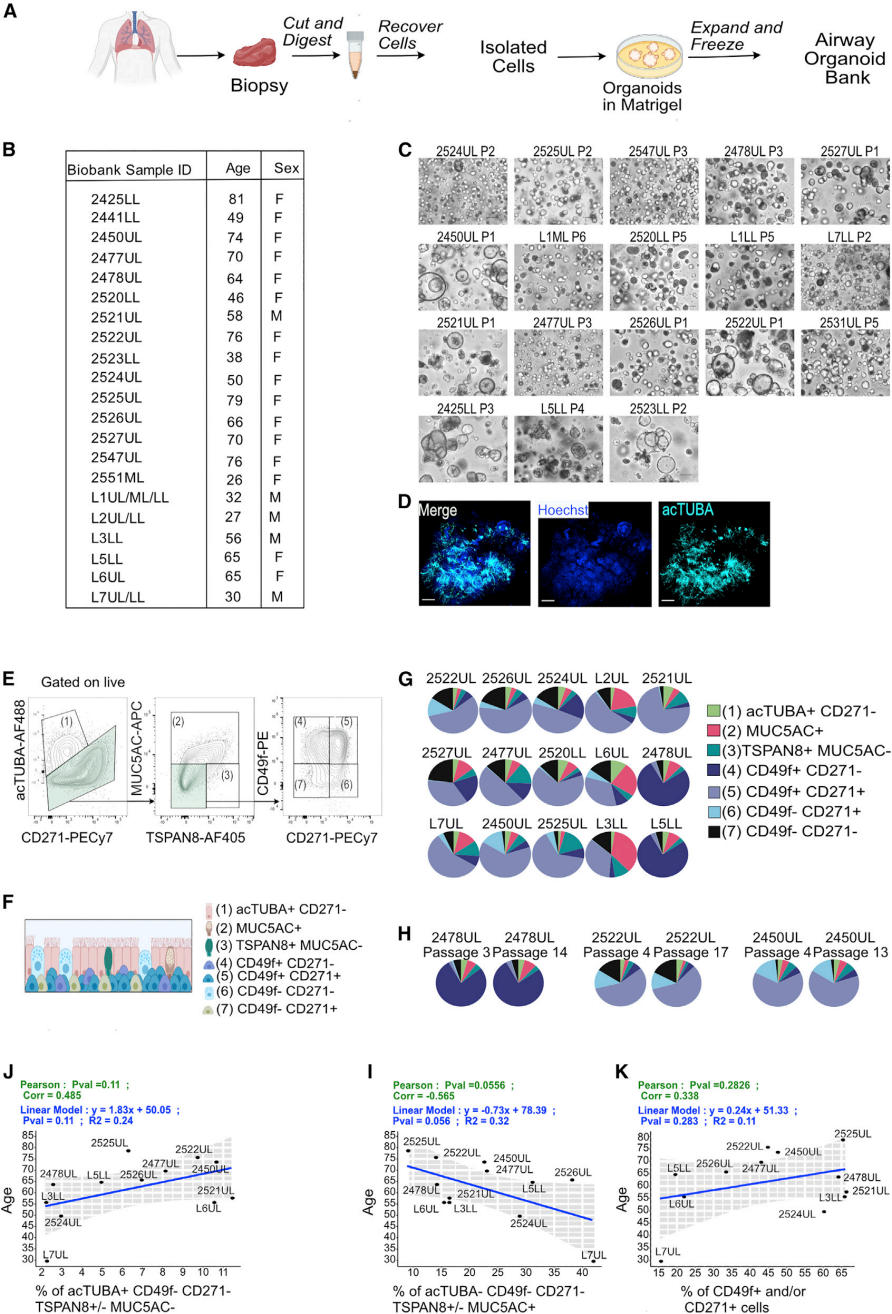
Donor-derived airway organoids are stable and distinctive (A) Workflow of airway organoid generation. (B) Table showing the age and sex of lung sample donors. (C) Brightfield images of AOs derived from different donors. Scale bars, 200 mm. (D) Confocal images (z stack) of whole-mounted organoids. Scale bars, 50 mm. Z stacks are combined into a z stack projection throughout the entire organoid and images are deconvolved to improve noise reduction and enhance contrast. (E) Spectral flow gating strategy for (1) acTUBA+ as ciliated cells, (2) MUC5AC+ acTUBA− as goblet-like cells, (3) TSPAN8+ MUC5AC− as pre-goblet cells, (4) CD49f+ CD271−, (5) CD49f+ CD271+, (6) CD49f− CD271+ as basal cells, and (7) CD49f− CD271− as undefined cells. (F) Scheme of cell types observed in the 3D AOs. (G and H) Pie charts representing AO cell-type composition from indicated donors and indicated passages (G) and from different passages of the same donor (H). (I–K) Pearson correlation showing the relationship between age of the donor and (I) % of ciliated-like cells (acTUBA+), (J) % of MUCA5AC+ cells, (K) % of CD271+ or CD49f+ cells, in the AOs. Values for Pearson correlation and p values are depicted. Functions of the positive or negative correlations are depicted by the Linear Model with R2 as value to indicate how well the linear model function agrees with the individual data points. If R2 = 0 then 0% of the data points follow the linear model, if R2 = 0.5 then 50% of the data points follow the linear model, and if R2 = 1 then 100% of the data points follow the linear model. For (G) and (K), data (pie chart fraction or dots) represent the mean value of three independent experiments with triplicates for each donor-derived organoid (DDO).

To perform a comprehensive analysis of SARS-CoV-2 infection of complex airway epithelial cell subsets in different individuals, we first generated an AO biobank from biopsies (Figure 1A and Table S1). 3D AOs from 21 subjects in the range of 26–81 years old were expanded through passaging and were cryopreserved (Figures 1B and S1C). Differential interference contrast images revealed growth of AO in Matrigel (Figure 1C) and imaging analysis of AO for acetylated Tubulin (acTUBA) confirmed the presence of ciliated cells (Figure 1D). To assess the cell-type composition and stability of AO in this panel, we performed Spectral flow cytometry analyses (termed “Spectral flow” here) on 14 reported airway epithelial markers (Bonser et al., 2021). Spectral flow enabled cell subset identification (Figures 1E, 1F, S1F, and S1G). Spectral flow revealed composite makeup with seven discrete cell populations in 15 AOs analyzed (Figure 1G). We identified ciliated-*like* cells (marked by acTUBA^high^, CD271^neg^), goblet-*like* cells (acTUBA^neg^, MUC5AC^+^) (Gray et al., 2004), pre-goblet-*like* cells (acTUBA^neg^, MUC5AC^+/−^,TSPAN8^+^), three populations of cells expressing basal cell markers CD49f^+^CD271^+^, CD49f^neg^CD271^+^, CD49f^+^CD271^+^, and a population of CD49f^neg^CD271^neg^acTUBA^neg^MUC5AC^neg^TSPAN8^neg^ cells. AOs derived from different donors displayed distinct cell-type compositions even though cultured in identical growth factors and environmental conditions (Figure 1G). Furthermore, different passages from the same donor-derived organoid (DDO) retain their patient-specific composition and are stable in composition (Figures 1H and S1A). Likewise, organoids generated from the upper and lower lobes of the lung of the same patient were very similar in makeup (Figure S1B). We generated a biobank of 20 stable, cryopreserved AOs (Figure S1C). Spectral flow for intra-cellular TMPRSS2 (Figure S1D) and extracellular ACE2 (Figure S1E) revealed the fraction of cells expressing these proteins that play critical roles in SARS-CoV-2 entry. Pearson correlations between age and goblet-*like-*, ciliated-*like*-, and basal cells in the organoids were not significant (Figures 1I–1K). So, we generated a stable and expandable biobank of 3D AO and we capitalized on it to understand SARS-CoV-2 infection in airway epithelium of different individuals.

### Benchmarking of airway organoid viral infections with H1N1 influenza

**Figure 2:**
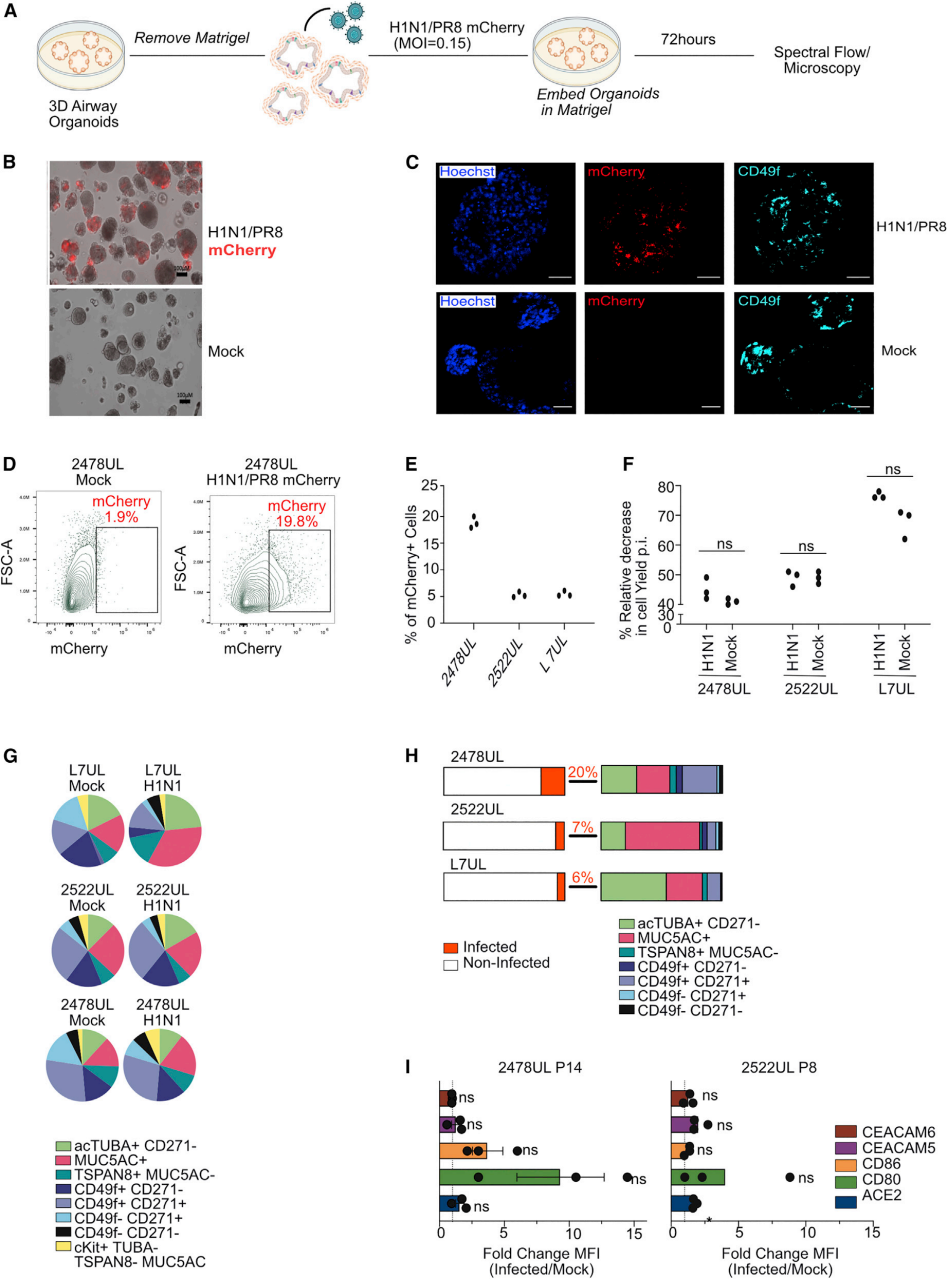
H1N1 viral infection of airway organoids (A) Experimental scheme of airway organoid infection with H1N1/PR8 mCherry virus (MOI = 0.15, analyses at 72 h p.i.). (B) Fluorescent microscopy images of H1N1-infected organoids. (C) Confocal images (z stack) of whole-mounted AOs, Hoechst (blue): nucleus, mCherry (red): H1N1/PR8+ cells, CD49f (cyan): CD49f+ cells. Scale bar, 50 μm. (D) Spectral flow layouts of mCherry+ cells. (E) Quantification of mCherry H1N1/PR8+ in 2,478, 2,522, and L7 organoids. (F) Quantification of live cell number p.i. compared with prior to infection. (G) Pie charts of cell populations distribution in AO from different donors p.i. (H) % of H1N1/PR8 mCherry+ cells (left) and bar charts of the distribution of mCherry-infected cell types (right). (I) Fold change in median fluorescence intensity (MFI) of CEACAM6, CEACAM5, CD80, CD86. (G) and (H) pie chart fractions show the mean value of three independent experiment with triplicates. For (E) and (F), dots represent the mean values of each independent experiment with triplicates per DDO. For (F) and (H), error bars are SEM. Paired t test, ^∗^p < 0.05; ns, non-significant was performed.

To benchmark reproducible viral infection of AOs, we first used H1N1/PR8 virus encoding mCherry (Figures 2A and 2B). Live imaging of whole-mount organoids through confocal microscopy for mCherry and cellular markers confirmed mCherry-positive cells throughout the 3D AO (Figures 2B and 2C). H1N1 infection levels were distinct for 2478UL, 2522UL, and L7UL organoids, but triplicate infections yielded similar infection rates for each individual organoid (Figures 2D and 2E). Live cell numbers in H1N1-infected AOs were similar compared with AOs going through the same procedures with Mock infection (Figure 2F). We used Spectral flow on mCherry and other markers to establish H1N1 tropism in distinct airway epithelial cell subsets, adding cKit as a 15th marker, as this receptor has been suggested to mark airway regeneration upon injury (Fang et al., 2012; Lopez-Giraldo et al., 2018). The Spectral flow strategies (Figure 2D) showed that H1N1/PR8 mCherry virus predominantly infected acTUBA^high^/CD271^−neg^ ciliated cells and acTUBA^neg^/MUC5AC^+^ goblet cells (Figure 2H).

Since viral infections trigger interferon-induced gene expression in epithelial cells to orchestrate immune responses (Schleimer et al., 2007), we also stained for co-stimulatory molecules CD80 and CD86 (Kaneko et al., 2000), or immune-activating molecules CEACAM5 and CEACAM6 (Lambrecht and Hammad, 2010). ACE2 is as an interferon-upregulated gene (Ziegler et al., 2020). Upregulation of these cell surface molecules was not significant, 72 h post H1N1/PR8 infection (Figure 2I).

### Application of airway organoid viral infection protocols for SARS-CoV-2

**Figure 3:**
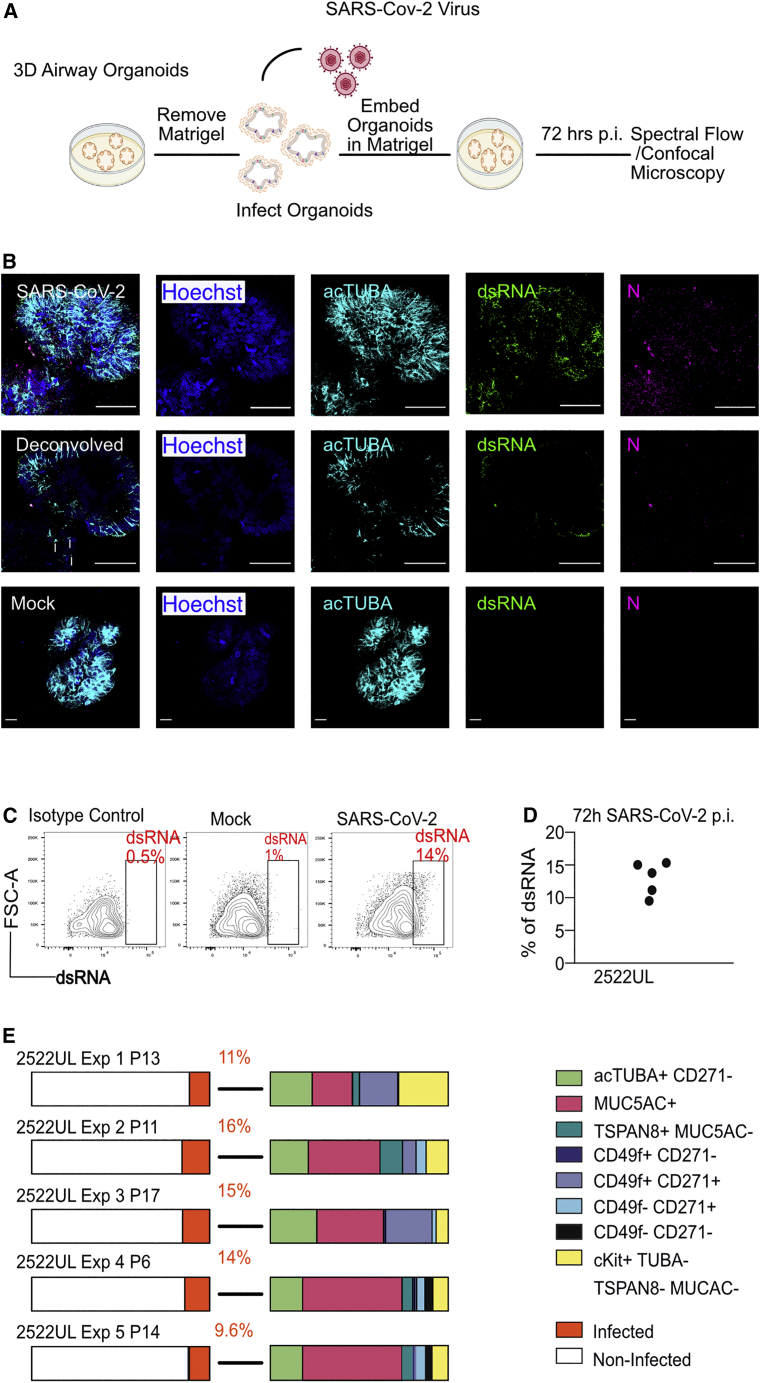
Reproducibility of SARS-CoV-2 infection in airway organoids (A) Experimental scheme of SARS-CoV-2 infection (MOI = 0.3). (B) Representative, confocal images (z stack) of SARS-CoV-2-infected (top and middle panels) and Mock-infected (bottom panel) whole-mounted organoids. Scale bars, 50 μm. For the top and bottom, all z stacks were combined into one z stack projection throughout the entire organoid image. Middle: 3 z stacks with in-focus acTUBA staining were combined into a z-projection and deconvolved to increase contrast. (C) Gating layouts of Spectral flow for dsRNA (cells with replicating SARS-CoV-2). (D) % of dsRNA+ in replicate experiments of the same DDO 72 h p.i. Dots show the mean value of each experiment with three replicates. (E) % of dsRNA + cells (left) and the bar charts (right) showing the fraction of cell types infected by SARS-CoV-2-WA-1 (dsRNA+). Bar charts show the mean value of three independent experiments with three replicates each.

We next applied our viral infection protocols with AOs to SARS-CoV-2 WA-1 at 72 h postinfection (p.i.) (Figure 3A). Confocal microscopy analyses of whole-mount organoids revealed the presence of double-stranded RNA (dsRNA) and viral nucleocapsid protein (N) in infected organoids (Figure 3B). It should be noted that removal of organoids from Matrigel induces a reverse of the organoid polarization into an apical-out model (Co et al., 2019), which can be appreciated through the acTUBA staining in Figure 3B.

Spectral flow analysis on five independent SARS-CoV-2 infections of 2522UL show that infections are consistent (Figures 3C and 3D) and an increase in cKit + cells p.i. (Figure S2A). Gating on dsRNA+ cells showed that ciliated (acTUBA+) and mucus-producing (MUC5A+) cells are predominant cell types carrying replicating SARS-CoV-2 (Figure 3E). The replicate experiments were also analyzed for expression of CEACAM6, CEACAM5, CD80, CD86, and ACE2 at 72h p.i. SARS-CoV-2 WA-1-infected cells were positive for ACE2 and a high fraction of infected cells expressed CD80, CD86, and CEACAM6 (Figure S2B).

### Identifying the host cell susceptibility factors to SARS-CoV-2 infection variation with an AO biobank

**Figure 4:**
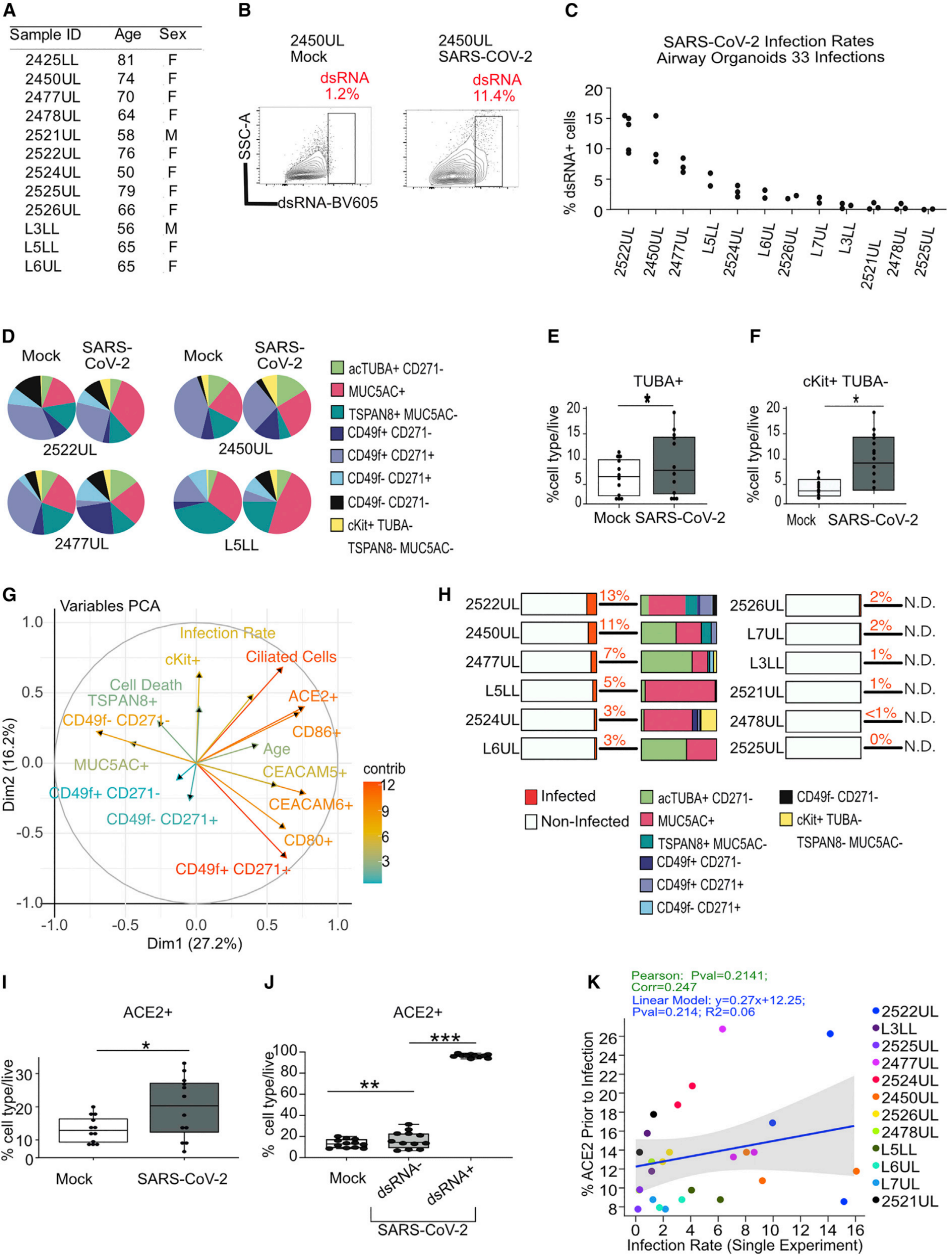
Susceptibility of airway organoids to SARS-CoV-2 infection is not predicted by ACE2 (A) Table showing donor ID, sex, and age of the lung sample donors. (B) Gating layout of Spectral flow for dsRNA (MOI = 0.3; analyses at 72 h p.i.). (C) Quantification of the fraction of dsRNA+ cells in SARS-CoV-2-infected AOs. (D) Pie charts representing the distribution of cell populations in AO from different donors p.i. Each fraction of the pie charts shows the mean value of three independent experiments with triplicates. (E and F) % of acTUBA+ cells (E) % of acTUBA-cKit+ cells (F) in all organoids combined. Each dot shows the mean value of three independent experiments for each DDO. (G) PCA of different variables impacting infection with SARS-CoV-2-WA-1. Each arrow corresponds to one biological descriptor; the longer the arrow, the better the representation (the color displays the cos2). Orange to red parameters (warm colors) contribute to differences between Mock and infected organoids. Parameters that correlate with each other are presented by arrows going in the same direction. Three independent experiments with at least three replicates per experiment were performed per DDO. (H) Stack bar charts representing the mean value from three experiments of dsRNA+ cells (in red) in infected organoids (left) and the fraction of cell types infected by SARS-CoV-2 WA-1 for each DDO. (I) % of ACE2+ cells in Mock condition (non-exposed to the virus), and in SARS-CoV-2 condition. For SARS-CoV-2 condition, the % of ACE2+ cells is shown in the fraction of dsRNA^neg^ (exposed non-infected cells) and in the fraction dsRNA^pos^ (infected cells). In (I) and (J), dots show the mean of the cell population for three experiments for each DDO. (K) Linear regression modeling the relationship between infection rate and % ACE2+ cells prior to infection. Each point represents the % cell type for the distinct donor. The mean values of each independent infection experiment are depicted and plotted. For this figure, analyses are done 72 h SARS-CoV-2 WA-1 infection at MOI = 0.3. For (E), (F), and (I) Wilcoxon signed-rank test, and for (J) Friedman test were performed, ^∗∗^p < 0.01; ^∗∗∗^p < 0.001, ns, non-significant.

To uncover the rules of infection with SARS-CoV-2, we selected a panel of 12 AOs (Figure 4A) that captured the diversity in cell composition, age, and sex. Spectral flow of dsRNA staining revealed variation in the percentage of replicating SARS-CoV-2+ cells in the 12 different organoids in 33 separate infections (Figures 4B and 4C). The organoid 2525UL expresses low TMPRSS2 (Figure S1D), providing an explanation for the low infection rate in 2525UL (Figure 4C). We investigated the rules of the remarkable variation in infection rates in the other 11 organoids.

We performed Spectral flow upon infection (Figures 4D and S2C). SARS-CoV-2 infection induces an increased proportion of cells expressing acTUBA (Figure 4E) or cKit (Figure 4F), but no alterations in the fraction of MUC5AC+, CD49^+^, or CD271+, or CD49f^neg^CD271^neg^ cells (Figures S2D–S2F). The percentage of cell death was similar between SARS-CoV-2 infected and Mock (Figure S2G), implying that cell composition alterations following SARS-CoV-2 WA-1 infection were not caused by the death of specific cell populations in 3D AO.

To investigate parameters that may correlate with efficient infection, we performed principal-component analysis (PCA) for Mock and SARS-CoV-2-infected organoids on 21 variables (cell types, cell death, infection rate, age, sex of the donors) (Figure S3A). PCA (length of lines in Figure S3A) showed that 2450UL and 2522UL reveal alterations to SARS-CoV-2 infection. Depicting PCA of many factors in a circle of correlation, we tested if there are correlations between infection rate, ciliated cells, ACE2 positivity, CD86 positivity, and possibly age (Figure 4G; warm colors and length of arrows that point in the same direction). No single factor on its own, such as age or ACE2-positivity prior to infection, significantly correlated with SARS-CoV-2 infection rate (Figures S3B–S3G). Gating on dsRNA+ cells, we observed the strongest tropism of SARS-CoV-2 for acTUBA+ and MUC5AC+ cells (Figure 4H). The fraction of ACE2+ cells was increased upon SARS-CoV-2 WA-1 infection (Figure 4I). We also observed significant increases in proportions of CD86^+^ cells (Figure S3H) upon SARS-CoV-2 infection, but no increases for CD80-, CEACAM5-, and CEACAM6-expressing cells (Figures S3I–S3L). These data argue against a general, organoid-wide induction of an interferon response program. The Spectral flow of dsRNA+ cells (replicating virus), allowed comparisons between SARS-CoV-2 exposed/infected versus exposed/uninfected and interrogation of functional molecules. Nearly 100% of dsRNA-positive cells were ACE2-positive (Figure 4J), confirming ACE2’s critical role for SARS-CoV-2 entry into the cell (Wang et al., 2021). Surprisingly, ACE2+ cell proportions in organoids *prior* to infection did not correlate with eventual SARS-CoV-2 infection rates (Figures 4K and S3G). SARS-CoV-2-infected patients display neutralizing antibodies that are not to Spike’s ACE2-binding domain (Brouwer et al., 2020; Chi et al., 2020), and these clinical findings together with the unexplained infection variation in 12 different organoids (Figure 4C) motivated us to search for novel host proteins in airway epithelial cells co-opted by SARS-CoV-2.

### TSPAN8 as novel mediator of SARS-CoV-2 infection

**Figure 5:**
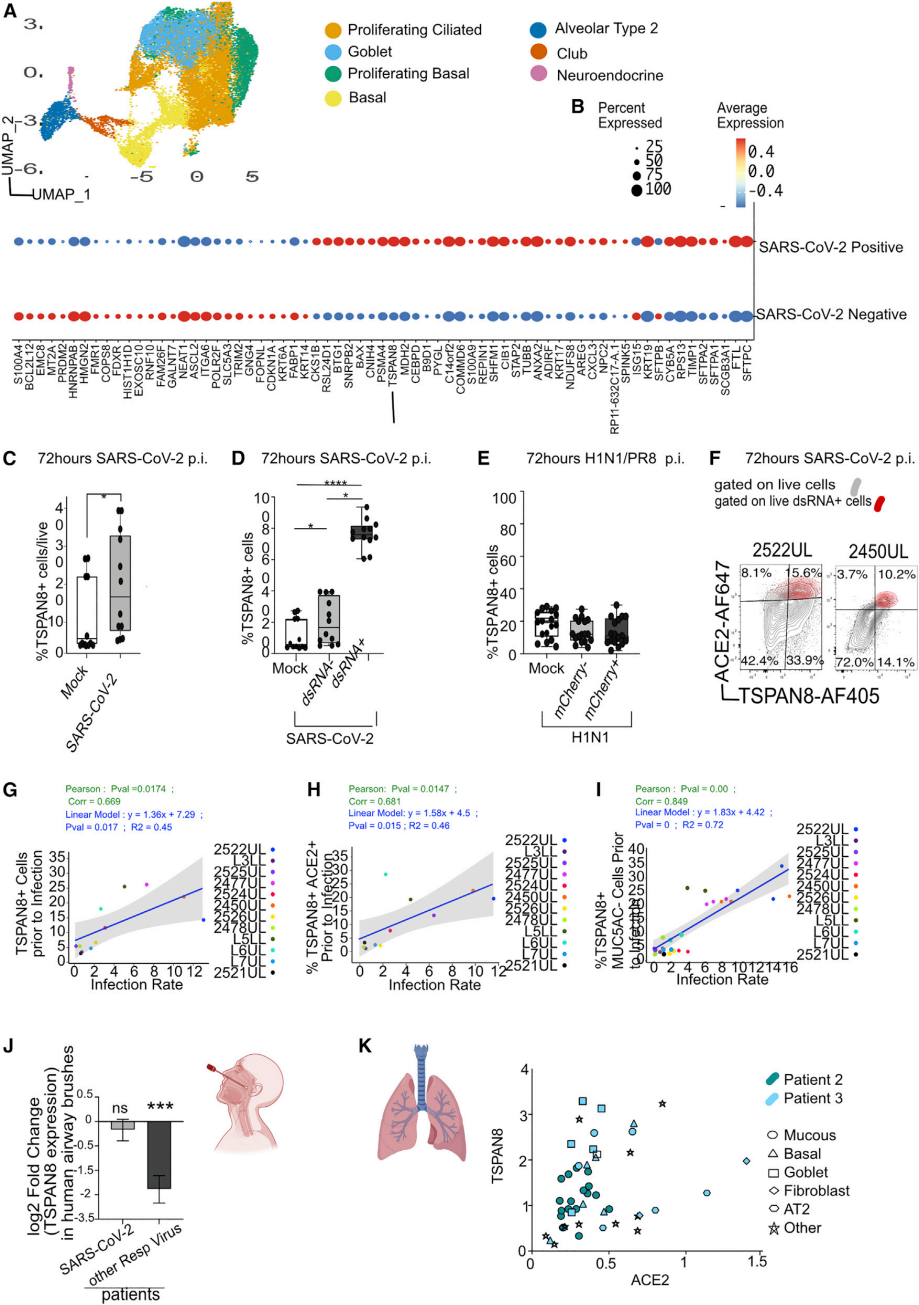
Discovery of TSPAN8 in SARS-CoV-2-infected airway organoids (A) UMAP reduction on the merged cell data with overlaid clusters and identified cell types. (B) Top differentially upregulated genes, including *TSPAN8* (arrow), within a representative random subsample of SARS-CoV-2-positive (top) versus SARS-CoV-2 negative (bottom) cells. Circle sizes indicate the % of cells within the total cell population that the specific gene is expressed. (C) Box and whisker plots representing the % of TSPAN8-positive cells in AOs for Mock and SARS-CoV-2 condition at 72 h p.i. (MOI = 0.3). (D) Box and whisker plots representing the % of TSPAN8+ cells in AOs for Mock and SARS-CoV-2-infected conditions. For the SARS-CoV-2 condition, the % of TSPAN8+ cells is quantified in non-infected (dsRNA−) and infected (dsRNA+) cells. (E) Same as (D) but for mCherry H1N1/PR8-infected organoids. (C–F)Dots show the mean value of three independent experiments with three replicates each for each DDO. Wilcoxon signed-rank test, ^∗^Pv < 0.05 was performed. (F) Representative Spectral flow plots of overlay between dsRNA cells (in gray) and SARS-CoV-2-infected, dsRNA+ cells (in red) in AOs for 2,522 organoid. The x axis represents TSPAN8 expression intensity and the y axis represents ACE2 expression intensity. (G–I) ScatterPlot showing the relationship among (G) % of TSPAN8+ cells, (H) TSPAN8+ ACE2+, (I) TSPAN8+ MUC5AC− prior to infection and the infection rate. Pearson correlation statistical significance stated on graph (Pval.). (J) Differential expression of TSPAN8 in nasal swabs of adult patients with acute respiratory illness (ARI) due to COVID-19 (n = 93) or other viral infection (n = 41), in comparison to patients with ARI due to non-viral etiology (n = 100). Pearson’s chi-squared test, ^∗∗∗^Pv < 0.001, ns, non-significant. (K) Single-cell sequencing was performed to analyze AOs from two different donors from patients undergoing lobectomy for focal airway tumors (Travaglini et al., 2020). The graph shows cells expressing TSPAN8 and ACE2 from different patients. Of the 60,993 cells derived from airway tissue of three patient donors in this dataset, 48 cells were found with at least one unique molecular identifier (UMI) for both genes. Only one cell derived from patient 1, which had fewer cells sequenced overall and so we excluded it. The expression values represent ln(UMI-per-10K + 1) in each of the 47 cells from patients 2 and 3. Cell-type designations were determined by Travaglini et al. (2020).

We infected four organoids (2522UL, 2450UL, L7UL, and 2524UL) with SARS-CoV-2 WA-1 and performed scRNA-seq. Unsupervised clustering analysis based on most variable gene expression across all cells (Becht et al., 2018), regardless of infection status, identified seven unique cell subsets represented in a UMAP plot (Figure 5A). Based on the top five most differentially expressed genes by cluster (Figure S4A) and published work (Travaglini et al., 2020; Vieira Braga et al., 2019), we assigned relative identities to these seven populations. These scRNA-seq analyses corroborated our Spectral flow results demonstrating that our 3D AOs have a complex makeup of cell types. It should be noted that specific single-cell characterization of different AOs was not our objective in this study. BSL-3 restrictions prevented us from running the control that we perform in other studies (Gonzalez et al., 2022) and for unknown reasons read counts were relatively low for organoids L7 and 2524. We, therefore, treated the four organoids as one collective dataset for discovery of novel mediators with scRNA-seq resolution. Interrogating cells expressing viral SARS-CoV-2 transcript, we identified specific transcriptomic signatures in infected cells (Figure S4B and Table S2) and genes differentially expressed in single cells, comparing positive for viral read identities with false identities (Figure 5B and Table S3). Neutralizing antibodies in COVID-19 patients suggest the existence of multiple targets (Brouwer et al., 2020; Chi et al., 2020) and we mapped several of these suggested genes back onto our AO UMAP (Figure S4C). For the remainder of this study, we focused on TSPAN8 (Figure 5B, Figure S4C, and Table S3), since members of the TSPAN family have been reported to promote cell entry of different viruses and depletion of TSPAN8, CD9, in mice reduced MERS-CoV lung titers by ∼90% (Earnest et al., 2017). In addition, the presence of TSPAN8 in infected cells was documented in supplemental data of a single SARS-CoV-2-infected lung organoid (Lamers et al., 2020), but the role of TSPAN8 in SARS-CoV-2 infection has not been investigated.

scRNA-seq of SARS-CoV-2-infected organoids showed that *TSPAN8* mRNA reads were present in 64% of single cells positive for SARS-CoV-2 reads (size of the circle in Figure 5B). Fortuitously, the anti-TSPAN8 antibody was included in our Spectral flow to distinguish goblet cells (MUC5AC^+^TSPAN8^−^) from pre-goblet cells (MUC5AC^−^TSPAN8^+^) (Figure S2), allowing investigation of the role of TSPAN8 surface protein in SARS-CoV-2 infection. The number of TSPAN8-positive cells increased upon SARS-CoV-2 infection (Figure 5C) but decreased upon H1N1/PR8 infection (Figure S5A). As we observed for ACE2 (Figure 4J), most SARS-CoV-2-infected cells expressed TSPAN8 (Figure 5D), whereas H1N1 infected cells do not (Figure 5E). Furthermore, most SARS-CoV-2 infected cells in our AO co-expressed ACE2 and TSPAN8 on the cell surface (Figures 5F and S5B). Different from ACE2 (Figure 4K), the proportion of TSPAN8+ cells *prior* to infection correlated with levels of eventual infection (Figures 5G and S5C), suggesting TSPAN8 somehow facilitates SARS-CoV-2 infection. These correlations also held true for TSPAN8^+^ACE2^+^ and TSPAN8^+^MUC5AC^−^ cells prior to infection (Figures 5H and 5I).

In infected patients, *TSPAN8* expression decreases in airway brushes of acute illness in patients caused by non-SARS-CoV-2 respiratory viruses, while airway brushes from COVID-19 patients revealed preservation of *TSPAN8* levels (Figure 5J), despite depletion in goblet cells (Mick et al., 2020). In addition, in the lungs of two COVID-19 patients, we could detect cells that express ACE2 and TSPAN8 concomitantly (Figure 5K). Collectively, the clinical and 3D airway organoid data suggest that TSPAN8 facilitates SARS-CoV-2 infection.

### A reductionist 293T platform to investigate TSPAN8

**Figure 6:**
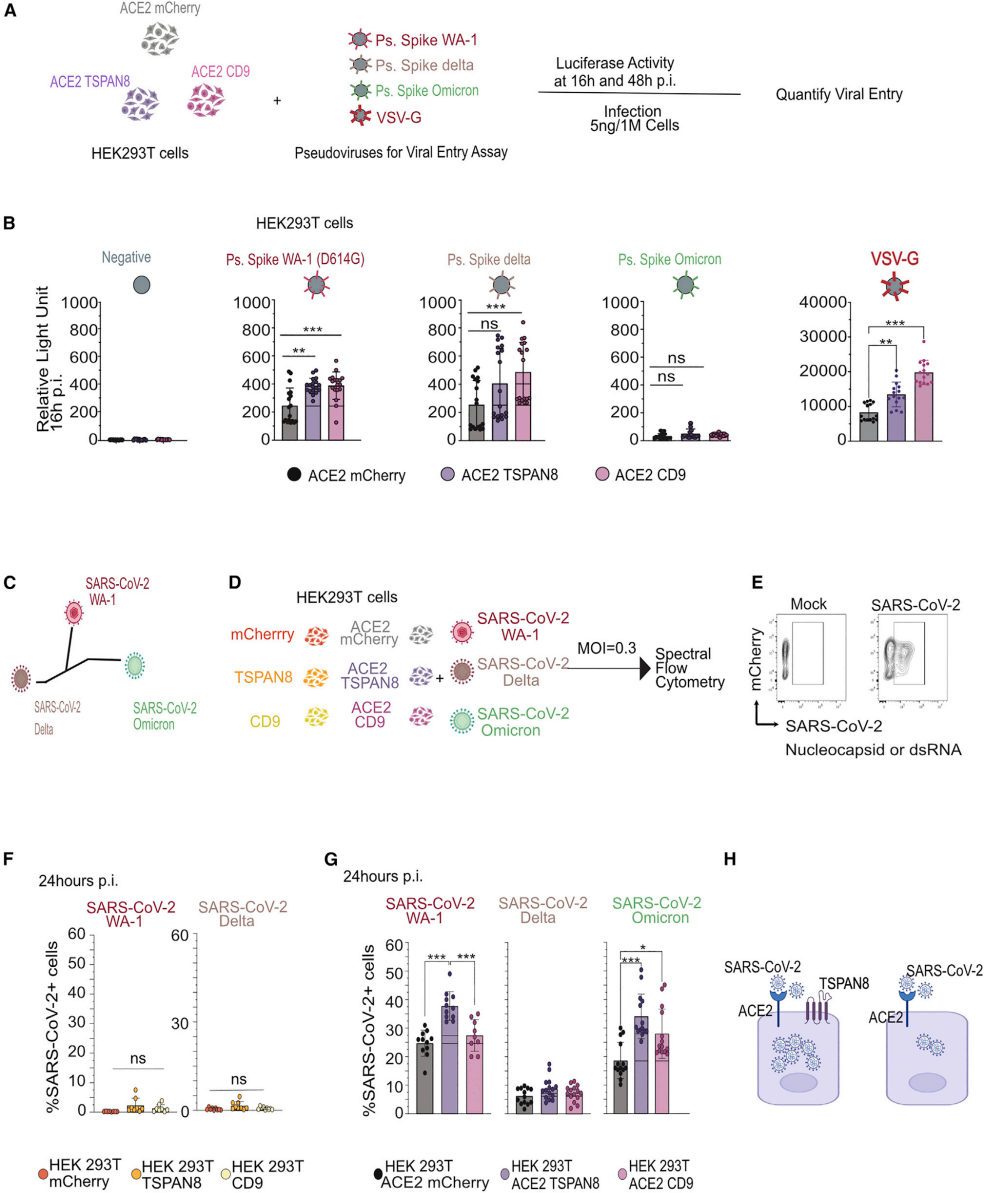
TSPAN8 serves as a facilitator for SARS-CoV-2 WA-1, Delta, and Omicron variants (A) Scheme of workflow for infection HEK293T cell lines with the Ps-virus expressing Spike protein of SARS-CoV-2 WA-1 (USA-WA1/2020) or SARS-CoV-2 Delta (SARS-CoV-2 B1.1617.2) or SARS-CoV-2 (Omicron BA1.1.529) or VSV-G virus (as control). Viral entry is quantified 16 h or 48 h postinfection by measuring luciferase activity. (B) Luminescence (relative light unit) measured as a function of Ps-virus entry for the backbone Ps-virus, WA-1, Delta, Omicron Ps-virus and VSV-G at 16 h p.i. (C) Scheme of SARS-CoV-2 phylogenetic tree representing SARS-CoV-2 WA-1, Delta, and Omicron. (D) Workflow of HEK293T cell lines infection with live SARS-CoV-2. (E) Flow cytometry plot of Nucleocapsid+ cells post SARS-CoV-2 infection. (F) SARS-CoV-2 Nucleocapsid+ fraction in mCherry, TSPAN8, and CD9 HEK293T cell at 24 h post SARS-CoV-2 WA-1 or Delta infection (MOI = 0.3). (G) SARS-CoV-2 Nucleocapsid+ cells fraction in ACE2 mCherry-, ACE2 TSPAN8-, and ACE2 CD9-expressing HEK293T cells at 24 h post SARS-CoV-2 WA-1, Delta, or Omicron infection (24 h p.i., MOI = 0.3). (F–H) Three independent experiments with four replicates. Nonparametric ANOVA tested corrected by Geisser Greenhouse Correction was performed to compare between different conditions. ^∗^Pv < 0.05; ^∗∗∗^Pv < 0.001. (H) Scheme showing TSPAN8 as a facilitator of infection.

We generated a panel of six HEK 293T cell lines, stably expressing ACE2, TSPAN8, or TSPAN8 CD9 as a control to enable a reductionist approach to investigate TSPAN8 (Figure 6A). HEK 293T cells do not express extracellular ACE2, TSPAN8, or CD9. The expression of TSPAN8 or CD9 did not alter ACE2 expression levels (Figures S6A and S6B) or overall subcellular localization (data not shown). We used these cell lines to assess viral entry with designed, replication-deficient, luciferase-expressing pseudo-viruses (Ps-virus) expressing Spike of SARS-CoV-2 WA-1, or Delta, or Omicron, or VSV-G (vesicular stomatitis virus-G) (Figures 6A and S6C). VSV-G does not use ACE2 for the entry and serves as control (Finkelshtein et al., 2013).

Entry of Omicron Spike Ps-virus in ACE2 HEK293T, with or without TSPAN8 or CD9, was relatively inefficient (Figures 6B and S6D), which may reflect the altered usage of proteases by Omicron (Meng et al., 2022). Entry of WA-1 (D614G) Spike-, Delta Spike-, and VSV-G- carrying Ps-virus was robust (Figures 6B and S6D). Expression of TSPAN8 or CD9 with ACE2 in HEK 293T cells resulted in roughly 2-fold increases in luciferase activity, but without specificity for Spike or VSV-G Ps-viruses (Figures 6B and S6D). So, TSPAN8 and CD9 enhance viral entry independently of the Spike/ACE2. These Ps-virus-based results are in line with previous reports that TSPANs promote the entry of multiple viruses (Earnest et al., 2015; Hantak et al., 2019).

We next analyzed live virus infection. Phylogenic and genetic analyses have shown that SARS-CoV-2 variants differ not only in their Spike protein but also in other proteins (Thorne et al., 2022) (Figures 6C and S6E). We propagated SARS-CoV-2 WA-1, Delta, and Omicron variants in Vero E6 cells (Figure S6E) with similar efficiency (Figure S6F) and infected the HEK293T cells (Figure 6D). None of the SARS-CoV-2 variants were able to infect TSPAN8- or CD9-expressing HEK293T without ACE2 (Figures 6F and S6G). So, TSPAN8 or CD9 are not alternative entry receptors. Levels of nucleocapsid+ HEK293T cells were unexpectedly modest for SARS-CoV-2 Delta but at expected levels for Omicron (Figure 6G) even though initial entry (Ps-virus) is inefficient (Figure 6B). The presence of TSPAN8 and CD9 in HEK293T cells increased Nucleocapsid+ cells for Omicron infections (Figure 6G). Only TSPAN8, but not CD9, was responsible for an increase of the Nucleocapsid+ cells for WA-1. Of note, the different SARS-CoV-2 variants did not differentially impact HEK293T viability (Figure S6H). Collectively, the HEK293T approach revealed that TSPAN8 facilitates SARS-CoV-2 infection rates independently of Spike/ACE2 interaction.

### AOs reveal conserved use of TSPAN8 by SARS-CoV-2 AW-1, Delta, and Omicron variants

**Figure 7:**
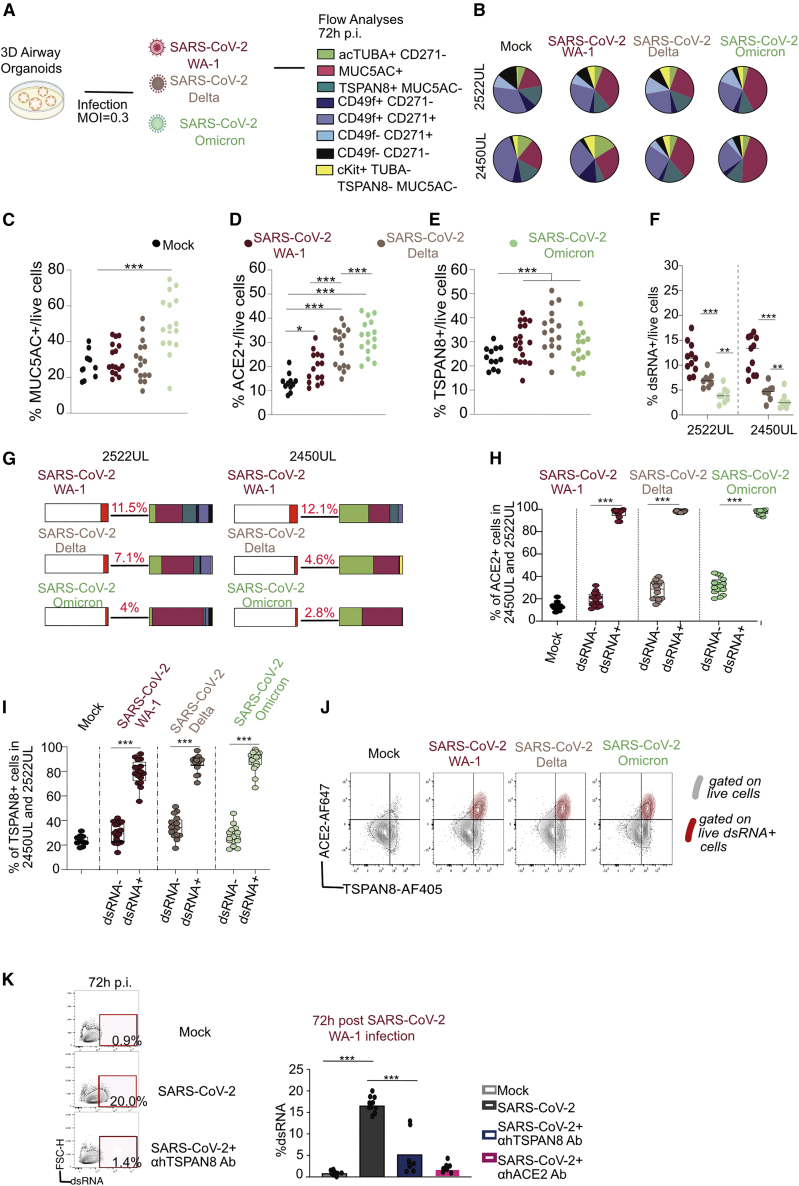
Conserved role of TSPAN8 in SARS-CoV-2 WA-1, Delta, and Omicron (A) Workflow of organoid infection by SARS-CoV-2 WA-1, Delta, and Omicron infection (MOI = 0.3, 72 h p.i.). (B) Pie charts of cell populations distribution in Mock, with SARS-CoV-2 WA-1-, -Delta-, or Omicron-infected AOs. Each fraction of the pie chart shows the mean of three independent experiments with triplicates. (C–F) % of cells fraction in live cells for Mock or SARS-CoV-2 WA-1-, Delta-, or Omicron-infected AO: MUC5AC+ (C), ACE2+ (D), TSPAN8+ (E), dsRNA+ (F). Dots show the data for each replicate. Experiments are repeated three times with at least three replicates. Nonparametric ANOVA tested corrected by Geisser Greenhouse Correction was performed to compare different conditions. ^∗∗∗^Pv < 0.001. (G) Stack bar charts representing the mean value for three independent experiments of dsRNA+ cells (in red) in SARS-CoV-2-infected organoids (left) and the fraction of cell types infected by SARS-CoV-2 WA-1, Delta, or Omicron (dsRNA+). (H) Plots representing the % of ACE2+ cells analyzed by Spectral flow in live cells for Mock, SARS-CoV-2 WA-1, Delta, or Omicron conditions. For SARS-CoV-2-infected organoids, the fraction of ACE2+ cells is shown in exposed, non-infected cells (dsRNA−) and infected cells (dsRNA+). (I) As in (H) but for TSPAN8-positive cells. (J) Representative Spectral flow plots of overlaid dsRNA− cells (in gray) and dsRNA+ cells (in red) of infected organoids with SARS-CoV-2 WA-1, Delta, or Omicron. The x axis represents TSPAN8 expression intensity and y axis represents ACE2 expression intensity. (K) Spectral flow of dsRNA+ cells in organoids pre-treated with Mock (TSPAN8 isotype control), TSPAN8, and/or ACE2 blocking antibody (50 μg/mL) at 72 h post SARS-CoV-2 WA-1 infection (MOI = 0.3).

Last, we investigated the infection characteristics of SARS-CoV-2 WA-1, Delta, and Omicron variants in the context of 3D AO with their diverse cell-type composition and subsequently related results to TSPAN8. We used organoids 2522UL and 2450UL in head-to-head comparisons, which demonstrated highest infection with the WA-1 variant at 72 h p.i. (Figure 7A). SARS-CoV-2 variants elicited distinct effects on cell composition (Figures 7B and S7A–S7E). A selective increase in MUC5AC+ cells with Omicron (Figure 7C), as well as in cKit+ cells with WA-1 infection (Figure S7C) was noted. Analysis of functional markers demonstrated stepwise increases in ACE2+ and CD86^+^ cells for the WA-1, Delta, or Omicron variants (Figures 7D and S7F). CD86 (Corbiere et al., 2011) and ACE2 (Ziegler et al., 2020) are interferon-stimulated genes implying that SARS-CoV-2 VOC trigger increasing strengths of interferon responses in airway epithelial organoids. The fraction of TSPAN8+ cells increased following all SARS-CoV-2 variant infections, but there was no difference between the VOCs (Figure 7E).

Our AOs do not contain immune cells. With the notion that our organoids are a purely epithelial cell platform, it is striking that the percentage of infected cells in AOs decreased stepwise, in head-to-head comparisons of WA-1, to Delta, to Omicron (Figure 7F). Analyzing the dsRNA-positive cells with replicating virus, SARS-CoV-2 AW-1, Delta, and Omicron all displayed clear tropism for acTUBA+ (ciliated) and MUC5AC+ (goblet) cells (Figures 7G and S7G). For all SARS-CoV-2 variants, close to 100% of infected cells (dsRNA+) displayed ACE2 expression (Figures 7H and S7H), highlighting the role of ACE2 as an entry receptor (Hoffmann et al., 2020b). Roughly 80% of all SARS-CoV-2-infected cells expressed TSPAN8 (Figures 7I and S7I), indicating that TSPAN8 is critical as well. While AO cell infection rates decreased sequentially in SARS-CoV-2 from WA-1 to Delta to Omicron variants (Figure 7I), the presence of TSPAN8 in infected cells remained conserved (Figure 7J). The conserved role of TSPAN8 for all SARS-CoV-2 VOCs imply that TSPAN8 is an attractive therapeutic target to limit COVID-19, which we explored with a TSPAN8-blocking antibody approach developed in the oncology field (Bonnet et al., 2019; Kim et al., 2015). Infection levels of 2522UL and 2450UL organoids with SARS-CoV-2 WA-1 were reduced by 60% upon TSPAN8-blocking antibody treatment (Figures 7K, S7J, and S7K), demonstrating that TSPAN8 plays a functional role in SARS-CoV-2 infection.

## Discussion

SARS-CoV-2 research has focused on the immune cells (Schultze and Aschenbrenner, 2021; Sette and Crotty, 2021), but many questions regarding airway epithelial responses to SARS-CoV-2 infection have remained unanswered. Clonal cell lines are efficient discovery tools but lack variations in genetic and proteomic traits. Lung epithelial organoids contain diverse cell types (Sachs et al., 2019). Here, we characterized AO from many subjects and capitalized on the diversity and cell-type-complexity in our large panel of AOs to (1) understand the underpinnings of epithelial cell infection by SARS-CoV-2 WA-1, Delta, and Omicron variants, and (2) discover TSPAN8 as a conserved mediator of infection with all three variants.

Our study with a comprehensive panel of AOs capitalized on the unique diversity in AOs derived from different subjects to understand airway epithelial characteristics that impact SARS-CoV-2 infection. Essential here was the Spectral flow approach that allows cell-type characterization. Our AOs from our model are embedded in Matrigel as we cannot obtain the high throughput for the study presented here with organoids in air-liquid interface (Sachs et al., 2019). SARS-CoV-2 WA-1, Delta, and Omicron variants shared highest tropism for ciliated and goblet cells. The three SARS-CoV-2 variants elicited distinct cell-type composition effects in AOs; selective increases in MUC5AC+ cells for Omicron and cKit+ cells for WA-1 were striking. MUC5AC and cKit are both suggested to play a role in airway epithelium regeneration (Letuve et al., 2019; Xian and McKeon, 2012), indicating different regeneration responses in the AOs triggered by different strains. Interferons upregulate CD80, CD86 (Kaneko et al., 2000), CEACAM5, and CEACAM6 (Lambrecht and Hammad, 2010) and ACE2 itself (Ziegler et al., 2020). ACE2, CD80, CD86, and CAECAM6 (Figures S3H–S3L and 4I) expression levels were upregulated in infected cells (dsRNA-positive), but not in all cells in the epithelial AO, arguing against an organoid-wide interferon response. With that notion, it was remarkable that WA-1, Delta, or Omicron variants yielded stepwise increases in ACE2- and CD86-positive cells, suggesting that SARS-CoV-2 VOC trigger stronger interferon responses in airway epithelial organoids than Ancestral WA-1.

Neutralizing antibodies from COVID-19 patients have multiple targets (Brouwer et al., 2020; Chi et al., 2020), suggesting that these protective antibodies may block interactions of cell surface molecules other than the Spike protein-ACE2 receptor pair. The WA-1, Delta, or Omicron variants all led to increases in TSPAN8-positive cells in AOs. In addition, more than 80% of all SARS-CoV-2-infected cells expressed TSPAN8.

TSPAN8 proteins have four *trans*-membrane domains that form lateral associations with multiple molecular partners and with each other, organizing the surface membrane proteins in a dynamic microdomain (Hantak et al., 2019; Kummer et al., 2020). TSPAN8s promote the entry of multiple viruses, including influenza A virus, human cytomegalovirus (HCMV), human papillomavirus, etc (Hantak et al., 2019). We demonstrated that neither TSPAN8, nor CD9, can function as an alternative entry receptor in HEK293T cells. TSPAN8 and CD9 increase entry of Ps-viruses in HEK293T cells, but not via the ACE2 receptor.

We have previously reported in cancer cell lines that TSPAN8 increases extracellular vesicle or exosome (EV) numbers and influences their composition (Nazarenko et al., 2010; Voglstaetter et al., 2019). EV from virus-infected cells can infect or prime to neighbor cells (Martins and Alves, 2020). Circulating EVs have been implicated in SARS-CoV-2 infection (Barberis et al., 2021). We think that TSPAN8 contributes to the spreading of infection through EVs in the case of SARS-CoV-2 infection. Future work is required to understand how TSPAN8, CD9, or other TSPAN8s are involved in SARS-CoV-2 infection. We demonstrated that the addition of a TSPAN8-blocking antibody to the AO prior to infection decreased SARS-CoV-2 infection. VOCs escape from the therapeutic antibody neutralization (Planas et al., 2022; Sigal, 2022; VanBlargan et al., 2022), it will be of value to have other avenues of interfering with SARS-CoV-2. In conclusion, our study demonstrates that donor-derived AOs can be used to model the spectrum of the response of the human airway epithelium to airway pathogens and identify novel therapeutic targets.

## Experimental procedures

### Resource availability

#### Corresponding author

jeroen.roose@ucsf.edu.

#### Materials availability

A list of materials used and detailed methods are found on the Supplementary Materials section. Materials are available upon request.

#### Data and code availability

Resources: GEO: GSE211562.

## Author contributions

L.H. and J.P.R.: conceived the study. L.H., S.L., and K.K.: Spectral flow. L.H., K.K., S.L., O.M.G.: organoid biobank. L.H., M.M., and L.R.: BSL3 work. C.A., A.A.R., and A.J.C.: scRNA-seq. J.C.L.: statistical analyses. C.A., L.C.L.: scRNA-seq analyses. L.R.B. and D.J.E.: advice on airway populations. N.K.S. and M.K.: H1N1 virus. L.H., S.L., O.M.G., and K.B.: microscopy. L.H., S.L., K.B.: HEK239T cell line creation. J.Z.L., V.D., S.M., M.M.: patient samples. G.K., D.M.J., M.M., A.J.C.: funding. M.O.: SARS-CoV-2 virus, and SARS-CoV-2 pseudo-viruses. S.L., M.P.: pseudo-virus infections. J.R.K.: surgery airway samples, clinical data discussion. L.H., S.L., J.P.R.: manuscript writing. J.P.R.: funding. G.K.F., D.M.J., M.M., M.O., M.M., A.J.C., D.E., A.N.S., and J.R.K.: edits on the manuscript.

## Supplemental information

Document S1. Figures S1–S7 and supplemental materials and methods

Table S1. Lung sample details

Table S2. Supplement scRNAseq data analysis

Table S3. Viral reads analysis scRNAseq

Document S2. Article plus supplemental information
