# Supplementary material for: SARS-CoV-2 infection of airway organoids reveals conserved use of Tetraspanin-8 by Ancestral, Delta, and Omicron variants
Source: Stem Cell Reports. 2023 Feb 23;18(3):636–53. doi: 10.1016/j.stemcr.2023.01.011 (PMC9948283; doi:10.1016/j.stemcr.2023.01.011)
Supplement: Document S1. Figures S1–S7 and supplemental materials and methods [file mmc1.pdf]

Stem Cell Reports, Volume *18*

Supplemental Information

SARS-CoV-2 infection of airway organoids reveals conserved use of Tetraspanin-8 by Ancestral, Delta, and Omicron variants

Lisiena Hysenaj, Samantha Little, Kayla Kulhanek, Melia Magnen, Kriti Bahl, Oghenekevwe M. Gbenedio, Morgan Prinz, Lauren Rodriguez, Christopher Andersen, Arjun Arkal Rao, Alan Shen, Jean-Christophe Lone, Leonard C. Lupin-Jimenez, Luke R. Bonser, Nina K. Serwas, Eran Mick, Mir M. Khalid, Taha Y. Taha, Renuka Kumar, Jack Z. Li, Vivianne W. Ding, Shotaro Matsumoto, Mazharul Maishan, Bharath Sreekumar, Camille Simoneau, Irina Nazarenko, Michael G. Tomlinson, Khajida Khan, Anne von Gottberg, Alex Sigal, Mark R. Looney, Gabriela K. Fragiadakis, David M. Jablons, Charles R. Langelier, Michael Matthay, Matthew Krummel, David J. Erle, Alexis J. Combes, Anita Sil, Melanie Ott, Johannes R. Kratz, and Jeroen P. Roose

SARS-CoV-2 infection of Airway Organoids Reveals Conserved Use of Tetraspanin-8 by Ancestral-, Delta-, and Omicron- Variants

Materials and Methods

KEY RESSOURCES TABLE

**Cell lines**	**Supplier**	**Catalogue**
HEK 293T ACE2	Integral Molecular	C-HA102
HEK 293T	ATCC	CRL-3216
Vero E6 cell lines	ATCC	61262

**Media component**	**Supplier**	**Catalogue number**
R-Spondin 1	Peprotech	120-38
FGF 7	Peprotech	100-19
FGF 10	Peprotech	100-26
Noggin	Peprotech	120-10C
A83-01	Tocris	2939
Y-27632	Abmole	Y-27632
SB202190	Sigma	S7067
B27 supplement	Gibco	17504-44
N-Acetylcysteine	Sigma	A9165-5g
Nicotinamide	Sigma	N0636
GlutaMax 100x	Invitrogen	12634-034
Hepes	Invitrogen	15630-056
Penicillin / Streptomycin	Invitrogen	15140-122
Primocin	Invivogen	Ant-pm-1
Advanced DMEM/F12	Invitrogen	12634-034
Liberase™ TL Research Grade	ThermoFischer	5401020001
EDTA	ThermoFischer	03690-100ML
Cyto-Fast™ Fix/Perm Buffer Set	ThermoFischer	426803

BSA	ThermoFischer	A7030-10G
PBS without calcium, magnesium	UCSF	
Dead Cell Removal Kit		130090101

**Antibodies**	**Supplier**	**catalog #**
TSPAN8 Alexa Fluor 405	R&D systems	FAB4734V-100UG
CD66c Briliant Violet 510	BD	742684
CD86 Briliant Violet 650	BioLegend	305427
EPCAM Briliant Violet 711	BioLegend	324239
ckit (CD117) Briliant Violet 785	BioLegend	313237
CD49f PE	BioLegend	313612
CD80 PE-Cy5	bioLegend	305209
CD271 PE-Cy7	bioLegend	345109
ACE2 Alexa Fluor	R&D systems	FAB933R-100UG
CEACAM5	R&D systems	FAB41281S-100UG
Zombie NIR	Biolegend	--
TrueStain	Innovex Biosciences Inc.	NB309-5S
TUBA	Sigma-Aldrich	sc-23950 AF488
MUC5AC Biotin	Thermo Fischer Scientific	MA5-12175
TMPRSS2	Abcam	ab242384
dsRNA	Scicons	

**Software**		
GraphPad Prism 9		
Fiji/ImageJ (V) software 2.1.0/1.53c		
FlowJO Software V10.1		
Seurat		

EXPERIMENTAL MODEL AND SUBJECT DETAILS

Patient samples

Normal adjacent lung tissue was collected from non-small cell lung cancer patients; 2425LL, 2450UL, 2477UL, 2478UL, 2520LL, 2521UL, 2522UL, 2524UL, 2525UL, 2526UL, 2527UL, 2531UL, 2547UL, 2551ML. Samples were collected under UCSF study CC#00654 (IRB 11-06107) from patients undergoing thoracic surgery.

Donor lungs rejected for transplantation were received from organ procurement organization (Donor Network West) as previously described (Ross et al., 2019); samples L1, L2, L3, L5, L6, and L7. Explicit approval for the use of donor lungs for research was sought from each donor's family by Donor Network West as part of the standard organ donation process. Local institutional review board approval is not required because research on tissues from deceased organ donors is not considered human subject research, although institutional biosafety approval was obtained from University of California, San Francisco (UCSF) Institutional Review Board.

METHOD DETAILS

Viruses and Cell Lines

Vero E6 cells were cultured in Dulbecco's Modified Eagle Medium (UCSF Media Production) supplemented with 10% fetal bovine serum (Corning), penicillin/streptomycin (UCSF Media Production) and L-glutamine (Corning) in a humidified incubator at 37°C and 5% CO2. SARS-CoV-2 virus (USA-WA1/2020 strain) was propagated in Vero E6 cells. The Vero E6 cells were infected with the SARS-CoV-2 virus (Wuhan-Hu-1; GenBank accession number MN908947.3, Delta variant B.1.617.2, GenBank: OW998779.1, Omicron BA.1/2021, complete genome GenBank: OP090659.1), incubated at 37°C, 5% CO2 and after 72h the supernatant was collected. The virus was aliquoted and stored at -80 °C. This work was done under Biosafety Level 3 (BSL-3) conditions.

Determination of Virus Titers using Plaque Assay

Viral titer was quantified using a plaque assay in Vero E6 cells. 10-fold dilutions of the virus stock were added to Vero E6 cells in a 12-well plate for 1 hour, after which an overlay of 1.25% Avicel RC-591 in DMEM (UCSF Media Production) was added. The cells were incubated at 37°C, 5% CO2 for 72 hours. The cells were fixed with 10% formalin, stained with crystal violet, and washed with water. The plaques were counted to determine the titer of the virus stock. All work was done under BSL-3 conditions.

H1N1/PR8 1 (gift from Dr. Yoshihiro Kawaoka) was propagated in serum pathogen-free fertilized chicken eggs (Charles River) as previously described 2. In brief, freshly fertilized eggs were kept in an automatic egg turner for 10 days before injection of the virus into the allantoic cavity. Embryos were incubated with the virus for 2 days. Allantoic fluid was harvested and snap-frozen in liquid nitrogen. Titers were determined with a hemagglutination assay. This work was done under BSL-2 conditions.

Airway Organoid Cultures

Organoids were generated from non-tumor lung tissue obtained from patients undergoing lung resection or from donor organs, using a protocol adapted from Sachs and colleagues (Sachs et al., 2019). The Roose lab has IRB Exempt certification (IRB #: 12-09467). Airway tissue was washed 2X with DPBS, placed in sterile petri dishes (Corning® Gosselin™) and filled with 10 ml of digestionbuffer (DMEM/F12, Collagenase I 1.5mg/ml (Thermo Fischer Scientific, cat#17100017), HEPES (UCSF cell culture facility), penicillin 10,000 IU/mL (Thermo Fischer Scientific Cat#15140122). The tissue chunk was minced in 1-3mm pieces using a scalpel blade and transferred with the digestion buffer into a 50 ml Falcon tube. After 1 hour of incubation at 37°C with shaking at 125 rpm, the digested tissue was filtered through a 100μM filter and transferred into a 50 ml Falcon tube. Cells were pelleted (600g, 5 minutes at 4°C) and washed twice with DPBS (UCSF Cell Culture Facility). Airway cell pellet was then resuspended in 1300 μL Matrigel (Corning Cat#356230) and plated in ~50 μL droplets in a 24 well tissue culture plate. Plates were placed at 37°C with 5% CO2 for 20 min to solidify the Matrigel droplets upon which 550μL of airway organoid media was added to each well.

Plates were incubated in the standard tissue culture incubator at 37°C. Images were taken on Days 10 to 21 using a BZX700 inverted microscope with a CCD cooling camera and BZ-X analysis software (KEYENCE).

Airway Organoid Biobank

After 1 to 3 weeks of growth, organoids were dissociated into single cells using TrypLE express. Cells were washed twice with DPBS, pelleted and resuspended in freezing media (Gibco™ Recovery™ Cell Culture Freezing Medium -1560446) and transferred immediately in the Mr. Frosty™ Freezing Container (Cat 51100-001) in the -80C Freezer. Frozen vials were then transferred to the Liquid Nitrogen freezer. When needed, cells were quickly thawed using the 37°C water bath, and transferred to 15ml tubes filled with DPBS. Cells were pelleted (600g, 5 minutes at 4°C) and washed twice with DPBS. Airway cell pellets were then resuspended in 1300 µL Matrigel (Corning) and plated in ~50 µL droplets in a 24 well tissue culture plate. Plates were placed at 37°C with 5% CO2 for 20 min to solidify the Matrigel droplets upon which 550µL of airway organoid media was added to each well. Plates were incubated in a standard tissue culture incubator at 37°C.

TSPAN8 and CD9 plasmid

Plasmids were generated by the Core Facility "Signaling Factory" of the University of Freiburg. CD9 and mCherry sequences were inserted into the pcDNA3.1 Hygro (+) plasmid digested using XhoI. The direction on insert was controlled by sequencing. To generate Tspan8-Cherry, pcDNA3.1 containing hTspan8 and pcDNA3.1-CD9-Cherry were digested using BshTI and NotI and Tspan8 insert was ligated with the Cherry-containing plasmid. Digestion and sequencing analysis were used to select colonies containing correct fusion constructs.

HEK cell lines generation

HEK293T and ACE2+ HEK293Twere transfected with 1ug of either an empty mCherry backbone, TSPAN8-mCherry or CD9 mCherry (provided by Irina Nazarenko) and 1ug of pEF/myc-his B (Addgene) to confer blasticidin (BSD) resistance. Cells were transfected using Fugene (Promega E2691) in Opti-MEM according to the manufacturer's instructions. Following transfection, cells were cultured with BSD

(4ug/ml for HEK293T cells and 8µl/ml for ACE2+ HEK293T cells). Stable clones were sorted for mCherry+ cells and replated in culture media with the appropriate amount of BSD.

SARS-CoV-2 Delta and Omicron Generation

Blood samples were obtained after written informed consent from adults with PCR-confirmed SARS-CoV-2 infection who were enrolled in a prospective cohort study approved by the Biomedical Research Ethics Committee at the University of KwaZulu–Natal (reference BREC/00001275/2020).

BA.1 and BA.1.1 isolate: The Omicron/BA.1 was isolated from a residual swab sample with SARS-CoV-2 isolation from the sample approved by the University of the Witwatersrand Human Research Ethics Committee (HREC) (ref. M210752) as described in Cele *et al* (Cele et al., 2022).

Pseudotyped virus generation

293T cells were transfected with plasmid DNA (per 15cm2 plate: 3.4µg ng of spike mutants, 10 µg pspAX2), and 10 µg Luciferase reporter). Cell culture media was replaced after 16-24hr, then added fresh complete media (DMEM, 10% Serum, P/S, L-Glut). Supernatant containing pseudovirus particles was collected at 48hr after transfection, filtered (0.45 µm). The collected pseudovirus was concentrated on 20% sucrose cushion spinning at 24000RPM for 1hr at 4°C using SW28 rotor. The concentrated was reconstituted in complete DMEM media (5X times concentrated) quantified with a p24 assay (Takara #632200).

Airway Organoid Infection

Airway organoids were removed from Matrigel following 1 minute incubation with Dispase 0.5U/ml (StemCell Technologies, Cat #07913), carefully transferred into a 15 ml tube, washed with DPBS (UCSF Cell Culture Facility) supplemented with 5 mm EDTA (Cellgro, Cat#46-034-CI) and pelleted (200g, 3 minutes at Room Temperature). Organoid pellets were resuspended in 500 µl of airway organoid media. Virus was added at MOI=0.15 for H1N1/PR8 mCherry and MOI=0.3 for SARS-CoV-2. Every organoid well contained 10,000 cells. For mock infections, organoids go through the same process and handling without exposure to viral particles. After 2 hours of incubation, airway organoids were washed twice with PBS and resuspended in 300µL

Matrigel (Corning) and plated in ~50 µL droplets in a 24 well tissue culture plate. Plates were placed at 37°C with 5% CO_2 for 20 min to solidify the Matrigel droplets upon which 550µL of airway organoid media was added to each well. Plates were incubated in the standard tissue culture incubator at 37°C for 92 hours. After 72 hours spectral flow cytometry analyses (CYTEK Aurora) and Confocal Analyses (Zeiss, SP8) were performed.

TSPAN8- and ACE2- blocking antibody assays

Airway organoids were removed from Matrigel following a 1-minute incubation with Dispase 0.5U/ml (StemCell Technologies, Cat #07913), carefully transferred into a 15ml tube, washed with DPBS (UCSF Cell Culture Facility) supplemented with 5 mm EDTA (Cellgro, Cat#46-034-CI) and pelleted (200g, 3 minutes at Room Temperature). Organoid pellets were resuspended in 500 µl of airway organoid media with isotype control RatgG2B Isotype control (R&D Systems, Cat#MAB0061), humanTSPAN8 blocking antibody (R&D Systems, #MAB4734-SP) and/or human ACE2 (R&D Systems, #AF933-SP) at 50µg/ml. After 1 hour incubation with the antibody, SARS-CoV-2 was added. After 2 hours of incubation with the virus, airway organoids were washed twice with PBS and resuspended in 300µL Matrigel (Corning) containing human TSPAN8 blocking antibody (R&D Systems, #MAB4734-SP) and/or human ACE2 (R&D Systems, #AF933-SP) at 50µg/ml. and plated in ~50 µL droplets in a 24 well tissue culture plate. Plates were placed at 37°C with 5% CO_2 for 20 min to solidify the Matrigel droplets upon which 550µL of airway organoid media was added to each well. Plates were incubated in the standard tissue culture incubator at 37°C for 24 to 72 hours. Spectral flow cytometry analyses (CYTEK Aurora) and Confocal Analyses (Zeiss, SP8) were performed.

Spectral Flow analysis

Organoids were dissociated into single cells using TrypLE express (ThermoFisher Scientific Cat#12604012). Single Cell suspensions were transferred to 2 ml cryovial tubes and washed twice with FACS buffer (2% FBS, 0,5% BSA, DPBS, 0.1mg Liberase + Y-27632 5mM (Abmole) before incubating for 10 minutes with Ig block (True-Stain Monocyte Blocker™ Innovex Bioscience). Cells were incubated next with Zoombie NiR (Biolegend) and the antibody mix for 35 minutes followed by two washes

with FACS Buffer. Cells were fixed for 30 minutes with Permeabilization Fixation Buffer (eBioscience) washed twice and for 45 minutes with antibodies staining for intracellular targets followed by a 20-minute incubation with secondary conjugated antibodies. Cells were washed with DPBS (UCSF facilities) before acquiring in the CYTEK.

Antibodies used for spectral flow cytometry: TSPAN8 AF405, clone 45811(R&D systems), CD66c (CEACAM6) BV510, clone B6.2 (BD Bioscience), EPCAM BV711, clone 9C4, Biolegend; CD86 BV650, clone IT2.2 (BioLegend), CD117 BV785, clone 104D2, BioLegend, CD49f PE, clone GoH3 (BioLegend), CD80 PE-Cy5, clone 2D10 (BioLegend), CD271 PE-Cy7, clone ME20.4, BioLegend, ACE2 AF647, clone Q9BYF1 (R&D systems), CEACAM5 APC-Fire750, clone 487609 (R&D systems), MUC5AC Biotin, clone 45M1 (ThermoFischer Scientific), Streptavidine APC (eBioscience), anti-TMPRSS2, clone EPR3862 (Abcam), acetylated Tubulin AF488, clone 6-11B-1 (SantaCruz Biotechnologies), anti-dsRNA (Scicons), IGg BV605, clone4053 (Biolegend).

Confocal Imaging

Airway organoids were removed from Matrigel following 1 minute incubation with Dispase 0.5U/ml (StemCell Technologies, Cat #07913), carefully transferred into a 15 ml tube, washed with DPBS (UCSF Facility) and pelleted (200g, 2 minutes at Room Temperature). The organoid pellet was incubated for 30 minutes in 4% PFA 10% FBS Triton 0.1X and TrueStain. Organoids were washed twice with PBS-Triton 0.1X and stained overnight at 4°C with primary antibodies dsRNA Ab (clone J2, Scicons), SARS-CoV-2 Nucleocapsid Ab (Novus Biologicals, Cat#NB100-56576SS). Organoids were then washed and incubated for two hours with secondary antibodies Goat anti mouse IGg AF555, clone4053 (Biolegend), CD49f PE, clone GoH3 (BioLegend), ACE2 AF647, clone Q9BYF1 (R&D systems), and acetylated Tubulin AF488, clone 6-11B-1 (SantaCruz Biotechnologies). After staining, organoids were washed with DPBS- 0.1X Triton and resuspended in Fructose-Glycerol Clearing Solution 60% (vol/vol) glycerol and 2.5 M fructose. Organoids were mounted on coverslips and imaged with Leica SP8 Confocal Microscopy. Confocal Z-stack images were generated using the staining maxima. For the images in the figure panels, Z-stacks with 0.5-µm separation were acquired and all Z-stacks were combined to a z-stack

projection throughout the entire organoid imaged. Alternatively, to highlight detail such as staining of ciliated cells by means of acTUBA, only 3 Z-stacks with most in-focus acTUBA staining were combined to create a z-projections with just 3 z-stacks per image.

Single cell and library preparation for scRNA-sequencing

For the single-cell RNA sequencing experiments each organoid was generated from 4 different donors. Airway organoids were removed from Matrigel following 1 minute incubation with Dispase 0.5U/ml (StemCell Technologies, Cat #07913), carefully transferred into a 15 ml tube, washed with DPBS (UCSF Cell Culture Facility) supplemented with 5 mm EDTA (Cellgro, Cat#46-034-CI) and pelleted (200g, 3 minutes at Room Temperature). Organoid pellets were resuspended in 500 µl of airway organoid media. Virus was added at MOI=0.3 for SARS-CoV-2 and the same amount of DMEM was added in the Mock condition. Every organoid well contained 10,000 cells. The experiments was performed in triplicates for each condition. After 2 hours of incubation, airway organoids were washed twice with PBS and resuspended in 300µL Matrigel (Corning) and plated in ~50 µL droplets in a 24 well tissue culture plate. Plates were placed at 37°C with 5% CO2 for 20 min to solidify the Matrigel droplets upon which 550µL of airway organoid media was added to each well. Plates were incubated in the standard tissue culture incubator at 37°C for 72 hours. After 72 hours organoids were dissociation into single-cell suspension using TrypLE express (ThermoFisher Scientific Cat#12604012), cells were counted, and the same conditions were pooled together (78,000 total cells). Pooled cells were then loaded evenly across two lanes in the Chromium Controller for generating single-cell contained in lipid droplets following the manufacturer’s instructions (10X Genomics). A Chromium Single cell 3’ Reagent Kit (v3.1) (10X Genomics) was used for reverse transcription, cDNA amplification and library construction of the gene expression libraries following the manufacturer’s instructions. All samples were encapsulated, and cDNA was generated within 6 hours after organoid processing. Finally, Pooled libraries were sequenced on an Illumina NovaSeq 6000

QUANTIFICATIONS AND STATISTICAL ANALYSES

Single cell RNAseq analysis

Data pre-processing of 10x Genomics Chromium scRNA-seq data:

Data pre-processing was performed as previously described[16]. Briefly, sequencer-obtained bcl files were demultiplexed into individual samples using the Cellranger (v.3.0.2, 10X Genomics) suite of tools (https://support.10xgenomics.com). Feature-barcode matrices were obtained for each sample by aligning the raw fastqs to GRCh38 reference genome (annotated with Ensembl v85) using the Cellranger count. Raw feature-barcode matrices were loaded into Seurat (v.4.0.3)[17] and genes with fewer than 3 UMIs were dropped from the analyses. Matrices were further filtered to remove events with greater than 30% percent mitochondrial content, events with greater than 50% ribosomal content, or events with fewer than 250 total genes. The cell cycle state of each cell was assessed using a published set of genes associated with various stages of human mitosis.

Inter-sample doublet detection:

Inter-sample doublet detection was performed as previously described[16]. Libraries containing samples pooled prior to loading were processed using Freemuxlet (https://github.com/statgen/popscle) to identify clusters of cells belonging to the same patient via SNP concordance. Cells are classified as singlets arising from a single library, doublets arising from two or more libraries, or as ambiguous cells that cannot be accurately assigned to any existing cluster (due to a lack of sufficient genetic information).

Data quality control and Normalization:

The filtered count matrices were normalized, and variance stabilized using negative binomial regression via the scTransform method offered by Seurat[17]. The effects of mitochondrial content, ribosomal content, and cell cycle state were regressed out of the normalized data to prevent any confounding signal. The scTransformed data from different sequencing libraries were combined and normalized using Harmony integration software[15]. The merged object, after final QC, contained 21,225 single cells.

Intra-sample heterotypic doublet detection:

All libraries were further processed to identify heterotypic doublets arising from the 10X sample loading. Processed, annotated Seurat objects were processed using the DoubletFinder package[12]. Briefly, the cells from the object are modified to generate artificial duplicates, and true doublets in the dataset are identified based on similarity to the artificial doublets in the modified gene space. The prior doublet rate per library was approximated using the information provided in the 10x knowledgebase (https://kb.10xgenomics.com/hc/en-us/articles/360001378811) and this was corrected to account for homotypic doublets using the per-cluster numbers in each dataset.

Differential expression tests and cluster marker genes, cluster annotation:

Differential gene expression (DGE) tests were performed on log-normalized gene counts using the Poisson test (with a latent batch variable to account for multiple library preparations) as implemented in the FindMarkers/FindAllMarkers functions in Seurat. Cluster marker gene lists were generated by applying the Poisson test to identify upregulated genes for one cluster against all other clusters in the dataset. The resulting top ranked genes (sorted by log-fold change) for each cluster and canonical markers[13] were used for cluster annotation (Fig. 3, Supp. Fig. S6).

Data Mining

To examine presence of *ACE2* and *TSPAN8* co-expressing cells in vivo in normal human airway, we analyzed the processed 10X single-cell sequencing data from Travaglini et al(Travaglini et al., 2020)[3]. Of the 60,993 cells derived from airway tissue of 3 patient donors in this dataset, 48 cells were found with at least 1 UMI (unique molecular identifier) for both genes. Only 1 cell derived from patient 1, which had fewer cells sequenced overall and so we excluded it. The expression values represent ln(UMI-per-10K + 1) in each of the 47 cells from patients 2 and 3. Cell type designations were determined by Travaglini *et al*. Differential expression of TSPAN8 in nasal swabs of adult patients with acute respiratory illness (ARI) due to COVID-19 (n=93) or other viral infection (n=41), in comparison to patients with ARI due to non-viral etiology (n=100), was derived from Mick et al(Mick et al., 2020). The differential expression analysis between the 3 viral status groups was performed with the R package limma while controlling for gender and age.

Statistics

Statistical analyses were run in R (version 4.0.2) (quote 1). Paired Samples Wilcoxon Test were performed using Wilcox.Test (stat v4.0.2) (quote 1). Outliers were removed if the value was over Q3+1.5 IQR. We used PCA (FactoMineR v2.4) to perform Principal Component Analysis and fviz_pca_ind or fviz_pca_var (factoextra v1.0.7) (quote 3) for visualization. Community distances were evaluated based on relative abundance of cell populations by PERMANOVA (Bray, *p<0.05). For spearman's and pearson's correlations, we used cor function (stat v4.0.2) to compute the correlation coefficients and lm (stat v4.0.2) to fit linear models. Data management was done using tidyverse (v1.3.0) (quote 4). All graphs were built using ggplot2 (quote 5) (Kassambara and Mundt, 2017; Lê et al., 2008; Team, 2013; Wickham et al., 2016; Wickham et al.). UMAP dimensionality reduction, discovery of upregulated/downregulated genes, and gene expression related plots were constructed in R v 4.0.3 (1) via Seurat v 4.0 provided by the Satija Lab (Hao et al., 2020) and ggplot2 v 3.3.3 Clusters were separated using the Louvain clustering method with a resolution of 0.6, and upregulated differential expression gene scores between clusters were used to establish cell type identities(Wickham et al., 2016).

Bar graph of enrichment analysis up regulated pathway and processes based in cells positive for SARS-CoV-2 reads were generated using Metascape(Zhou et al., 2019).

scRNA-seq specific Statistical Analysis:

All statistical analyses were performed using R. Dot plots were generated using Seurat's 'DotPlot' function. Differentially expressed genes were identified using the Poisson test (with a latent batch variable to account for multiple library preparations) as implemented in the FindMarkers/FindAllMarkers functions in Seurat.

Software

GraphPad Prism 9 was used for DATA visualization. Fiji/ImageJ (V) software 2.1.0/1.53c was used for confocal microscopy analysis. FlowJO and CYTEK Aurora software 10.7.1 was used for spectral flow analyses.

References

Cele, S., Jackson, L., Khoury, D.S., Khan, K., Moyo-Gwete, T., Tegally, H., San, J.E., Cromer, D., Scheepers, C., and Amoako, D.G. (2022). Omicron extensively but incompletely escapes Pfizer BNT162b2 neutralization. Nature *602*, 654-656.

Hao, Y., Hao, S., Andersen-Nissen, E., Mauck, W.M., Zheng, S., Butler, A., Lee, M.J., Wilk, A.J., Darby, C., and Zagar, M. (2020). Integrated analysis of multimodal single-cell data. bioRxiv.

Kassambara, A., and Mundt, F. (2017). Package 'factoextra'. Extract and visualize the results of multivariate data analyses *76*.

Lê, S., Josse, J., and Mazet, F. (2008). Package 'FactoMineR. J Stat Softw [Internet] *25*, 1-18.

Mick, E., Kamm, J., Pisco, A.O., Ratnasiri, K., Babik, J.M., Castañeda, G., DeRisi, J.L., Detweiler, A.M., Hao, S.L., and Kangelaris, K.N. (2020). Upper airway gene expression reveals suppressed immune responses to SARS-CoV-2 compared with other respiratory viruses. Nature communications *11*, 1-7.

Ross, J.T., Nesseler, N., Lee, J.W., Ware, L.B., and Matthay, M.A. (2019). The ex vivo human lung: research value for translational science. JCI Insight *4*.

Sachs, N., Papaspyropoulos, A., Zomer-van Ommen, D.D., Heo, I., Bottinger, L., Klay, D., Weeber, F., Huelsz-Prince, G., Iakobachvili, N., Amatngalim, G.D., *et al.* (2019). Long-term expanding human airway organoids for disease modeling. EMBO J *38*.

Team, R.C. (2013). R: A language and environment for statistical computing.

Travaglini, K.J., Nabhan, A.N., Penland, L., Sinha, R., Gillich, A., Sit, R.V., Chang, S., Conley, S.D., Mori, Y., and Seita, J. (2020). A molecular cell atlas of the human lung from single-cell RNA sequencing. Nature *587*, 619-625.

Wickham, H., Chang, W., Henry, L., Pedersen, T., Takahashi, K., Wilke, C., Woo, K., Yutani, H., and Dunnington, D. (2016). Springer-Verlag. New York.

Wickham, H., Francois, R., Henry, L., and Müller, K. others. 2015.". Dplyr: A Grammar of Data Manipulation" R Package Version 04 *3*.

Zhou, Y., Zhou, B., Pache, L., Chang, M., Khodabakhshi, A.H., Tanaseichuk, O., Benner, C., and Chanda, S.K. (2019). Metascape provides a biologist-oriented resource for the analysis of systems-level datasets. Nature communications *10*, 1-10.

Supplementary Figures Legends

Supplementary Figure 1: Donor-derived airway organoids display distinct but stable composition.

A) Brightfield images of organoids derived from the upper and lower region of the left airway of individual L2. Scale bars=200µm. Pie charts representing the distribution of cell populations found in airway organoids from the upper and lower lobes of subjects L2 and L7. B) Pie charts representing the distribution of cell populations in airway organoids from different donors analyzed by Spectral flow of different passages. In A and B, each section of the pie chart represents the cell population mean from 3 independent experiments with at least 3 replicates for the distinct donor-derived organoids. C) Table of 20 biobanked airway organoids from 20 different subjects. Indicated are the resource qualities of this biobank with indicated cryovial amount and passage number. Normal adjacent lung tissue was collected from non-small cell lung cancer patients; 2425LL, 2450UL, 2477UL, 2478UL, 2520LL, 2521UL, 2522UL, 2524UL, 2525UL, 2526UL, 2527UL, 2531UL, 2547UL, 2551ML. Alternatively, lung samples were obtained from donor lungs rejected for or left over from transplantation, received from organ procurement organization (Donor Network West) as previously described (Ross et al., 2019); samples L1, L2, L3, L5, L6, and L7.

D) TMPRSS2+ cells fraction in airway organoids based on spectral flow cytometry analyses. Bars represent mean, error bars are SEM. 3 independent experiments with at least 3 replicates for the distinct donor-derived organoids were performed. One way non-parametric ANOVA was performed to compare if there is differences between individual organoids.E) ACE2+ cells fraction in airway organoids based on spectral flow cytometry analyses. Bars represent mean, error bars are SEM. Experiments were repeated 3-5 times with at least 3 replicates each. One way non-parametric ANOVA was performed to compare if there is differences between individual organoids.

F) Spectral flow cytometry gating strategy. Cell populations are defined after excluding ZombieNIR-positive, dead cells and doublets in steps a-c, using the indicated gating strategies. (1) acTUBA + CD271- are considered ciliated cells. Fraction (d) is analyzed for MUC5AS and TSPAN8 and (2) MUC5AC+ acTUBA- cells are considered goblet-like cells. (3) TSPAN8+ MUC5AC- acTUBA- are defined as pre-goblet cells. Fraction (e) is analyzed for cKit and (g) are considered cKit-positive cells. Fraction (f) is analyzed for CD49f and CD271, (4) CD49f+ CD271-, (5) CD49f+ CD271+ and (6)

CD49f- CD271+ are considered basal stem cells and (7) CD49f- CD271- are undefined cells. G) Spectral flow cytometry gating strategy for mCherry+ cell populations. The same gates shown in Supplementary 1 A) were drawn on live, single cell and mCherry+ cells (h).

Supplementary Figure 2. SARS-CoV-2 infection in airway organoids.

A) Pie charts representing distribution of cell populations in organoids Mock, infected with SARS-CoV-2 -WA-1, at 72h p.i. (MOI=0.3) for 5 independent experiments. The pie chart fraction represents the mean of the cell populations for every independent experiment. B) Spectral flow analyses of the percentage of cells positive for CEACAM6, CEACAM5, CD80, CD86 and ACE2 at 72h p.i. (MOI=0.3) in live cells of Mock and SARS-CoV-2 WA-1 infected organoids. For SARS-CoV-2 infected organoids, the fraction of ACE2+ cells (or other marker-positive cells) is shown in non-infected cells (dsRNA-) or infected cells (dsRNA+) condition. Each dot represents the mean value of every experiment. Bars represent mean, error bars are SEM. Paired t-test, *p < 0.05, ****p<0.0001; ns, non-significant.

C) Pie charts representing the distribution of each cell population in Mock and SARS-CoV-2-WA-1 infected organoids at 72h p.i. (MOI=0.3). Each fraction of the pie chart represents the mean value of 3 independent experiments of the specific cell population in distinct donor-derived airway organoids. D-F) Box and whisker plots representing the spectral flow analyses of the % of (D) goblet-like cells (Muc5AC+), (E)CD49f- CD271- , (F) CD49f+ or CD271+in Mock and SARS-CoV-2-WA-1 infected organoids. Each dot represents the mean of % cell type for 3 independent experiments with 3-5 replicates per experiment for the distinct donor. Wilcoxon signed-rank paired test,*p<0.05. G) Quantification of percentage relative decrease in cell yield post-infection as a comparison to the cell number cell prior to the infection; for Mock and infected organoids at 72h post H1N1/PR8 infection (MOI=0.15). H1N1/PR8 virus did not induce increased levels of cell death, compared to mock infection Bars represent mean, and error bars are SEM, n=3 to n=5. Paired t-test, *p＜0.05; ns, non-significant. in mock and SARS-CoV-2-WA-1 infected organoids (MOI=0.3, 72h p.i.). Each dot represents the mean value of the percentage of the cell population in distinct donor-derived airway organoids for every independent experiment. 3 independent experiments with at least 3 replicates for the distinct donor-derived organoids were

performed.

Supplementary Figure 3. Donor-derived airway organoid responses to SARS-CoV-2.

A) Principal component analysis (PCA) using cell populations and infection conditions (all SARS-CoV-2-WA-1). PCA reduced the descriptors into two dimensions as shown in the individual plot. Each point corresponds to an observation in the dataset and were colored according to their mock (blue) or infection (red) state. Community distances were evaluated based on relative abundance of cell populations by PERMANOVA (Bray, *$p<0.05$). The PCA showed that 2525UL components change very minor to SARS-CoV-2 infection, while 2450UL and 2522UL reveal robust component alterations to SARS-CoV-2 infection. 3 independent experiments with at least 3 replicates for the distinct donor-derived organoids were performed. B-G) Scatter Plot showing the relationship between SARS-CoV-2-WA-1 infection rate and fraction B) CD86+ cells, C) cKit+ cells, D) MUC5AC+ cells, E) acTUBA+ cells prior to infection, F) Age of or (G) ACE2 the adult stem cell donor. Each dot represents the mean of % cell type in 3 independent experiments with at least 3 replicates for the distinct donor-derived organoid. Linear regression, R2 value and Pearson Correlation statistical significance are stated on the graph. Every dot shows the mean of the fraction of the specific cell in 3 independent experiments with 3-5 replicates each. Functions of the positive or negative correlations are depicted by the Linear Model with R2 as value to indicate how well the linear model function agrees with the individual data points. If R2=0 then 0% of the data points follow the linear model, if R2=0.5 then 50% of the data points follow the linear model, and if R2=1 then 100% of the data points follow the linear model. H-L) Box and whisker plots representing the percentage of (G) CD86+ cells, (I) CD80+ cells, (K) CEACAM5+ and (L) CEACAM6+cells analyzed by spectral flow cytometry for 12 Mock and SARS-CoV-2-WA-1 infected organoids. Each dot represents the mean of % cell type for the distinct donor. 3 independent experiments with at least 3 replicates for the distinct donor-derived organoids were performed. Wilcoxon signed-rank test, *$p<0.05$, N=12, n=3. Outliers were removed if the value was over Q3+1.5 IQR.

Supplementary Figure 4: scRNAseq analyses in airway organoids

A) Dot plot of canonical and top differentially expressed genes used to determine cluster annotations established in Fig 5A. B) Cells negative for SARS-CoV-2 reads (orange) and positive for SARS-CoV-2 reads (blue) overlay on the UMAP from Fig 5A. A sample of 100 randomly selected cells each for SARS-CoV-2 negative (orange) and SARS-CoV-2 positive (blue) populations selected for subsequent DGE analysis. Image is representative of independent random samples.

C) Cells positive for *TSPAN8, ACE2, TMPRSS2, NRP1, FURIN, CSTL1, CD24, AREG, CD9, CD37*, nCountSARS-CoV-2 reads and log (base 2) SARS-CoV-2 read counts (orange) overlaid on the UMAP seen in Fig 5A. Neutralizing antibodies from COVID-19 patients have multiple targets (Brouwer et al., 2020; Chi et al., 2020), suggesting that protective immune responses occur that may block interactions of molecules other than the S protein-ACE2 receptor pair. In our scRNA data *RALA* and *CD24* reads were present in infected cells. RALA (RAS like Proto-Oncogene A) was identified in the host-coronavirus protein network (Gordon et al., 2020) and infection levels of a hepatoma cell line is reduced when deleted by CRISPR(Wang et al., 2021a). CD24 is a glycosyl-phosphatidyl-inositol (GPI)-anchored membrane protein that can repress the host response to DAMPS (Chen et al., 2009) and EXO-CD24 exosomes are explored in clinical trials as therapy for COVID19 patients (Clinical Trial NCT047477574). Studies using cell lines had also implicated additional mediators of infection, including AXL (Wang et al., 2021b) CD147 (Shilts et al., 2021; Wang et al., 2020) and neuropilin-1 (NRP1) (Cantuti-Castelvetri et al., 2020; Daly et al., 2020).

Top differentially upregulated genes, including *TSPAN8* (arrow), within representative random subsample of SARS-CoV-2 positive (top) versus SARS-CoV-2 negative (bottom) cells. Circle sizes indicate the percentage of cells within the total cell population that the specific gene is expressed in. We did not observe CD9 or any other Tetraspanin enriched in infected cells (See Supplemental Tables).

Supplementary Figure 5: TSPAN8 correlates with infection rate in airway organoids.

A) Box and whisker plots representing the percentage of TSPAN8+ cells analyzed by spectral flow cytometry for each distinct donor in Mock and H1N1/PR8 infected organoids (MOI=0.15, 72h p.i.). Every dot represents the mean of cell percentage for 3 independent experiment in that distinct donor.

B) Spectral flow cytometry plots of overlay between dsRNA- cells (in gray) and

dsRNA+ cells (in red) in the most infected SARS-CoV-2-WA-1 infected organoids at 72h p.i. (MOI=0.3). X axis represents TSPAN8 expression intensity and Y axis represents ACE2 expression intensity.

C) Scatter Plot showing the relationship between SARS-CoV-2-WA-1 infection rate and ACE2+ TSPAN8+ cells prior to infection, prior to infection. Each dot represents the mean of 3 independent experiment for the distinct donor. Linear regression, R2 value and Pearson Correlation statistical significance stated on graph. Mean values, N= 3, n=3 for every donor-derived organoid.

Supplementary Figure 6: TSPAN8 is a facilitator of SARS-CoV-2

A) Spectral Flow histogram plot of ACE2 (left) and mCherry (right) intensity in ACE2 mCherry, ACE2 CD9 and ACE2 TSPAN8 HEK293T cells.

B) Median Fluorescence Intensity (MFI) of ACE2 in ACE2 mCherry, ACE2 CD9 and ACE2 TSPAN8 HEK293T cells measured by Spectral flow.

C) Scheme of pseudo viruses generated and utilized.

D) Luminescence measured as a function of pseudovirus entry for the backbone Ps. PsVirus expressing spike of WA1 (D614G)-, Delta VOC, and Omicron VOC as well as VSV-G were tested at 48hours post Psvirus infection.

E) Mutations found on the Spike protein of Delta and Omicron Strain of SARS-CoV-2 (left) and workflow of airway organoids with SARS-CoV-2 strains (right). In the second half of 2020, SARS-CoV-2 VOCs with a combination of several mutations emerged, including Alpha, first described in southeast England(Volz et al., 2021), and Beta, first identified in South Africa(Tegally et al., 2021). In February–March 2021, Alpha rapidly became the prevailing variant in many regions of the world and a higher reproduction number was inferred from early epidemiological data (Davies et al., 2021; Wagar et al., 2021; Washington et al., 2021). Beyond S(D614G), Alpha has 18 further mutations in its genome compared with the progenitor, with two deletions and six substitutions within the *S* gene The Delta SARS-CoV-2 variant, B.1.617.2 accumulated eight amino acid mutations in the S protein, including T19R, G142D, FR156-157del, R158G, L452R, T478K, P681R, D950N (Harvey et al., 2021).

F) Viral titers generated from Vero E6 cells infected with SARS-CoV-2-WA-1, -Delta and -Omicron Strains (5 days p.i., MOI=0.01).

G) Percentage of SARS-CoV-2 Nucleocapsid+ in live mCherry, TSPAN8 and CD9 expressing HEK293T cell at 48h post SARS-CoV-2 WA-1 or Delta infection. H)

Absolute number of live cells ACE2 mCherry-, ACE2 TSPAN8- and ACE2 CD9-expressing HEK293T cell at 24h post-SARS-CoV-2 WA-, -Delta, or -Omicron infection (MOI=0.3); at least 3 independent experiments with 5 replicates each were performed.

Supplementary Figure 7: Infection of airway organoids by SARS-CoV-2 VOCs

A) Pie charts representing distribution of cell populations in organoids Mock, infected with SARS-CoV-2-WA-1, Delta and Omicron at 72h p.i. (MOI=0.3). The pie chart fraction represents the mean of the cell populations for 3 independent experiments with at least 3 replicates.

B-F) Percentage of B) acTUBA+ cells, C) cKit+ cells, D) TSPAN8+ MUC5AC- , E) CD271+ or CD49f+ cells, F) CD86+ cells in in AO Mock or infected with SARS-CoV-2 -WA-1, -Delta or -Omicron at 72h p.i. (MOI=0.3), 3 independent experiments with 3 replicates for the distinct donor-derived organoids were performed; non parametric ANOVA tested corrected by Geisser Greenhouse Correction *p<0.05, **p<0.001, ***p<0.001.

G) Stack bar-charts representing of dsRNA+ cells (in red) in SARS-CoV-2 infected organoids (left) and the fraction of cell types infected by SARS-CoV-2 WA-1, -delta or -omicron (dsRNA+) at 72h p.i., MOI=0.3 Every fraction of the stack bar chart shows the mean of the distinctive cell population for every single independent experiment for the same donor, 3 independent experiments with 5 replicates each.; non parametric ANOVA tested corrected by Geisser Greenhouse Correction *p<0.05, **p<0.001, ***p<0.001.

H) Percentage of ACE2+ cells in 2522 and 2450 airway organoids Mock or infected with SARS-CoV-2 -WA-1, -Delta or -Omicron at 72h p.i. (MOI=0.3), , 3 independent experiments with 5 replicates each, nonparametric ANOVA tested corrected by Geisser Greenhouse Correction *p<0.05, **p<0.001, ***p<0.001.

I) As in S8H but for TSPAN8+ cells in 2522 and 2450 airway organoids.

J-K) Spectral flow cytometry layouts (J) and quantification (K) at 72h post SARS-CoV-2 -WA-1 infection of dsRNA-positive cells in organoids pre-treated with TSPAN8 and/or ACE2 blocking antibody (50µg/ml); representative data from 2 independent experiment with 4 replicates each.

References

Barberis, E., Vanella, V.V., Falasca, M., Caneparo, V., Cappellano, G., Raineri, D., Ghirimoldi, M., De Giorgis, V., Puricelli, C., and Vaschetto, R. (2021). Circulating exosomes are strongly involved in SARS-CoV-2 infection. Frontiers in molecular biosciences *8*, 29.

Bastard, P., Gervais, A., Le Voyer, T., Rosain, J., Philippot, Q., Manry, J., Michailidis, E., Hoffmann, H.H., Eto, S., Garcia-Prat, M., *et al.* (2021). Autoantibodies neutralizing type I IFNs are present in ~4% of uninfected individuals over 70 years old and account for ~20% of COVID-19 deaths. Sci Immunol *6*.

Becht, E., McInnes, L., Healy, J., Dutertre, C.A., Kwok, I.W.H., Ng, L.G., Ginhoux, F., and Newell, E.W. (2018). Dimensionality reduction for visualizing single-cell data using UMAP. Nat Biotechnol.

Bonnet, M., Maisonial-Besset, A., Zhu, Y., Witkowski, T., Roche, G., Boucheix, C., Greco, C., and Degoul, F. (2019). Targeting the tetraspanins with monoclonal antibodies in oncology: focus on Tspan8/Co-029. Cancers *11*, 179.

Bonser, L.R., Koh, K.D., Johansson, K., Choksi, S.P., Cheng, D., Liu, L., Sun, D.I., Zlock, L.T., Eckalbar, W.L., and Finkbeiner, W.E. (2021). Flow-cytometric analysis and purification of airway epithelial-cell subsets. American Journal of Respiratory Cell and Molecular Biology *64*, 308-317.

Brouwer, P.J., Caniels, T.G., van der Straten, K., Snitselaar, J.L., Aldon, Y., Bangaru, S., Torres, J.L., Okba, N.M., Claireaux, M., and Kerster, G. (2020). Potent neutralizing antibodies from COVID-19 patients define multiple targets of vulnerability. Science *369*, 643-650.

Cantuti-Castelvetri, L., Ojha, R., Pedro, L.D., Djannatian, M., Franz, J., Kuivanen, S., van der Meer, F., Kallio, K., Kaya, T., and Anastasina, M. (2020). Neuropilin-1 facilitates SARS-CoV-2 cell entry and infectivity. Science *370*, 856-860.

Cele, S., Jackson, L., Khoury, D.S., Khan, K., Moyo-Gwete, T., Tegally, H., San, J.E., Cromer, D., Scheepers, C., and Amoako, D.G. (2022). Omicron extensively but incompletely escapes Pfizer BNT162b2 neutralization. Nature *602*, 654-656.

Chen, G.-Y., Tang, J., Zheng, P., and Liu, Y. (2009). CD24 and Siglec-10 selectively repress tissue damage–induced immune responses. Science *323*, 1722-1725.

Chi, X., Yan, R., Zhang, J., Zhang, G., Zhang, Y., Hao, M., Zhang, Z., Fan, P., Dong, Y., and Yang, Y. (2020). A neutralizing human antibody binds to the N-terminal domain of the Spike protein of SARS-CoV-2. Science *369*, 650-655.

Chua, R.L., Lukassen, S., Trump, S., Hennig, B.P., Wendisch, D., Pott, F., Debnath, O., Thurmann, L., Kurth, F., Volker, M.T.*, et al.* (2020). COVID-19 severity correlates with airway epithelium-immune cell interactions identified by single-cell analysis. Nat Biotechnol *38*, 970-979.

Co, J.Y., Margalef-Catala, M., Li, X., Mah, A.T., Kuo, C.J., Monack, D.M., and Amieva, M.R. (2019). Controlling Epithelial Polarity: A Human Enteroid Model for Host-Pathogen Interactions. Cell Rep *26*, 2509-2520 e2504.

Cobey, S., Larremore, D.B., Grad, Y.H., and Lipsitch, M. (2021). Concerns about SARS-CoV-2 evolution should not hold back efforts to expand vaccination. Nat Rev Immunol *21*, 330-335.

Combes, A.J., Courau, T., Kuhn, N.F., Hu, K.H., Ray, A., Chen, W.S., Clearly, S.J., Chew, N.W., Kushnoor, D., and Reeder, G.C. (2020). Global Absence and Targeting of Protective Immune States in Severe COVID-19. bioRxiv.

Corbiere, V., Dirix, V., Norrenberg, S., Cappello, M., Remmelink, M., and Mascart, F. (2011). Phenotypic characteristics of human type II alveolar epithelial cells suitable for antigen presentation to T lymphocytes. Respir Res *12*, 15.

Daly, J.L., Simonetti, B., Klein, K., Chen, K.-E., Williamson, M.K., Antón-Plágaro, C., Shoemark, D.K., Simón-Gracia, L., Bauer, M., and Hollandi, R. (2020). Neuropilin-1 is a host factor for SARS-CoV-2 infection. Science *370*, 861-865.

Davies, N.G., Abbott, S., Barnard, R.C., Jarvis, C.I., Kucharski, A.J., Munday, J.D., Pearson, C.A., Russell, T.W., Tully, D.C., and Washburne, A.D. (2021). Estimated transmissibility and impact of SARS-CoV-2 lineage B. 1.1. 7 in England. Science *372*, eabg3055.

Earnest, J.T., Hantak, M.P., Li, K., McCray Jr, P.B., Perlman, S., and Gallagher, T. (2017). The tetraspanin CD9 facilitates MERS-coronavirus entry by scaffolding host cell receptors and proteases. PLoS pathogens *13*, e1006546.

Earnest, J.T., Hantak, M.P., Park, J.E., and Gallagher, T. (2015). Coronavirus and influenza virus proteolytic priming takes place in tetraspanin-enriched membrane microdomains. J Virol *89*, 6093-6104.

Escalera, A., Gonzalez-Reiche, A.S., Aslam, S., Mena, I., Laporte, M., Pearl, R.L., Fossati, A., Rathnasinghe, R., Alshammary, H., van de Guchte, A.*, et al.* (2022). Mutations in SARS-CoV-2 variants of concern link to increased spike cleavage and virus transmission. Cell Host Microbe *30*, 373-387 e377.

Fang, S., Wei, J., Pentinmikko, N., Leinonen, H., and Salven, P. (2012). Generation of functional blood vessels from a single c-kit+ adult vascular endothelial stem cell. PLoS Biol *10*, e1001407.

Fiege, J.K., Thiede, J.M., Nanda, H.A., Matchett, W.E., Moore, P.J., Montanari, N.R., Thielen, B.K., Daniel, J., Stanley, E., Hunter, R.C.*, et al.* (2021). Single cell resolution of SARS-CoV-2 tropism, antiviral responses, and susceptibility to therapies in primary human airway epithelium. PLoS Pathog *17*, e1009292.

Finkelshtein, D., Werman, A., Novick, D., Barak, S., and Rubinstein, M. (2013). LDL receptor and its family members serve as the cellular receptors for vesicular stomatitis virus. Proc Natl Acad Sci U S A *110*, 7306-7311.

Firas A. Rabi 1, Mazhar S. Al Zoubi 2, , G.A.K., , D.M.S.a., and 4, A.D.A.-N. SARS-CoV-2 and Coronavirus Disease 2019: What We Know So Far. https://doiorg/103390/pathogens9030231.

Gao, Y., Cai, C., Grifoni, A., Muller, T.R., Niessl, J., Olofsson, A., Humbert, M., Hansson, L., Osterborg, A., Bergman, P.*, et al.* (2022). Ancestral SARS-CoV-2-specific T cells cross-recognize the Omicron variant. Nat Med *28*, 472-476.

Gonzalez, H., Mei, W., Robles, I., Hagerling, C., Allen, B.M., Okholm, T.L.H., Nanjaraj, A., Verbeek, T., Kalavacherla, S., and van Gogh, M. (2022). Cellular architecture of human brain metastases. Cell *185*, 729-745. e720.

Gordon, D.E., Jang, G.M., Bouhaddou, M., Xu, J., Obernier, K., White, K.M., O'Meara, M.J., Rezelj, V.V., Guo, J.Z., Swaney, D.L.*, et al.* (2020). A SARS-CoV-2 protein interaction map reveals targets for drug repurposing. Nature *583*, 459-468.

Gray, T., Coakley, R., Hirsh, A., Thornton, D., Kirkham, S., Koo, J.-S., Burch, L., Boucher, R., and Nettesheim, P. (2004). Regulation of MUC5AC mucin secretion and airway surface liquid metabolism by IL-1β in human bronchial epithelia. American Journal of Physiology-Lung Cellular and Molecular Physiology *286*, L320-L330.

Han, Y., Duan, X., Yang, L., Nilsson-Payant, B.E., Wang, P., Duan, F., Tang, X., Yaron, T.M., Zhang, T., Uhl, S.*, et al.* (2021). Identification of SARS-CoV-2 inhibitors using lung and colonic organoids. Nature *589*, 270-275.

Hantak, M.P., Qing, E., Earnest, J.T., and Gallagher, T. (2019). Tetraspanins: architects of viral entry and exit platforms. Journal of virology *93*.

Hao, Y., Hao, S., Andersen-Nissen, E., Mauck, W.M., Zheng, S., Butler, A., Lee, M.J., Wilk, A.J., Darby, C., and Zagar, M. (2020). Integrated analysis of multimodal single-cell data. bioRxiv.

Harvey, W.T., Carabelli, A.M., Jackson, B., Gupta, R.K., Thomson, E.C., Harrison, E.M., Ludden, C., Reeve, R., Rambaut, A., Consortium, C.-G.U., *et al.* (2021). SARS-CoV-2 variants, spike mutations and immune escape. Nat Rev Microbiol *19*, 409-424.

Heo, K., and Lee, S. (2020). TSPAN8 as a novel emerging therapeutic target in cancer for monoclonal antibody therapy. Biomolecules *10*, 388.

Hoffmann, M., Kleine-Weber, H., and Pöhlmann, S. (2020a). A multibasic cleavage site in the spike protein of SARS-CoV-2 is essential for infection of human lung cells. Molecular Cell.

Hoffmann, M., Kleine-Weber, H., Schroeder, S., Kruger, N., Herrler, T., Erichsen, S., Schiergens, T.S., Herrler, G., Wu, N.H., Nitsche, A., *et al.* (2020b). SARS-CoV-2 Cell Entry Depends on ACE2 and TMPRSS2 and Is Blocked by a Clinically Proven Protease Inhibitor. Cell *181*, 271-280 e278.

https://coronavirus.jhu.edu/map.html) (2022). COVID-19 Cases. John Hopkins University and Medicine.

Iketani, S., Liu, L., Guo, Y., Liu, L., Chan, J.F., Huang, Y., Wang, M., Luo, Y., Yu, J., Chu, H., *et al.* (2022). Antibody evasion properties of SARS-CoV-2 Omicron sublineages. Nature.

Kaneko, Y., Kuwano, K., Kunitake, R., Kawasaki, M., Hagimoto, N., and Hara, N. (2000). B7-1, B7-2 and class II MHC molecules in idiopathic pulmonary fibrosis and bronchiolitis obliterans-organizing pneumonia. European Respiratory Journal *15*, 49-55.

Kassambara, A., and Mundt, F. (2017). Package 'factoextra'. Extract and visualize the results of multivariate data analyses *76*.

Kim, I.S., Jenni, S., Stanifer, M.L., Roth, E., Whelan, S.P., van Oijen, A.M., and Harrison, S.C. (2017). Mechanism of membrane fusion induced by vesicular stomatitis virus G protein. Proc Natl Acad Sci U S A *114*, E28-E36.

Kim, T.-K., Park, C.S., Jeoung, M.H., Lee, W.R., Go, N.K., Choi, J.R., Lee, T.S., Shim, H., and Lee, S. (2015). Generation of a human antibody that inhibits TSPAN8-mediated invasion of metastatic colorectal cancer cells. Biochemical and Biophysical Research Communications *468*, 774-780.

Kummer, D., Steinbacher, T., Schwietzer, M.F., Thölmann, S., and Ebnet, K. (2020). Tetraspanins: integrating cell surface receptors to functional microdomains in homeostasis and disease. Medical Microbiology and Immunology *209*, 397-405.

Lambrecht, B.N., and Hammad, H. (2010). The role of dendritic and epithelial cells as master regulators of allergic airway inflammation. The Lancet *376*, 835-843.
Lamers, M.M., Beumer, J., van der Vaart, J., Knoops, K., Puschhof, J., Breugem, T.I., Ravelli, R.B., van Schayck, J.P., Mykytyn, A.Z., and Duimel, H.Q. (2020). SARS-CoV-2 productively infects human gut enterocytes. Science.
Lê, S., Josse, J., and Mazet, F. (2008). Package 'FactoMineR. J Stat Softw [Internet] *25*, 1-18.
Letuve, S., Sallon, C., Yang, X., Dumay, A., Bedja, S., Hamidi, F., Guillou, N., Mordant, P., Pretolani, M., and Taillé, C. (2019). Role of DNA methylation in Muc5AC hyperexpression in severe asthma (Eur Respiratory Soc).
Li, F., Li, W., Farzan, M., and Harrison, S.C. (2005). Structure of SARS coronavirus spike receptor-binding domain complexed with receptor. Science *309*, 1864-1868.
Lopez-Giraldo, A., Cruz, T., Molins, L., Guirao, A., Saco, A., Cuerpo, S., Ramirez, J., Agusti, A., and Faner, R. (2018). Characterization, localization and comparison of c-Kit+ lung cells in never smokers and smokers with and without COPD. BMC Pulm Med *18*, 123.
Martins, S.d.T., and Alves, L.R. (2020). Extracellular vesicles in viral infections: two sides of the same coin? Frontiers in Cellular and Infection Microbiology, 737.
Mason, R.J. (2020). Pathogenesis of COVID-19 from a cell biology perspective. Eur Respir J *55*.
Meng, B., Abdullahi, A., Ferreira, I., Goonawardane, N., Saito, A., Kimura, I., Yamasoba, D., Gerber, P.P., Fatihi, S., Rathore, S*., et al.* (2022). Altered TMPRSS2 usage by SARS-CoV-2 Omicron impacts infectivity and fusogenicity. Nature.
Merad, M., and Martin, J.C. (2020). Pathological inflammation in patients with COVID-19: a key role for monocytes and macrophages. Nat Rev Immunol *20*, 355-362.
Mick, E., Kamm, J., Pisco, A.O., Ratnasiri, K., Babik, J.M., Castañeda, G., DeRisi, J.L., Detweiler, A.M., Hao, S.L., and Kangelaris, K.N. (2020). Upper airway gene expression reveals suppressed immune responses to SARS-CoV-2 compared with other respiratory viruses. Nature communications *11*, 1-7.
Nawijn, M.C., and Timens, W. (2020). Can ACE 2 expression explain SARS-CoV-2 infection of the respiratory epithelia in COVID-19? Molecular Systems Biology *16*, e9841.
Nazarenko, I., Rana, S., Baumann, A., McAlear, J., Hellwig, A., Trendelenburg, M., Lochnit, G., Preissner, K.T., and Zöller, M. (2010). Cell surface tetraspanin Tspan8

contributes to molecular pathways of exosome-induced endothelial cell activation. Cancer research *70*, 1668-1678.

Pastorino, R., Pezzullo, A.M., Villani, L., Causio, F.A., Axfors, C., Contopoulos-Ioannidis, D.G., Boccia, S., and Ioannidis, J.P. (2022). Change in age distribution of COVID-19 deaths with the introduction of COVID-19 vaccination. Environmental research *204*, 112342.

Planas, D., Saunders, N., Maes, P., Guivel-Benhassine, F., Planchais, C., Buchrieser, J., Bolland, W.-H., Porrot, F., Staropoli, I., and Lemoine, F. (2022). Considerable escape of SARS-CoV-2 Omicron to antibody neutralization. Nature *602*, 671-675.

Planas, D., Veyer, D., Baidaliuk, A., Staropoli, I., Guivel-Benhassine, F., Rajah, M.M., Planchais, C., Porrot, F., Robillard, N., Puech, J.*, et al.* (2021). Reduced sensitivity of SARS-CoV-2 variant Delta to antibody neutralization. Nature *596*, 276-280.

Ravindra, N.G., Alfajaro, M.M., Gasque, V., Wei, J., Filler, R.B., Huston, N.C., Wan, H., Szigeti-Buck, K., Wang, B., Montgomery, R.R.*, et al.* (2020). Single-cell longitudinal analysis of SARS-CoV-2 infection in human bronchial epithelial cells. bioRxiv.

Robinot, R., Hubert, M., de Melo, G.D., Lazarini, F., Bruel, T., Smith, N., Levallois, S., Larrous, F., Fernandes, J., and Gellenoncourt, S. (2021). SARS-CoV-2 infection induces the dedifferentiation of multiciliated cells and impairs mucociliary clearance. Nature communications *12*, 1-16.

Ross, J.T., Nesseler, N., Lee, J.W., Ware, L.B., and Matthay, M.A. (2019). The ex vivo human lung: research value for translational science. JCI Insight *4*.

Sachs, N., Papaspyropoulos, A., Zomer-van Ommen, D.D., Heo, I., Bottinger, L., Klay, D., Weeber, F., Huelsz-Prince, G., Iakobachvili, N., Amatngalim, G.D.*, et al.* (2019). Long-term expanding human airway organoids for disease modeling. EMBO J *38*.

Salahudeen, A.A., Choi, S.S., Rustagi, A., Zhu, J., van Unen, V., de la O, S.M., Flynn, R.A., Margalef-Català, M., Santos, A.J., and Ju, J. (2020). Progenitor identification and SARS-CoV-2 infection in human distal lung organoids. Nature *588*, 670-675.

Sarma, A., Christenson, S.A., Byrne, A., Mick, E., Pisco, A.O., DeVoe, C., Deiss, T., Ghale, R., Zha, B.S., Tsitsiklis, A.*, et al.* (2021). Tracheal aspirate RNA sequencing identifies distinct immunological features of COVID-19 ARDS. Nat Commun *12*, 5152.

Schleimer, R.P., Kato, A., Kern, R., Kuperman, D., and Avila, P.C. (2007). Epithelium: at the interface of innate and adaptive immune responses. J Allergy Clin Immunol *120*, 1279-1284.

Schultze, J.L., and Aschenbrenner, A.C. (2021). COVID-19 and the human innate immune system. Cell *184*, 1671-1692.

Sette, A., and Crotty, S. (2021). Adaptive immunity to SARS-CoV-2 and COVID-19. Cell *184*, 861-880.

Shafiee, A., Moradi, L., Lim, M., and Brown, J. (2021). Coronavirus disease 2019: A tissue engineering and regenerative medicine perspective. Stem Cells Transl Med *10*, 27-38.

Shang, J., Wan, Y., Luo, C., Ye, G., Geng, Q., Auerbach, A., and Li, F. (2020). Cell entry mechanisms of SARS-CoV-2. Proc Natl Acad Sci U S A *117*, 11727-11734.

Shilts, J., Crozier, T.W., Greenwood, E.J., Lehner, P.J., and Wright, G.J. (2021). No evidence for basigin/CD147 as a direct SARS-CoV-2 spike binding receptor. Scientific reports *11*, 1-10.

Sigal, A. (2022). Milder disease with Omicron: is it the virus or the pre-existing immunity? Nature Reviews Immunology, 1-3.

Simon-Loriere, E., and Schwartz, O. (2022). Towards SARS-CoV-2 serotypes? Nat Rev Microbiol *20*, 187-188.

Simoneau, C.R., and Ott, M. (2020). Modeling Multi-organ Infection by SARS-CoV-2 Using Stem Cell Technology. Cell Stem Cell *27*, 859-868.

Skevaki, C., Karsonova, A., Karaulov, A., Fomina, D., Xie, M., Chinthrajah, S., Nadeau, K.C., and Renz, H. (2021). SARS-CoV-2 infection and COVID-19 in asthmatics: a complex relationship. Nature Reviews Immunology *21*, 202-203.

Smith, J.C., and Sheltzer, J.M. (2020).

Suryawanshi, R.K., Chen, I.P., Ma, T., Syed, A.M., Brazer, N., Saldhi, P., Simoneau, C.R., Ciling, A., Khalid, M.M., Sreekumar, B.*, et al.* (2022). Limited Cross-Variant Immunity after Infection with the SARS-CoV-2 Omicron Variant Without Vaccination. medRxiv.

Tarke, A., Coelho, C.H., Zhang, Z., Dan, J.M., Yu, E.D., Methot, N., Bloom, N.I., Goodwin, B., Phillips, E., Mallal, S.*, et al.* (2022). SARS-CoV-2 vaccination induces immunological T cell memory able to cross-recognize variants from Alpha to Omicron. Cell *185*, 847-859 e811.

Team, R.C. (2013). R: A language and environment for statistical computing.

Tegally, H., Wilkinson, E., Giovanetti, M., Iranzadeh, A., Fonseca, V., Giandhari, J., Doolabh, D., Pillay, S., San, E.J., and Msomi, N. (2021). Detection of a SARS-CoV-2 variant of concern in South Africa. Nature *592*, 438-443.

Thorne, L.G., Bouhaddou, M., Reuschl, A.-K., Zuliani-Alvarez, L., Polacco, B., Pelin, A., Batra, J., Whelan, M.V., Hosmillo, M., and Fossati, A. (2022). Evolution of enhanced innate immune evasion by SARS-CoV-2. Nature *602*, 487-495.

Travaglini, K.J., Nabhan, A.N., Penland, L., Sinha, R., Gillich, A., Sit, R.V., Chang, S., Conley, S.D., Mori, Y., and Seita, J. (2020). A molecular cell atlas of the human lung from single-cell RNA sequencing. Nature *587*, 619-625.

VanBlargan, L.A., Errico, J.M., Halfmann, P.J., Zost, S.J., Crowe, J.E., Purcell, L.A., Kawaoka, Y., Corti, D., Fremont, D.H., and Diamond, M.S. (2022). An infectious SARS-CoV-2 B. 1.1. 529 Omicron virus escapes neutralization by therapeutic monoclonal antibodies. Nature medicine, 1-6.

Vieira Braga, F.A., Kar, G., Berg, M., Carpaij, O.A., Polanski, K., Simon, L.M., Brouwer, S., Gomes, T., Hesse, L., and Jiang, J. (2019). A cellular census of human lungs identifies novel cell states in health and in asthma. Nature medicine *25*, 1153-1163.

Voglstaetter, M., Thomsen, A.R., Nouvel, J., Koch, A., Jank, P., Navarro, E.G., Gainey-Schleicher, T., Khanduri, R., Groß, A., and Rossner, F. (2019). Tspan8 is expressed in breast cancer and regulates E-cadherin/catenin signalling and metastasis accompanied by increased circulating extracellular vesicles. The Journal of pathology *248*, 421-437.

Volz, E., Mishra, S., Chand, M., Barrett, J.C., Johnson, R., Geidelberg, L., Hinsley, W.R., Laydon, D.J., Dabrera, G., and O'Toole, Á. (2021). Assessing transmissibility of SARS-CoV-2 lineage B. 1.1. 7 in England. Nature *593*, 266-269.

Wagar, L.E., Salahudeen, A., Constantz, C.M., Wendel, B.S., Lyons, M.M., Mallajosyula, V., Jatt, L.P., Adamska, J.Z., Blum, L.K., Gupta, N.*, et al.* (2021). Modeling human adaptive immune responses with tonsil organoids. Nat Med *27*, 125-135.

Walls, A.C., Park, Y.J., Tortorici, M.A., Wall, A., McGuire, A.T., and Veesler, D. (2020). Structure, Function, and Antigenicity of the SARS-CoV-2 Spike Glycoprotein. Cell *181*, 281-292 e286.

Walls, A.C., Tortorici, M.A., Bosch, B.-J., Frenz, B., Rottier, P.J., DiMaio, F., Rey, F.A., and Veesler, D. (2016). Cryo-electron microscopy structure of a coronavirus spike glycoprotein trimer. Nature *531*, 114-117.

Wang, K., Chen, W., Zhou, Y.-S., Lian, J.-Q., Zhang, Z., Du, P., Gong, L., Zhang, Y., Cui, H.-Y., and Geng, J.-J. (2020). SARS-CoV-2 invades host cells via a novel route: CD147-spike protein. BioRxiv.

Wang, R., Simoneau, C.R., Kulsuptrakul, J., Bouhaddou, M., Travisano, K.A., Hayashi, J.M., Carlson-Stevermer, J., Zengel, J.R., Richards, C.M., and Fozouni, P. (2021a). Genetic screens identify host factors for SARS-CoV-2 and common cold coronaviruses. Cell *184*, 106-119. e114.

Wang, S., Qiu, Z., Hou, Y., Deng, X., Xu, W., Zheng, T., Wu, P., Xie, S., Bian, W., Zhang, C*., et al.* (2021b). AXL is a candidate receptor for SARS-CoV-2 that promotes infection of pulmonary and bronchial epithelial cells. Cell Res.

Washington, N.L., Gangavarapu, K., Zeller, M., Bolze, A., Cirulli, E.T., Barrett, K.M.S., Larsen, B.B., Anderson, C., White, S., and Cassens, T. (2021). Emergence and rapid transmission of SARS-CoV-2 B. 1.1. 7 in the United States. Cell *184*, 2587-2594. e2587.

Wickham, H., Chang, W., Henry, L., Pedersen, T., Takahashi, K., Wilke, C., Woo, K., Yutani, H., and Dunnington, D. (2016). Springer-Verlag. New York.

Wickham, H., Francois, R., Henry, L., and Müller, K. others. 2015.“. Dplyr: A Grammar of Data Manipulation” R Package Version 04 *3*.

Xian, W., and McKeon, F. (2012). Adult stem cells underlying lung regeneration. Cell Cycle *11*, 887-894.

Yan, R., Zhang, Y., Li, Y., Xia, L., Guo, Y., and Zhou, Q. (2020). Structural basis for the recognition of SARS-CoV-2 by full-length human ACE2. Science *367*, 1444-1448.

Youk, J., Kim, T., Evans, K.V., Jeong, Y.I., Hur, Y., Hong, S.P., Kim, J.H., Yi, K., Kim, S.Y., Na, K.J*., et al.* (2020). Three-Dimensional Human Alveolar Stem Cell Culture Models Reveal Infection Response to SARS-CoV-2. Cell Stem Cell *27*, 905-919 e910.

Zhou, Y., Zhou, B., Pache, L., Chang, M., Khodabakhshi, A.H., Tanaseichuk, O., Benner, C., and Chanda, S.K. (2019). Metascape provides a biologist-oriented resource for the analysis of systems-level datasets. Nature communications *10*, 1-10.

Ziegler, C.G.K., Allon, S.J., Nyquist, S.K., Mbano, I.M., Miao, V.N., Tzouanas, C.N., Cao, Y., Yousif, A.S., Bals, J., Hauser, B.M*., et al.* (2020). SARS-CoV-2 Receptor ACE2 Is an Interferon-Stimulated Gene in Human Airway Epithelial Cells and Is Detected in Specific Cell Subsets across Tissues. Cell *181*, 1016-1035 e1019.

Zissel, G., Ernst, M., Rabe, K., Papadopoulos, T., Magnussen, H., Schlaak, M., and Müller-Quernheim, J. (2000). Human alveolar epithelial cells type II are capable of

regulating T-cell activity. Journal of investigative medicine: the official publication of the American Federation for Clinical Research *48*, 66-75.

A

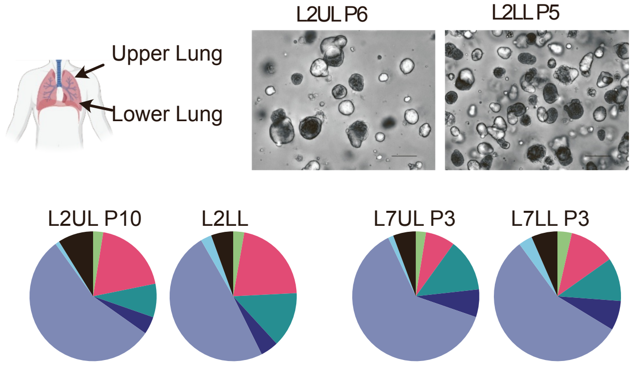

B

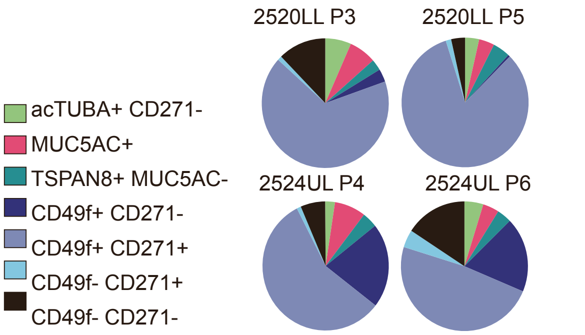

C

Sample ID	Tissue Type	P 1-5	P 6-10	P 11-16	P 17-20
2425	Normal Lung	11	15		
2477	Normal Lung	28	11		5
2478	Normal Lung	14	10	3	
2520	Normal Lung	14			
2521	Normal Lung	20	11	4	5
2522	Normal Lung	15	6	2	2
2523	Normal Lung	12	6	8	
2524	Normal Lung	16	9	9	
2525	Normal Lung	21		8	
2526	Normal Lung	16	2		
2527	Normal Lung	27			
2531	Normal Lung	4	21		
2547	Normal Lung	33			
2551	Normal Lung	16			
2553	Normal Lung	22			
2559	Normal Lung	11			
2561	Normal Lung	16	4		
L3	Normal Lung	5			
L5	Normal Lung	12			
L7	Normal Lung	5			

* Number of organoid cryovials in Organoid D2B Biobank (P = Passage).

D

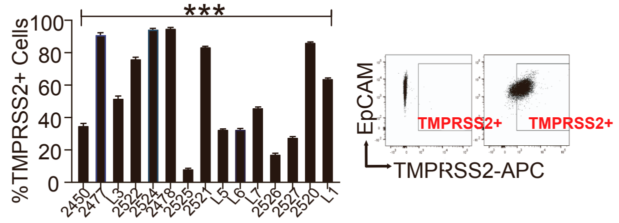

E

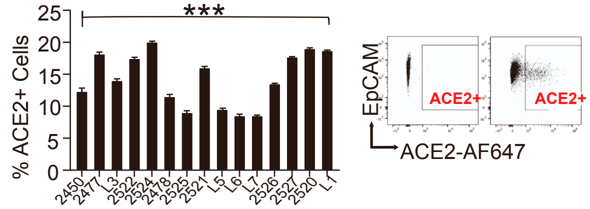

F

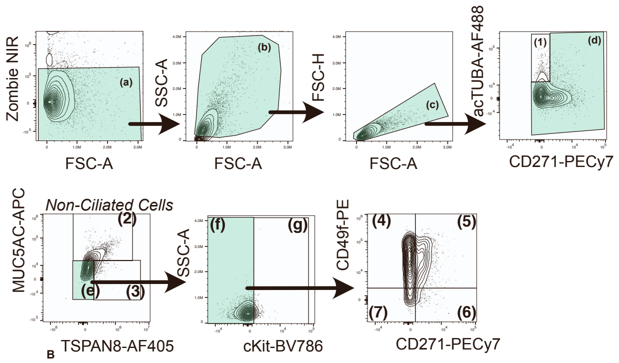

G

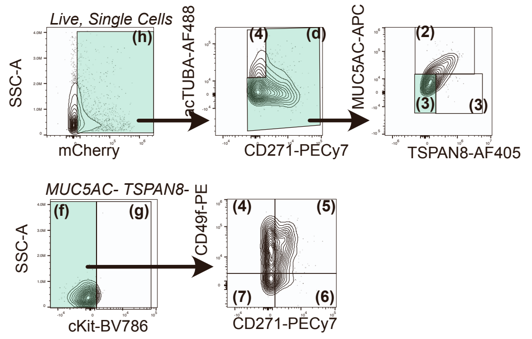

(a) Live Cells
(b) Cells
(c) Single Cells
(1) Ciliated Cells
(d) Non-Ciliated Cells
(2) Goblet-like cells
(e) MUC5AC- TSPAN8-
(3) Pre-Goblet-like cells
(f) cKit-
(g) cKit+
(4) CD49f+ CD271-
(5) CD49f+ CD271+
(6) CD49f- CD271-
(7) CD49f- CD271+
(h) mCherry+ Cells

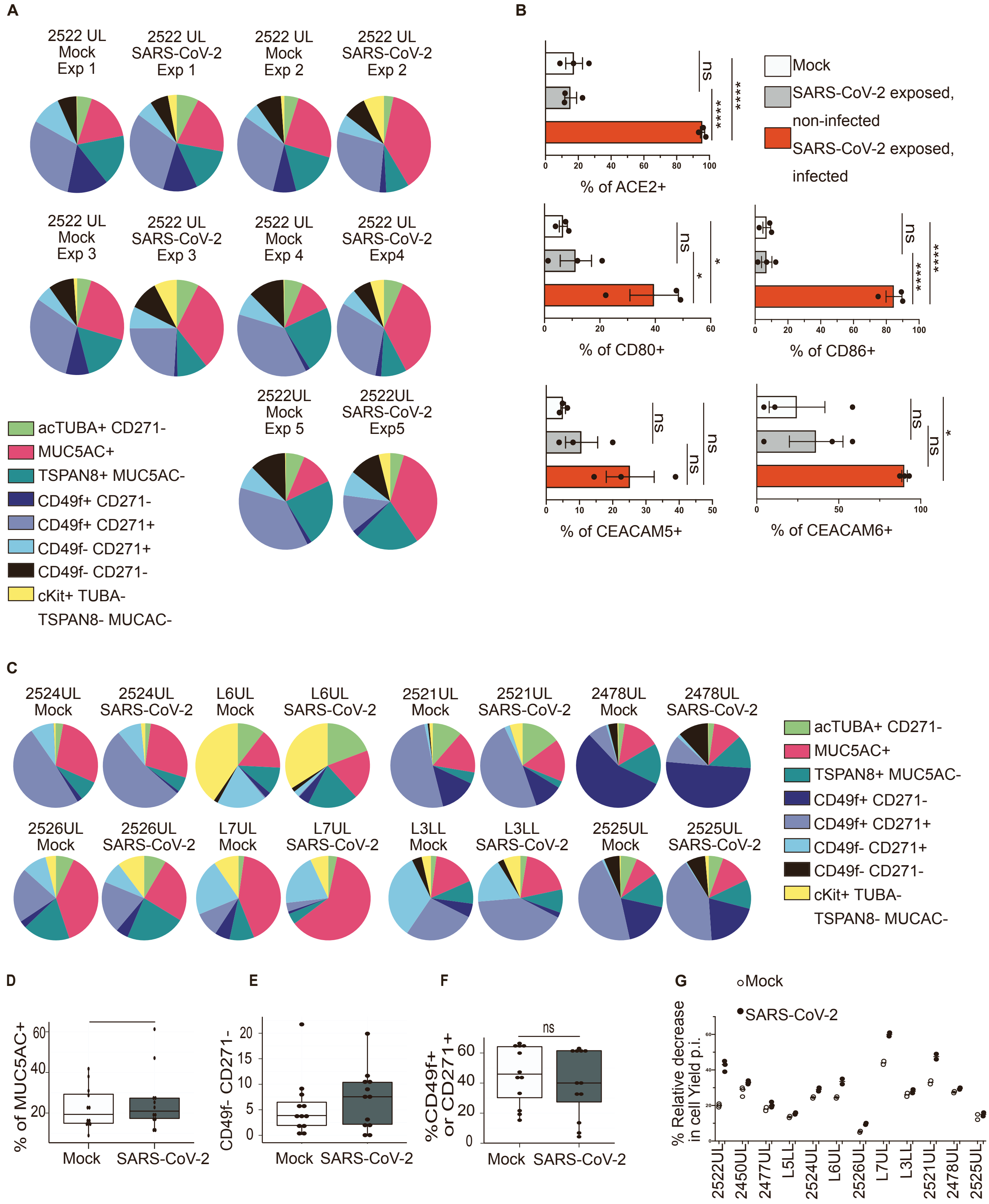
A
2522 UL Mock Exp 1
2522 UL SARS-CoV-2 Exp 1
2522 UL Mock Exp 2
2522 UL SARS-CoV-2 Exp 2
2522 UL Mock Exp 3
2522 UL SARS-CoV-2 Exp 3
2522 UL Mock Exp 4
2522 UL SARS-CoV-2 Exp4
2522UL Mock Exp 5
2522UL SARS-CoV-2 Exp5
acTUBA+ CD271-
MUC5AC+
TSPAN8+ MUC5AC-
CD49f+ CD271-
CD49f+ CD271+
CD49f- CD271+
CD49f- CD271-
cKit+ TUBA- TSPAN8- MUCAC-
B
Mock
SARS-CoV-2 exposed, non-infected
SARS-CoV-2 exposed, infected
% of ACE2+
% of CD80+
% of CD86+
% of CEACAM5+
% of CEACAM6+
ns
****
*
C
2524UL Mock
2524UL SARS-CoV-2
L6UL Mock
L6UL SARS-CoV-2
2521UL Mock
2521UL SARS-CoV-2
2478UL Mock
2478UL SARS-CoV-2
2526UL Mock
2526UL SARS-CoV-2
L7UL Mock
L7UL SARS-CoV-2
L3LL Mock
L3LL SARS-CoV-2
2525UL Mock
2525UL SARS-CoV-2
D
% of MUC5AC+
Mock
SARS-CoV-2
E
CD49f- CD271-
Mock
SARS-CoV-2
F
%CD49f+ or CD271+
ns
Mock
SARS-CoV-2
G
Mock
SARS-CoV-2
% Relative decrease in cell Yield p.i.
2522UL
2450UL
2477UL
L5LL
2524UL
L6UL
2526UL
L7UL
L3LL
2521UL
2478UL
2525UL

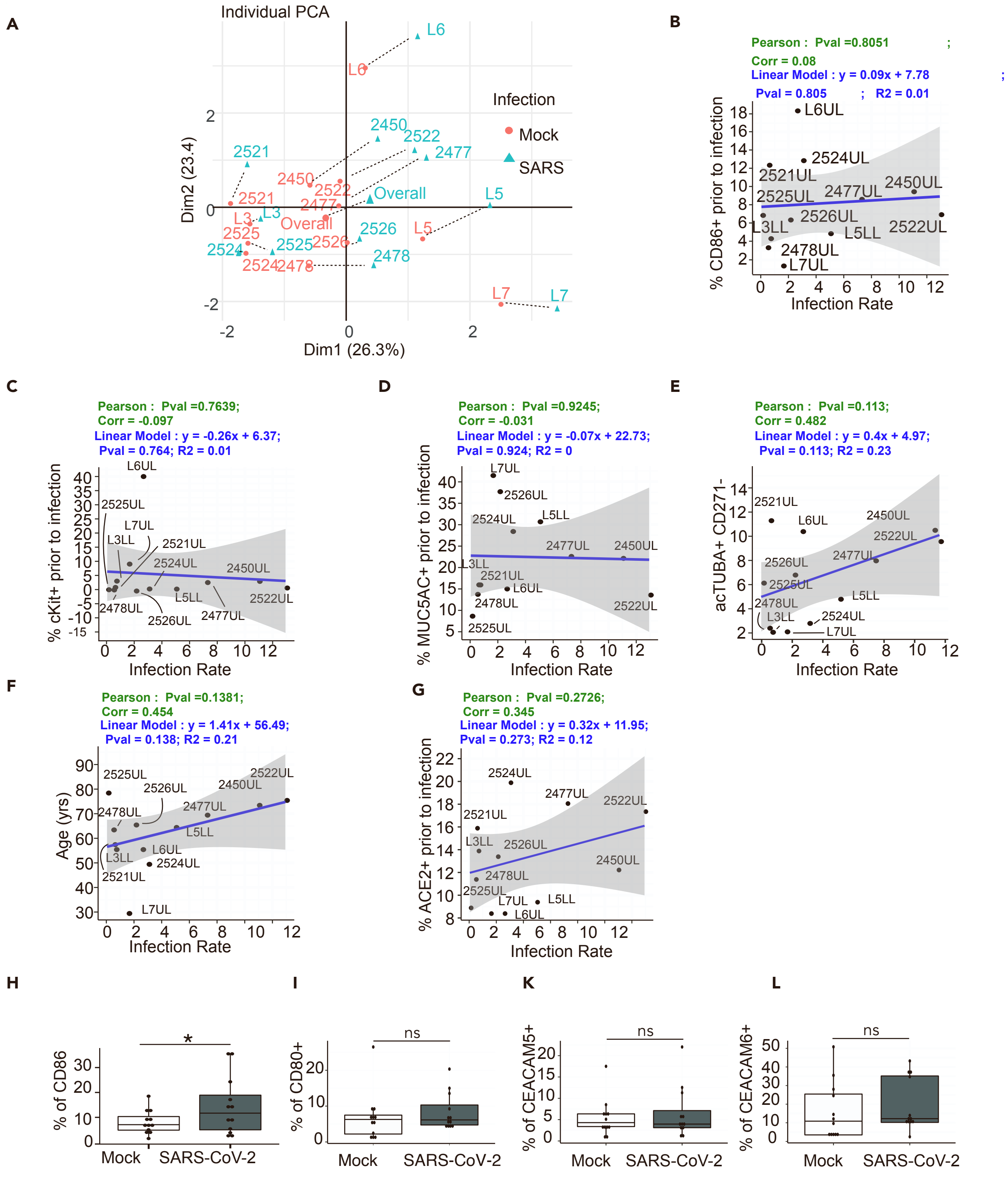
A
Individual PCA
Infection
Mock
SARS
Dim1 (26.3%)
Dim2 (23.4)
L6
L7
L5
L3
2521
2450
2522
2477
Overall
2526
2525
2524
2478
B
Pearson : Pval =0.8051 ;
Corr = 0.08
Linear Model : y = 0.09x + 7.78 ;
Pval = 0.805 ; R2 = 0.01
% CD86+ prior to infection
Infection Rate
L6UL
2524UL
2521UL
2525UL
2477UL
2450UL
2526UL
L3LL
L5LL
2522UL
2478UL
L7UL
C
Pearson : Pval =0.7639;
Corr = -0.097
Linear Model : y = -0.26x + 6.37;
Pval = 0.764; R2 = 0.01
% cKit+ prior to infection
Infection Rate
L6UL
2525UL
L7UL
L3LL
2521UL
2524UL
2450UL
L5LL
2522UL
2478UL
2526UL
2477UL
D
Pearson : Pval =0.9245;
Corr = -0.031
Linear Model : y = -0.07x + 22.73;
Pval = 0.924; R2 = 0
% MUC5AC+ prior to infection
Infection Rate
L7UL
2526UL
2524UL
L5LL
2477UL
2450UL
L3LL
2521UL
L6UL
2478UL
2522UL
2525UL
E
Pearson : Pval =0.113;
Corr = 0.482
Linear Model : y = 0.4x + 4.97;
Pval = 0.113; R2 = 0.23
acTUBA+ CD271-
Infection Rate
2521UL
L6UL
2450UL
2522UL
2477UL
2526UL
2525UL
L5LL
2478UL
L3LL
2524UL
L7UL
F
Pearson : Pval =0.1381;
Corr = 0.454
Linear Model : y = 1.41x + 56.49;
Pval = 0.138; R2 = 0.21
Age (yrs)
Infection Rate
2525UL
2526UL
2450UL
2522UL
2478UL
2477UL
L5LL
L6UL
L3LL
2524UL
2521UL
L7UL
G
Pearson : Pval =0.2726;
Corr = 0.345
Linear Model : y = 0.32x + 11.95;
Pval = 0.273; R2 = 0.12
% ACE2+ prior to infection
Infection Rate
2524UL
2477UL
2522UL
2521UL
L3LL
2526UL
2450UL
2478UL
2525UL
L7UL
L5LL
L6UL
H
% of CD86
*
Mock
SARS-CoV-2
I
% of CD80+
ns
Mock
SARS-CoV-2
K
% of CEACAM5+
ns
Mock
SARS-CoV-2
L
% of CEACAM6+
ns
Mock
SARS-CoV-2

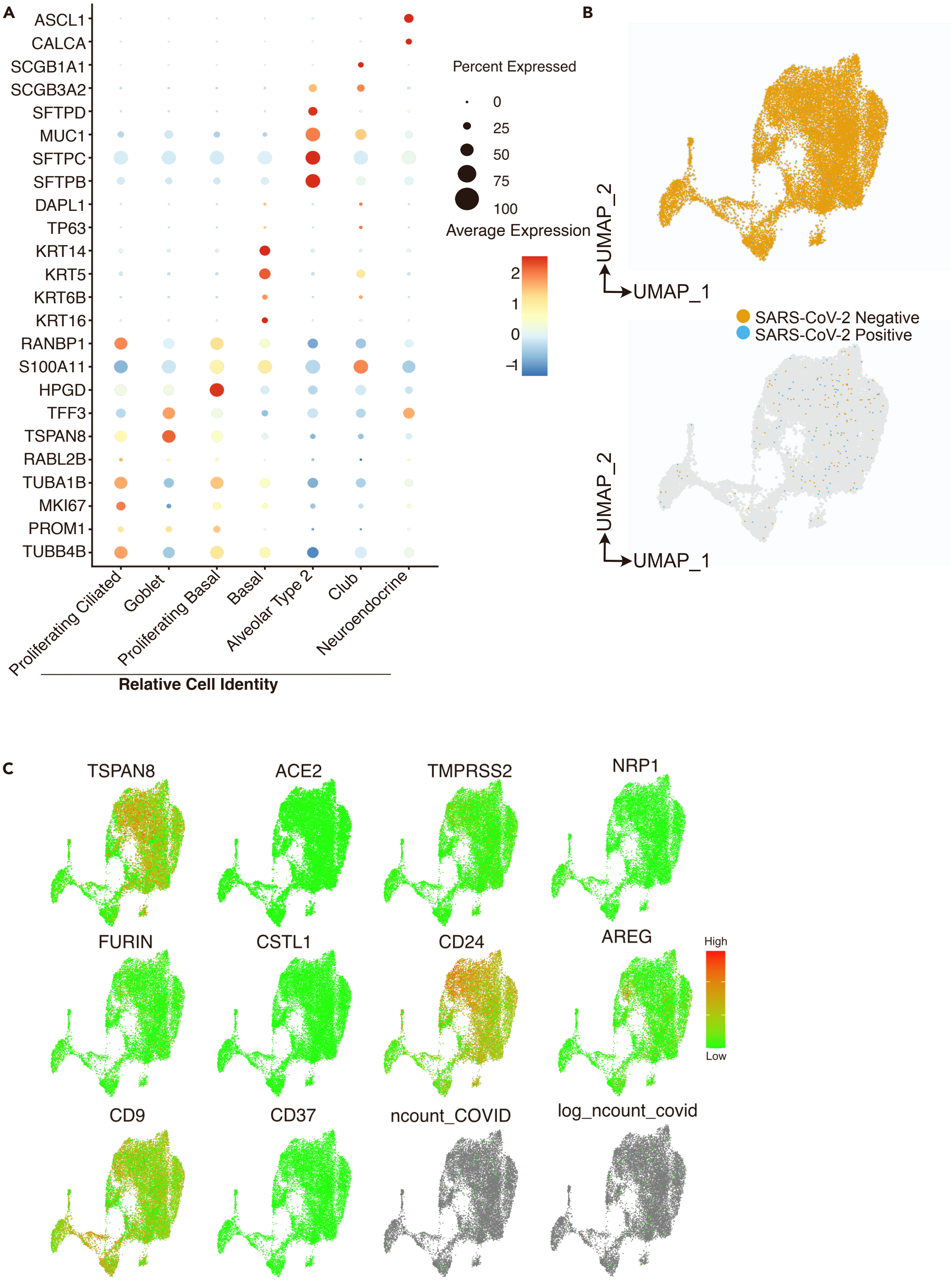
A
ASCL1
CALCA
SCGB1A1
SCGB3A2
SFTPD
MUC1
SFTPC
SFTPB
DAPL1
TP63
KRT14
KRT5
KRT6B
KRT16
RANBP1
S100A11
HPGD
TFF3
TSPAN8
RABL2B
TUBA1B
MKI67
PROM1
TUBB4B
Proliferating Ciliated
Goblet
Proliferating Basal
Basal
Alveolar Type 2
Club
Neuroendocrine
Relative Cell Identity
Percent Expressed
0
25
50
75
100
Average Expression
2
1
0
-1
B
UMAP_2
UMAP_1
SARS-CoV-2 Negative
SARS-CoV-2 Positive
UMAP_2
UMAP_1
C
TSPAN8
ACE2
TMPRSS2
NRP1
FURIN
CSTL1
CD24
AREG
High
Low
CD9
CD37
ncount_COVID
log_ncount_covid

A

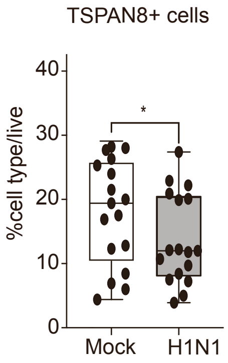

TSPAN8+ cells
%cell type/live
40
30
20
10
0
*
Mock
H1N1

B

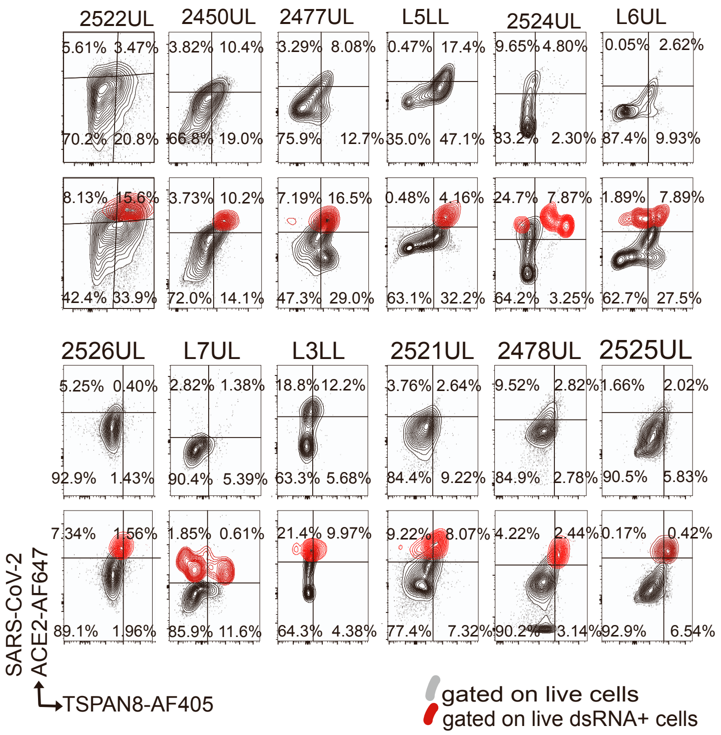

2522UL
2450UL
2477UL
L5LL
2524UL
L6UL
2526UL
L7UL
L3LL
2521UL
2478UL
2525UL
SARS-CoV-2
ACE2-AF647
TSPAN8-AF405
gated on live cells
gated on live dsRNA+ cells

C

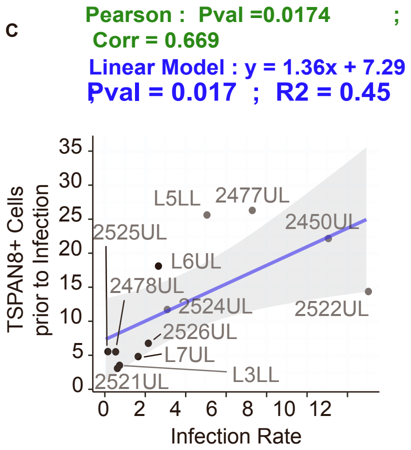

Pearson : Pval =0.0174 ;
Corr = 0.669
Linear Model : y = 1.36x + 7.29
Pval = 0.017 ; R2 = 0.45
TSPAN8+ Cells
prior to Infection
Infection Rate
L5LL
2477UL
2450UL
2525UL
L6UL
2478UL
2524UL
2522UL
2526UL
L7UL
L3LL
2521UL

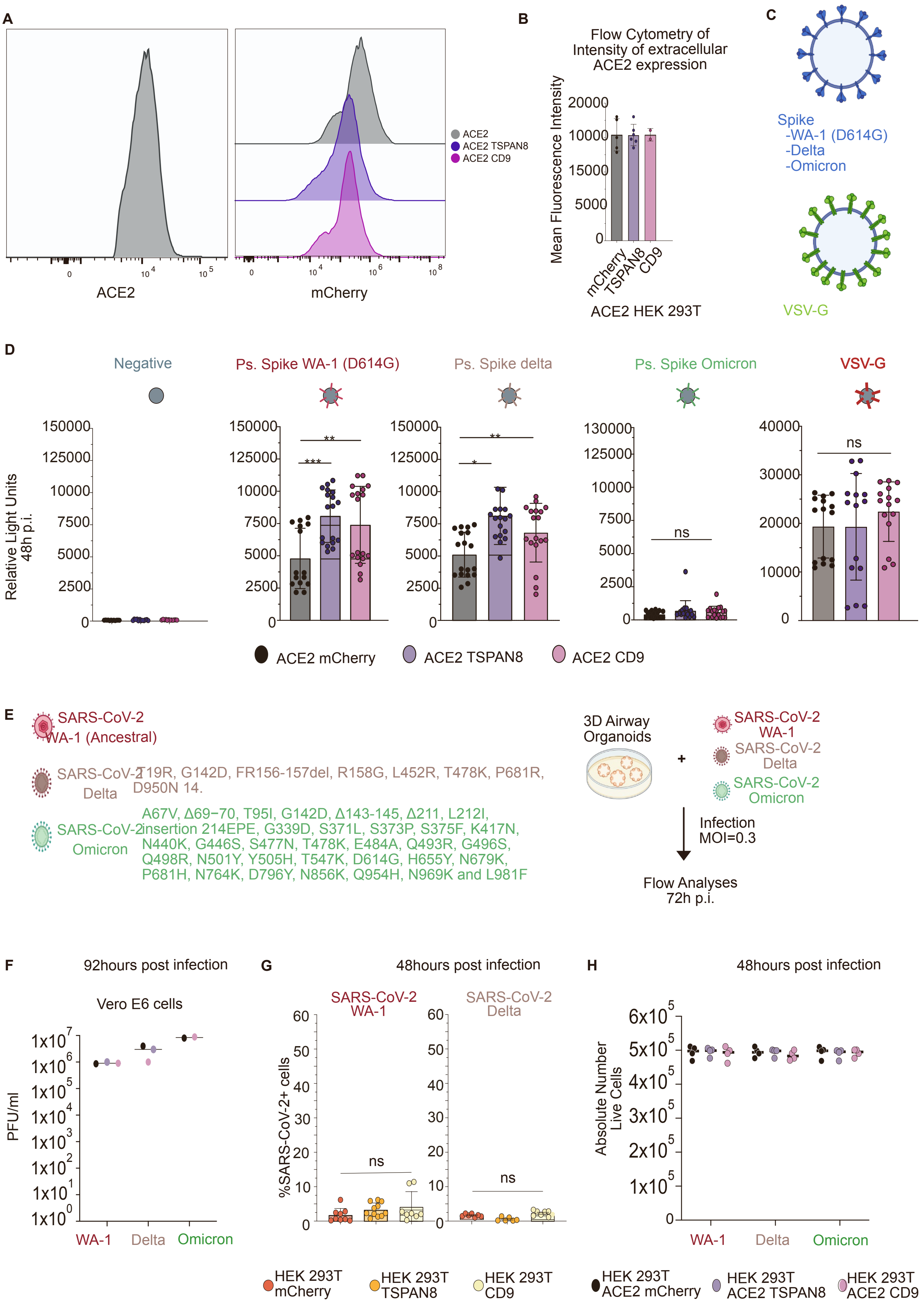

A
ACE2
mCherry
ACE2
ACE2 TSPAN8
ACE2 CD9
B
Flow Cytometry of Intensity of extracellular ACE2 expression
Mean Fluorescence Intensity
20000
10000
15000
5000
0
mCherry
TSPAN8
CD9
ACE2 HEK 293T
C
Spike
-WA-1 (D614G)
-Delta
-Omicron
VSV-G
D
Negative
Ps. Spike WA-1 (D614G)
Ps. Spike delta
Ps. Spike Omicron
VSV-G
Relative Light Units 48h p.i.
ns
ACE2 mCherry
ACE2 TSPAN8
ACE2 CD9
E
SARS-CoV-2 WA-1 (Ancestral)
SARS-CoV-2 Delta
T19R, G142D, FR156-157del, R158G, L452R, T478K, P681R, D950N 14.
SARS-CoV-2 Omicron
A67V, Δ69−70, T95I, G142D, Δ143-145, Δ211, L212I, insertion 214EPE, G339D, S371L, S373P, S375F, K417N, N440K, G446S, S477N, T478K, E484A, Q493R, G496S, Q498R, N501Y, Y505H, T547K, D614G, H655Y, N679K, P681H, N764K, D796Y, N856K, Q954H, N969K and L981F
3D Airway Organoids
SARS-CoV-2 WA-1
SARS-CoV-2 Delta
SARS-CoV-2 Omicron
Infection MOI=0.3
Flow Analyses 72h p.i.
F
92hours post infection
Vero E6 cells
PFU/ml
WA-1
Delta
Omicron
G
48hours post infection
SARS-CoV-2 WA-1
SARS-CoV-2 Delta
%SARS-CoV-2+ cells
ns
HEK 293T mCherry
HEK 293T TSPAN8
HEK 293T CD9
H
48hours post infection
Absolute Number Live Cells
WA-1
Delta
Omicron
HEK 293T ACE2 mCherry
HEK 293T ACE2 TSPAN8
HEK 293T ACE2 CD9

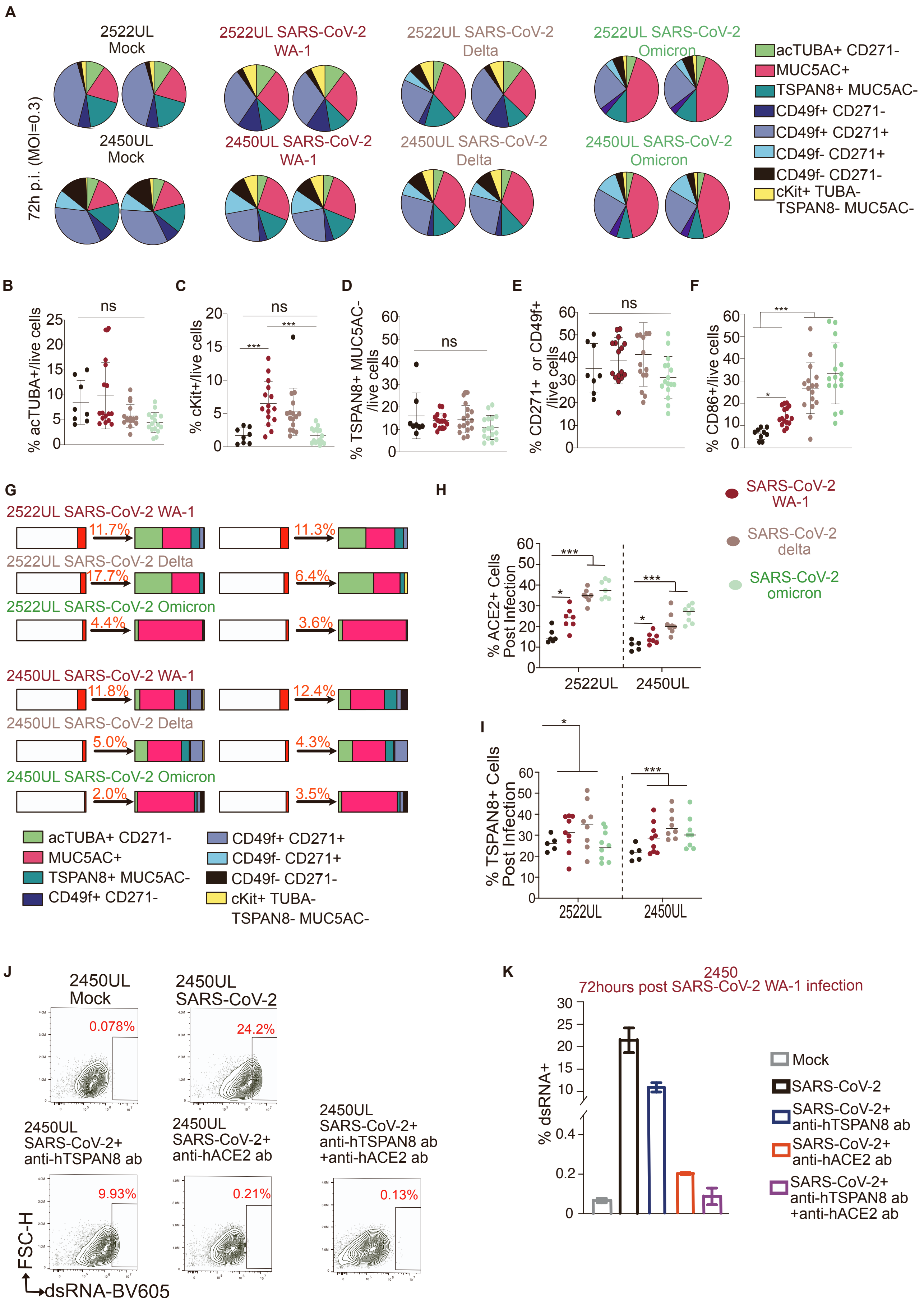
A
2522UL Mock
2522UL SARS-CoV-2 WA-1
2522UL SARS-CoV-2 Delta
2522UL SARS-CoV-2 Omicron
2450UL Mock
2450UL SARS-CoV-2 WA-1
2450UL SARS-CoV-2 Delta
2450UL SARS-CoV-2 Omicron
72h p.i. (MOI=0.3)
acTUBA+ CD271-
MUC5AC+
TSPAN8+ MUC5AC-
CD49f+ CD271-
CD49f+ CD271+
CD49f- CD271+
CD49f- CD271-
cKit+ TUBA- TSPAN8- MUC5AC-
B
% acTUBA+/live cells
ns
C
% cKit+/live cells
ns
***
***
D
% TSPAN8+ MUC5AC- /live cells
ns
E
% CD271+ or CD49f+ /live cells
ns
F
% CD86+/live cells
***
*
G
2522UL SARS-CoV-2 WA-1
11.7%
11.3%
2522UL SARS-CoV-2 Delta
17.7%
6.4%
2522UL SARS-CoV-2 Omicron
4.4%
3.6%
2450UL SARS-CoV-2 WA-1
11.8%
12.4%
2450UL SARS-CoV-2 Delta
5.0%
4.3%
2450UL SARS-CoV-2 Omicron
2.0%
3.5%
acTUBA+ CD271-
MUC5AC+
TSPAN8+ MUC5AC-
CD49f+ CD271-
CD49f+ CD271+
CD49f- CD271+
CD49f- CD271-
cKit+ TUBA- TSPAN8- MUC5AC-
H
% ACE2+ Cells Post Infection
***
*
2522UL
2450UL
SARS-CoV-2 WA-1
SARS-CoV-2 delta
SARS-CoV-2 omicron
I
% TSPAN8+ Cells Post Infection
*
***
2522UL
2450UL
J
2450UL Mock
0.078%
2450UL SARS-CoV-2
24.2%
2450UL SARS-CoV-2+ anti-hTSPAN8 ab
9.93%
2450UL SARS-CoV-2+ anti-hACE2 ab
0.21%
2450UL SARS-CoV-2+ anti-hTSPAN8 ab +anti-hACE2 ab
0.13%
FSC-H
dsRNA-BV605
K
2450
72hours post SARS-CoV-2 WA-1 infection
% dsRNA+
Mock
SARS-CoV-2
SARS-CoV-2+ anti-hTSPAN8 ab
SARS-CoV-2+ anti-hACE2 ab
SARS-CoV-2+ anti-hTSPAN8 ab +anti-hACE2 ab
