# Supplementary material for: SARS-CoV-2 infection of airway organoids reveals conserved use of Tetraspanin-8 by Ancestral, Delta, and Omicron variants
Source: Stem Cell Reports. 2023 Feb 23;18(3):636–53. doi: 10.1016/j.stemcr.2023.01.011 (PMC9948283; doi:10.1016/j.stemcr.2023.01.011)
Supplement: Document S2. Article plus supplemental information [file mmc5.pdf]

Stem Cell Reports
ISSCR
Article
OPEN ACCESS

SARS-CoV-2 infection of airway organoids reveals conserved use of Tetraspanin-8 by Ancestral, Delta, and Omicron variants

Lisiena Hysenaj,[1] Samantha Little,[1] Kayla Kulhanek,[1] Melia Magnen,[4,5] Kriti Bahl,[1] Oghenekevwe M. Gbenedio,[1] Morgan Prinz,[1] Lauren Rodriguez,[2,3,4] Christopher Andersen,[3,4] Arjun Arkal Rao,[3,4,7] Alan Shen,[3] Jean-Christophe Lone,[16] Leonard C. Lupin-Jimenez,[3,4] Luke R. Bonser,[6] Nina K. Serwas,[7] Eran Mick,[8,9,15] Mir M. Khalid,[12] Taha Y. Taha,[12] Renuka Kumar,[12] Jack Z. Li,[10] Vivianne W. Ding,[10] Shotaro Matsumoto,[11] Mazharul Maishan,[11] Bharath Sreekumar,[12] Camille Simoneau,[12] Irina Nazarenko,[19,20,21] Michael G. Tomlinson,[17,18] Khajida Khan,[22,23] Anne von Gottberg,[26,27] Alex Sigal,[22,23,24,25] Mark R. Looney,[4,5,9] Gabriela K. Fragiadakis,[3,4,13] David M. Jablons,[9,10,11] Charles R. Langelier,[8,9,12,15] Michael Matthay,[9,11] Matthew Krummel,[1,7] David J. Erle,[3,4,6,9] Alexis J. Combes,[3,4,7] Anita Sil,[2] Melanie Ott,[12,13,14] Johannes R. Kratz,[4,10] and Jeroen P. Roose[1,*]

[1]Department of Anatomy, University of California, San Francisco, 513 Parnassus Avenue, San Francisco, CA 94143, USA
[2]Department of Microbiology and Immunology, University of California, San Francisco, San Francisco, CA 94143, USA
[3]UCSF CoLabs, University of California, San Francisco, San Francisco, CA 94143, USA
[4]ImmunoX Initiative, University of California, San Francisco, San Francisco, CA, USA
[5]Department of Medicine, University of California, San Francisco, San Francisco, CA 94143, USA
[6]Lung Biology Center, Department of Medicine, University of California, San Francisco, San Francisco, CA, USA
[7]Department of Pathology, University of California, San Francisco, San Francisco, CA 94143, USA
[8]Division of Infectious Diseases, University of California, San Francisco, San Francisco, CA, USA
[9]Division of Pulmonary and Critical Care, San Francisco, San Francisco, CA, USA
[10]Department of Surgery, Division of Cardiothoracic Surgery, University of California, San Francisco, San Francisco, CA, USA
[11]Cardiovascular Research Institute, Departments of Medicine and Anesthesia, University of California, San Francisco, San Francisco, CA 94143, USA
[12]Gladstone Institute of Virology, Department of Medicine, University of California, San Francisco, San Francisco, CA, USA
[13]Department of Medicine, Division of Rheumatology, University of California, San Francisco, San Francisco, CA 94143, USA
[14]Quantitative Biosciences Institute COVID-19 Research Group, University of California, San Francisco, San Francisco, CA, USA
[15]Chan Zuckerberg Biohub, San Francisco, CA 94158, USA
[16]School of Life Science, University of Essex, C04 3SQ Colchester, UK
[17]School of Biosciences, University of Birmingham, Birmingham, UK
[18]Centre of Membrane Proteins and Receptors, Universities of Birmingham and Nottingham, Midlands, UK
[19]Institute for Infection Prevention and Hospital Epidemiology, University of Freiburg, Freiburg, Germany
[20]Faculty of Medicine, University of Freiburg, 79106 Freiburg, Germany
[21]German Cancer Consortium, Partner Site Freiburg and German Cancer Research Center, Heidelberg, Germany
[22]Africa Health Research Institute, Durban, South Africa
[23]School of Laboratory Medicine and Medical Sciences, University of KwaZulu-Natal, Durban, South Africa
[24]Max Planck Institute for Infection Biology, Berlin, Germany
[25]Centre for the AIDS Program of Research, Durban, South Africa
[26]National Institute for Communicable Diseases of the National Health Laboratory Service, Johannesburg, South Africa
[27]SAMRC Antibody Immunity Research Unit, University of the Witwatersrand, Johannesburg, South Africa
*Correspondence: jeroen.roose@ucsf.edu
https://doi.org/10.1016/j.stemcr.2023.01.011

SUMMARY

Ancestral SARS coronavirus-2 (SARS-CoV-2) and variants of concern (VOC) caused a global pandemic with a spectrum of disease severity. The mechanistic explaining variations related to airway epithelium are relatively understudied. Here, we biobanked airway organoids (AO) by preserving stem cell function. We optimized viral infection with H1N1/PR8 and comprehensively characterized epithelial responses to SARS-CoV-2 infection in phenotypically stable AO from 20 different subjects. We discovered Tetraspanin-8 (TSPAN8) as a facilitator of SARS-CoV-2 infection. TSPAN8 facilitates SARS-CoV-2 infection rates independently of ACE2-Spike interaction. In head-to-head comparisons with Ancestral SARS-CoV-2, Delta and Omicron VOC displayed lower overall infection rates of AO but triggered changes in epithelial response. All variants shared highest tropism for ciliated and goblet cells. TSPAN8-blocking antibodies diminish SARS-CoV-2 infection and may spur novel avenues for COVID-19 therapy.

INTRODUCTION

Severe Acute Respiratory Syndrome Coronavirus-2 (SARS-CoV-2) has caused a global pandemic of coronavirus disease (COVID-19) with more than half a billion cases worldwide (https://coronavirus.jhu.edu). While most SARS-CoV-2-infected individuals develop asymptomatic to mild disease, some develop a severe disease characterized by immune cell dysfunction (Bastard et al., 2021). Elegant work carefully mapped characteristics of SARS-CoV-2 responses in blood- and airway-immune cells. Vaccination programs have resulted in reduced cases of COVID-19

This is an open access article under the CC BY license (http://creativecommons.org/licenses/by/4.0/).

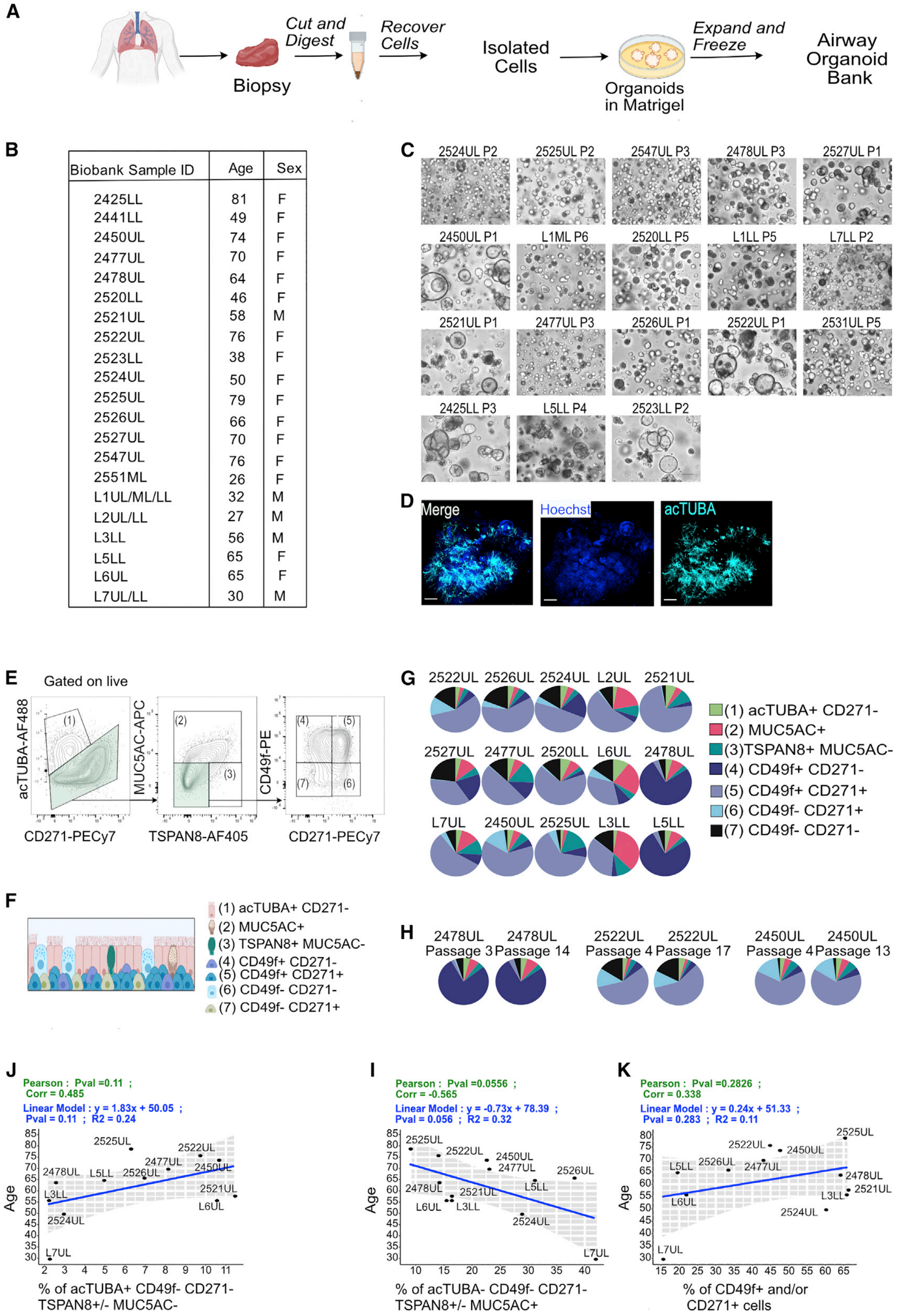

(legend on next page)

death (Pastorino et al., 2022); however, SARS-CoV-2 variants of concern (VOC) have emerged, such as Alpha (B.1.1.7), Beta (B.1.351), Delta (B.1.617.2), and Omicron (B.1.1.529) (Cobey et al., 2021; Harvey et al., 2021). Vaccinated individuals appear to retain partial T cell responses to VOC; however, Delta and Omicron escape existing neutralizing antibodies (Iketani et al., 2022; Planas et al., 2021) and caused surges in SARS-CoV-2 VOC infections (Simon-Loriere and Schwartz, 2022). The spike (Spike) glycoprotein on SARS-CoV-2 binds to human ACE2 (Yan et al., 2020), mediating membrane fusion and viral entry. Spike cleavage by host cell-type II *trans*-membrane serine proteases (TMPRSS2) results in Spike protein activation and viral entry (Hoffmann et al., 2020a). As such, ACE2 and TMPRSS2 are critical for SARS-CoV-2 entry into the cell (Wang et al., 2021); however, SARS-CoV-2 infected patients display neutralizing antibodies that bind to SARS-CoV-2 but not to Spike's ACE2-binding domain (Brouwer et al., 2020). These findings indicate that there are likely molecular interactions, in addition to the Spike/ACE2 pair, in the extracellular environment that impact SARS-CoV-2 biology in the airway epithelium.

The lung airway epithelium defends against pollutants, allergens, and pathogens and is composed of a variety of cell types. SARS-CoV-2 reportedly infects mostly ciliated cells, goblet cells, and alveolar type 2 cells, but also basal stem cells (Chua et al., 2020; Fiege et al., 2021; Han et al., 2021; Lamers et al., 2020; Mason, 2020; Ravindra et al., 2020; Robinot et al., 2021; Salahudeen et al., 2020; Shafiee et al., 2021; Youk et al., 2020). SARS-CoV-2 elicits variation in disease spectrum of COVID-19, but the underpinnings of variation related to airway epithelium are largely unknown. Many questions remain regarding lung epithelial responses to SARS-CoV-2 infection in different people, the molecules and mechanisms that enable infection, and whether these mechanisms are conserved or distinct for different SARS-CoV-2 VOC.

Here we generated and characterized a biobank of 20 stable, but unique airway organoids (AOs) derived from adult stem cells of different individuals. We used this biobank to first optimize viral infection of AO with H1N1/PR8 influenza, and next performed a comprehensive analysis of SARS-CoV-2 infection with repeat infections. Spectral flow analysis of infected AO was used to assess cellular and functional responses of the epithelial cell compartment. Single-cell RNA sequencing (scRNA-seq) and Spectral flow enabled the discovery of Tetraspanin-8 (TSPAN8) as a conserved mediator of SARS-CoV-2 Ancestral (WA-1)-, Delta-, and Omicron-variant infection. Reductionist HEK293T cell-pseudovirus approaches showed that TSPAN8 facilitates viral entry independently of the Spike-ACE2 interaction. We show that TSPAN8 is not an alternative entry receptor. Blocking TSPAN8 in airway epithelial organoids prior to infection is associated with a decrease in the viral load of AOs. Based on our TSPAN8 work in the context of cancer (Nazarenko et al., 2010; Voglstaetter et al., 2019), we propose that TSPAN8 as a potential therapeutic target for controlling the severity of COVID-19 disease.

RESULTS

Generation of a comprehensive and stable 3D airway organoid biobank

To perform a comprehensive analysis of SARS-CoV-2 infection of complex airway epithelial cell subsets in different individuals, we first generated an AO biobank from biopsies (Figure 1A and Table S1). 3D AOs from 21 subjects in the range of 26–81 years old were expanded through passaging and were cryopreserved (Figures 1B and S1C). Differential

Figure 1. Donor-derived airway organoids are stable and distinctive
(A) Workflow of airway organoid generation.
(B) Table showing the age and sex of lung sample donors.
(C) Brightfield images of AOs derived from different donors. Scale bars, 200 mm.
(D) Confocal images (z stack) of whole-mounted organoids. Scale bars, 50 mm. Z stacks are combined into a z stack projection throughout the entire organoid and images are deconvolved to improve noise reduction and enhance contrast.
(E) Spectral flow gating strategy for (1) acTUBA+ as ciliated cells, (2) MUC5AC+ acTUBA− as goblet-like cells, (3) TSPAN8+ MUC5AC− as pre-goblet cells, (4) CD49f+ CD271−, (5) CD49f+ CD271+, (6) CD49f− CD271+ as basal cells, and (7) CD49f− CD271− as undefined cells.
(F) Scheme of cell types observed in the 3D AOs.
(G and H) Pie charts representing AO cell-type composition from indicated donors and indicated passages (G) and from different passages of the same donor (H).
(I–K) Pearson correlation showing the relationship between age of the donor and (I) % of ciliated-like cells (acTUBA+), (J) % of MUCA5AC+ cells, (K) % of CD271+ or CD49f+ cells, in the AOs. Values for Pearson correlation and p values are depicted. Functions of the positive or negative correlations are depicted by the Linear Model with R2 as value to indicate how well the linear model function agrees with the individual data points. If R2 = 0 then 0% of the data points follow the linear model, if R2 = 0.5 then 50% of the data points follow the linear model, and if R2 = 1 then 100% of the data points follow the linear model. For (G) and (K), data (pie chart fraction or dots) represent the mean value of three independent experiments with triplicates for each donor-derived organoid (DDO).

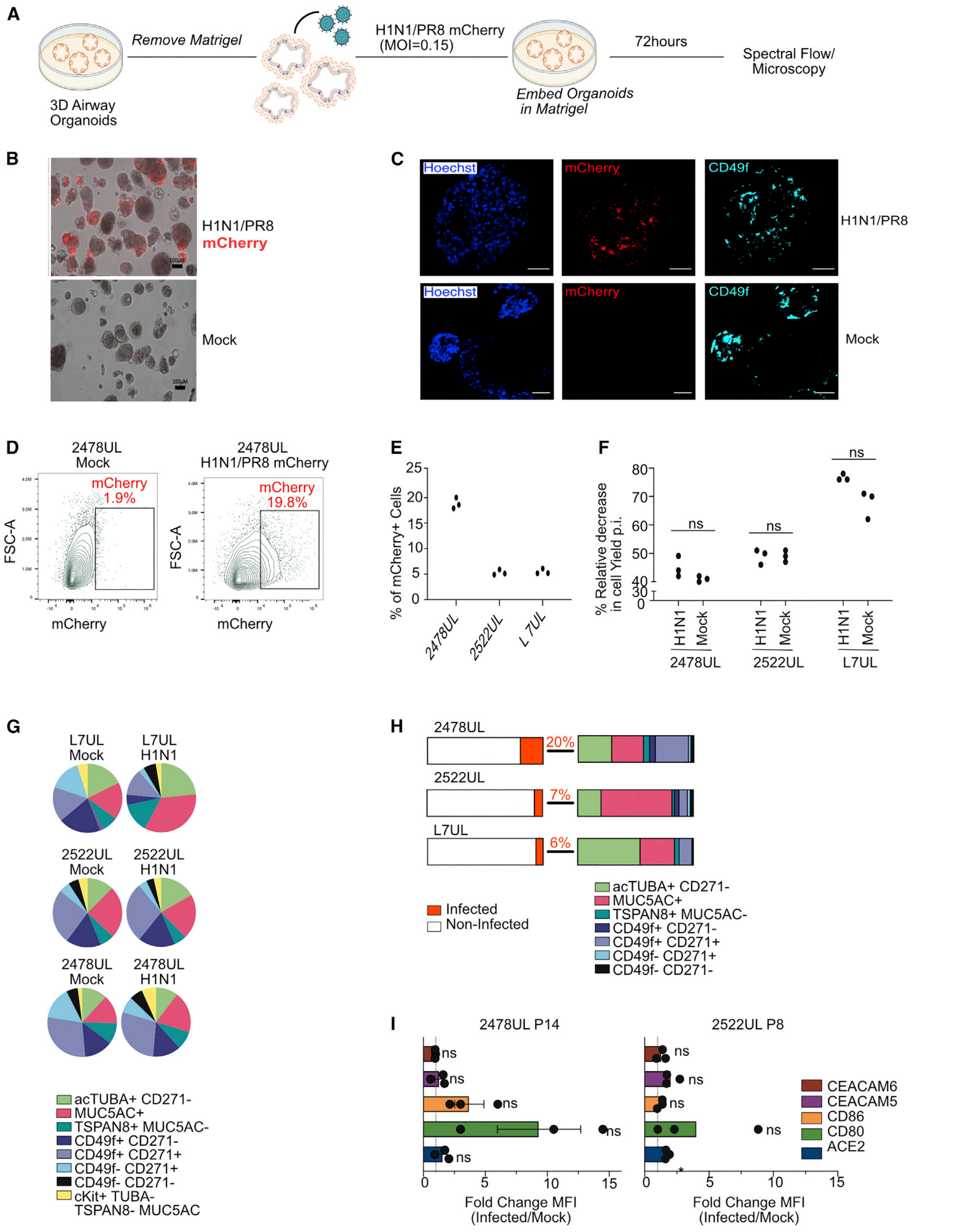

(legend on next page)

interference contrast images revealed growth of AO in Matrigel (Figure 1C) and imaging analysis of AO for acetylated Tubulin (acTUBA) confirmed the presence of ciliated cells (Figure 1D). To assess the cell-type composition and stability of AO in this panel, we performed Spectral flow cytometry analyses (termed “Spectral flow” here) on 14 reported airway epithelial markers (Bonser et al., 2021). Spectral flow enabled cell subset identification (Figures 1E, 1F, S1F, and S1G). Spectral flow revealed composite makeup with seven discrete cell populations in 15 AOs analyzed (Figure 1G). We identified ciliated-*like* cells (marked by $acTUBA^{high}$, $CD271^{neg}$), goblet-*like* cells ($acTUBA^{neg}$, $MUC5AC^{+}$) (Gray et al., 2004), pre-goblet-*like* cells ($acTUBA^{neg}$, $MUC5AC^{+/-}$,$TSPAN8^{+}$), three populations of cells expressing basal cell markers $CD49f^{+}CD271^{+}$, $CD49f^{neg}CD271^{+}$, $CD49f^{+}CD271^{+}$, and a population of $CD49f^{neg}CD271^{neg}acTUBA^{neg}MUC5AC^{neg}$ $TSPAN8^{neg}$ cells. AOs derived from different donors displayed distinct cell-type compositions even though cultured in identical growth factors and environmental conditions (Figure 1G). Furthermore, different passages from the same donor-derived organoid (DDO) retain their patient-specific composition and are stable in composition (Figures 1H and S1A). Likewise, organoids generated from the upper and lower lobes of the lung of the same patient were very similar in makeup (Figure S1B). We generated a biobank of 20 stable, cryopreserved AOs (Figure S1C). Spectral flow for intra-cellular TMPRSS2 (Figure S1D) and extracellular ACE2 (Figure S1E) revealed the fraction of cells expressing these proteins that play critical roles in SARS-CoV-2 entry. Pearson correlations between age and goblet-*like*-, ciliated-*like*-, and basal cells in the organoids were not significant (Figures 1I–1K). So, we generated a stable and expandable biobank of 3D AO and we capitalized on it to understand SARS-CoV-2 infection in airway epithelium of different individuals.

Benchmarking of airway organoid viral infections with H1N1 influenza

To benchmark reproducible viral infection of AOs, we first used H1N1/PR8 virus encoding mCherry (Figures 2A and 2B). Live imaging of whole-mount organoids through confocal microscopy for mCherry and cellular markers confirmed mCherry-positive cells throughout the 3D AO (Figures 2B and 2C). H1N1 infection levels were distinct for 2478UL, 2522UL, and L7UL organoids, but triplicate infections yielded similar infection rates for each individual organoid (Figures 2D and 2E). Live cell numbers in H1N1-infected AOs were similar compared with AOs going through the same procedures with Mock infection (Figure 2F). We used Spectral flow on mCherry and other markers to establish H1N1 tropism in distinct airway epithelial cell subsets, adding cKit as a 15th marker, as this receptor has been suggested to mark airway regeneration upon injury (Fang et al., 2012; Lopez-Giraldo et al., 2018). The Spectral flow strategies (Figure 2D) showed that H1N1/PR8 mCherry virus predominantly infected $acTUBA^{high}/CD271^{-neg}$ ciliated cells and $acTUBA^{neg}/MUC5AC^{+}$ goblet cells (Figure 2H).

Since viral infections trigger interferon-induced gene expression in epithelial cells to orchestrate immune responses (Schleimer et al., 2007), we also stained for co-stimulatory molecules CD80 and CD86 (Kaneko et al., 2000), or immune-activating molecules CEACAM5 and CEACAM6 (Lambrecht and Hammad, 2010). ACE2 is as an interferon-upregulated gene (Ziegler et al., 2020). Upregulation of these cell surface molecules was not significant, 72 h post H1N1/PR8 infection (Figure 2I).

Application of airway organoid viral infection protocols for SARS-CoV-2

We next applied our viral infection protocols with AOs to SARS-CoV-2 WA-1 at 72 h postinfection (p.i.) (Figure 3A). Confocal microscopy analyses of whole-mount organoids revealed the presence of double-stranded RNA (dsRNA) and viral nucleocapsid protein (N) in infected organoids (Figure 3B). It should be noted that removal of organoids from Matrigel induces a reverse of the organoid polarization into an apical-out model (Co et al., 2019), which can be appreciated through the acTUBA staining in Figure 3B.

Figure 2. H1N1 viral infection of airway organoids

(A) Experimental scheme of airway organoid infection with H1N1/PR8 mCherry virus (MOI = 0.15, analyses at 72 h p.i.).

(B) Fluorescent microscopy images of H1N1-infected organoids.

(C) Confocal images (z stack) of whole-mounted AOs, Hoechst (blue): nucleus, mCherry (red): H1N1/PR8+ cells, CD49f (cyan): CD49f+ cells. Scale bar, 50 μm.

(D) Spectral flow layouts of mCherry+ cells.

(E) Quantification of mCherry H1N1/PR8+ in 2,478, 2,522, and L7 organoids.

(F) Quantification of live cell number p.i. compared with prior to infection.

(G) Pie charts of cell populations distribution in AO from different donors p.i.

(H) % of H1N1/PR8 mCherry+ cells (left) and bar charts of the distribution of mCherry-infected cell types (right).

(I) Fold change in median fluorescence intensity (MFI) of CEACAM6, CEACAM5, CD80, CD86. (G) and (H) pie chart fractions show the mean value of three independent experiment with triplicates. For (E) and (F), dots represent the mean values of each independent experiment with triplicates per DDO. For (F) and (H), error bars are SEM. Paired t test, $*p < 0.05$; ns, non-significant was performed.

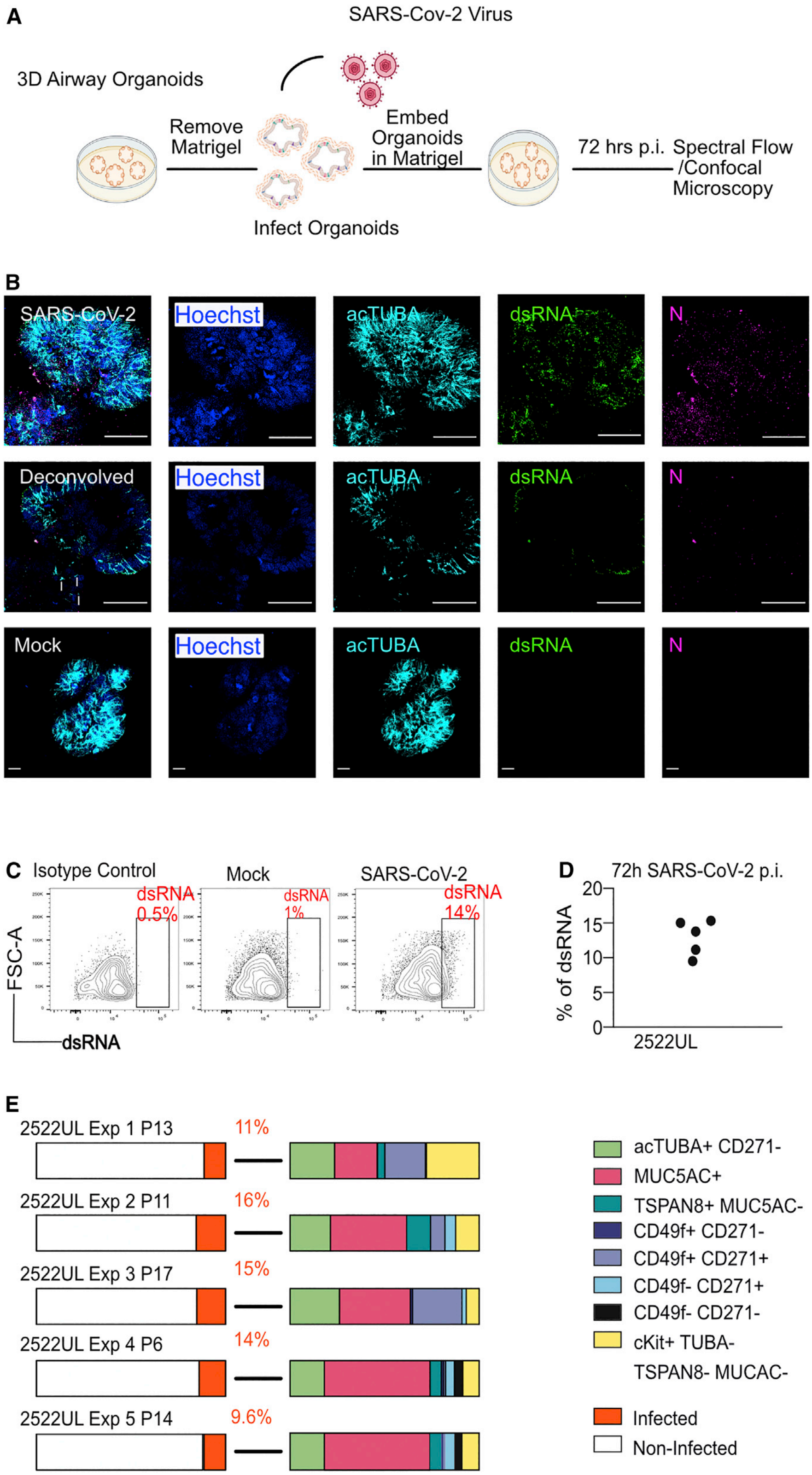

(legend on next page)

Spectral flow analysis on five independent SARS-CoV-2 infections of 2522UL show that infections are consistent (Figures 3C and 3D) and an increase in cKit + cells p.i. (Figure S2A). Gating on dsRNA+ cells showed that ciliated (acTUBA+) and mucus-producing (MUC5A+) cells are predominant cell types carrying replicating SARS-CoV-2 (Figure 3E). The replicate experiments were also analyzed for expression of CEACAM6, CEACAM5, CD80, CD86, and ACE2 at 72h p.i. SARS-CoV-2 WA-1-infected cells were positive for ACE2 and a high fraction of infected cells expressed CD80, CD86, and CEACAM6 (Figure S2B).

Identifying the host cell susceptibility factors to SARS-CoV-2 infection variation with an AO biobank

To uncover the rules of infection with SARS-CoV-2, we selected a panel of 12 AOs (Figure 4A) that captured the diversity in cell composition, age, and sex. Spectral flow of dsRNA staining revealed variation in the percentage of replicating SARS-CoV-2+ cells in the 12 different organoids in 33 separate infections (Figures 4B and 4C). The organoid 2525UL expresses low TMPRSS2 (Figure S1D), providing an explanation for the low infection rate in 2525UL (Figure 4C). We investigated the rules of the remarkable variation in infection rates in the other 11 organoids.

We performed Spectral flow upon infection (Figures 4D and S2C). SARS-CoV-2 infection induces an increased proportion of cells expressing acTUBA (Figure 4E) or cKit (Figure 4F), but no alterations in the fraction of MUC5AC+, $CD49^{+}$, or CD271+, or $CD49f^{neg}CD271^{neg}$ cells (Figures S2D–S2F). The percentage of cell death was similar between SARS-CoV-2 infected and Mock (Figure S2G), implying that cell composition alterations following SARS-CoV-2 WA-1 infection were not caused by the death of specific cell populations in 3D AO.

To investigate parameters that may correlate with efficient infection, we performed principal-component analysis (PCA) for Mock and SARS-CoV-2-infected organoids on 21 variables (cell types, cell death, infection rate, age, sex of the donors) (Figure S3A). PCA (length of lines in Figure S3A) showed that 2450UL and 2522UL reveal alterations to SARS-CoV-2 infection. Depicting PCA of many factors in a circle of correlation, we tested if there are correlations between infection rate, ciliated cells, ACE2 positivity, CD86 positivity, and possibly age (Figure 4G; warm colors and length of arrows that point in the same direction). No single factor on its own, such as age or ACE2-positivity prior to infection, significantly correlated with SARS-CoV-2 infection rate (Figures S3B–S3G). Gating on dsRNA+ cells, we observed the strongest tropism of SARS-CoV-2 for acTUBA+ and MUC5AC+ cells (Figure 4H). The fraction of ACE2+ cells was increased upon SARS-CoV-2 WA-1 infection (Figure 4I). We also observed significant increases in proportions of $CD86^{+}$ cells (Figure S3H) upon SARS-CoV-2 infection, but no increases for CD80-, CEACAM5-, and CEACAM6-expressing cells (Figures S3I–S3L). These data argue against a general, organoid-wide induction of an interferon response program. The Spectral flow of dsRNA+ cells (replicating virus), allowed comparisons between SARS-CoV-2 exposed/infected versus exposed/uninfected and interrogation of functional molecules. Nearly 100% of dsRNA-positive cells were ACE2-positive (Figure 4J), confirming ACE2's critical role for SARS-CoV-2 entry into the cell (Wang et al., 2021). Surprisingly, ACE2+ cell proportions in organoids *prior* to infection did not correlate with eventual SARS-CoV-2 infection rates (Figures 4K and S3G). SARS-CoV-2-infected patients display neutralizing antibodies that are not to Spike's ACE2-binding domain (Brouwer et al., 2020; Chi et al., 2020), and these clinical findings together with the unexplained infection variation in 12 different organoids (Figure 4C) motivated us to search for novel host proteins in airway epithelial cells co-opted by SARS-CoV-2.

TSPAN8 as novel mediator of SARS-CoV-2 infection

We infected four organoids (2522UL, 2450UL, L7UL, and 2524UL) with SARS-CoV-2 WA-1 and performed scRNA-seq. Unsupervised clustering analysis based on most variable gene expression across all cells (Becht et al., 2018), regardless of infection status, identified seven unique cell subsets represented in a UMAP plot (Figure 5A). Based on the top five most differentially expressed genes by cluster (Figure S4A) and published work (Travaglini et al., 2020; Vieira Braga et al., 2019), we assigned relative identities to these seven populations. These scRNA-seq analyses corroborated our Spectral flow results demonstrating that our 3D

Figure 3. Reproducibility of SARS-CoV-2 infection in airway organoids

(A) Experimental scheme of SARS-CoV-2 infection (MOI = 0.3).

(B) Representative, confocal images (z stack) of SARS-CoV-2-infected (top and middle panels) and Mock-infected (bottom panel) whole-mounted organoids. Scale bars, 50 μm. For the top and bottom, all z stacks were combined into one z stack projection throughout the entire organoid image. Middle: 3 z stacks with in-focus acTUBA staining were combined into a z-projection and deconvolved to increase contrast.

(C) Gating layouts of Spectral flow for dsRNA (cells with replicating SARS-CoV-2).

(D) % of dsRNA+ in replicate experiments of the same DDO 72 h p.i. Dots show the mean value of each experiment with three replicates.

(E) % of dsRNA + cells (left) and the bar charts (right) showing the fraction of cell types infected by SARS-CoV-2-WA-1 (dsRNA+). Bar charts show the mean value of three independent experiments with three replicates each.

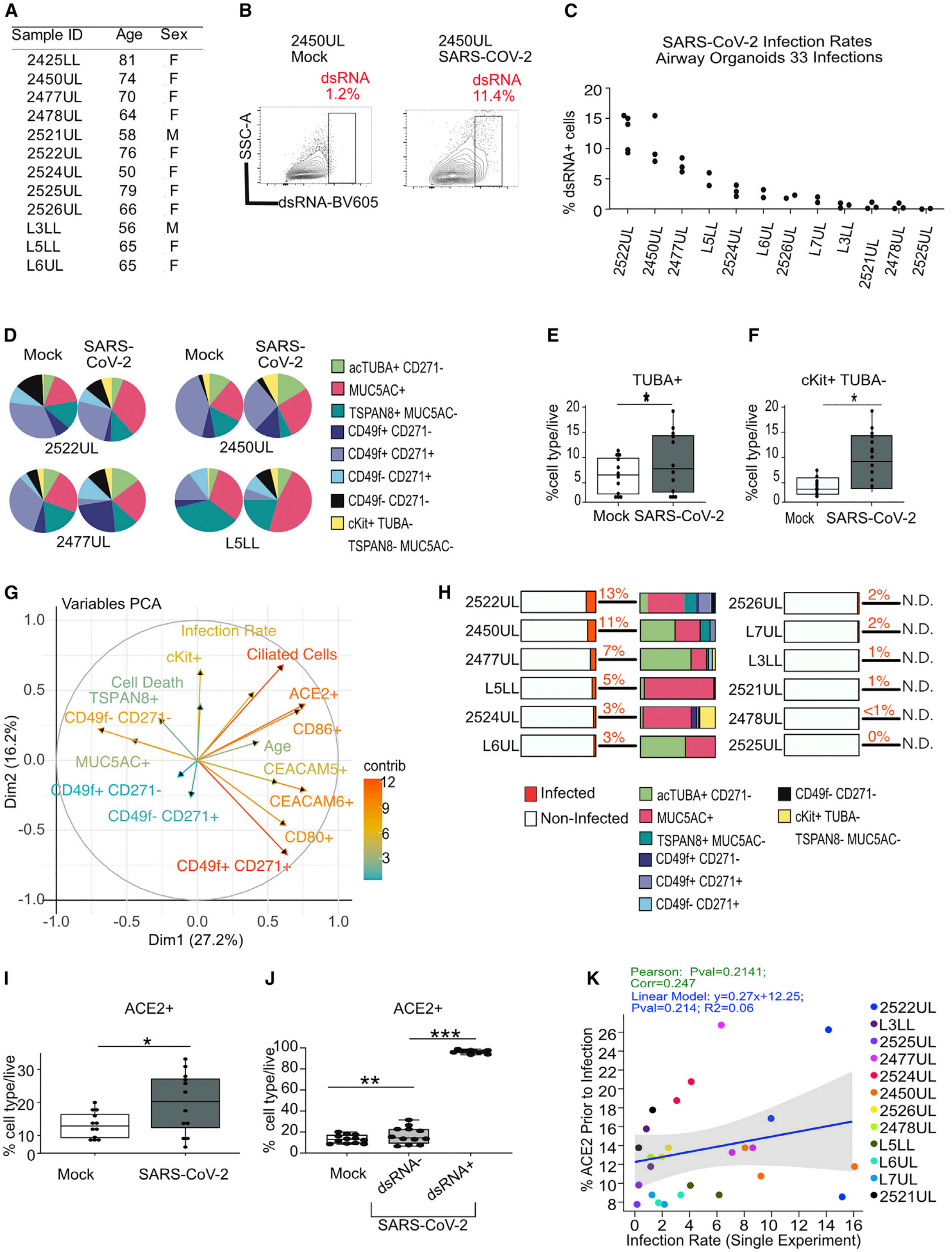

(legend on next page)

AOs have a complex makeup of cell types. It should be noted that specific single-cell characterization of different AOs was not our objective in this study. BSL-3 restrictions prevented us from running the control that we perform in other studies (Gonzalez et al., 2022) and for unknown reasons read counts were relatively low for organoids L7 and 2524. We, therefore, treated the four organoids as one collective dataset for discovery of novel mediators with scRNA-seq resolution. Interrogating cells expressing viral SARS-CoV-2 transcript, we identified specific transcriptomic signatures in infected cells (Figure S4B and Table S2) and genes differentially expressed in single cells, comparing positive for viral read identities with false identities (Figure 5B and Table S3). Neutralizing antibodies in COVID-19 patients suggest the existence of multiple targets (Brouwer et al., 2020; Chi et al., 2020) and we mapped several of these suggested genes back onto our AO UMAP (Figure S4C). For the remainder of this study, we focused on TSPAN8 (Figure 5B, Figure S4C, and Table S3), since members of the TSPAN family have been reported to promote cell entry of different viruses and depletion of TSPAN8, CD9, in mice reduced MERS-CoV lung titers by ~90% (Earnest et al., 2017). In addition, the presence of TSPAN8 in infected cells was documented in supplemental data of a single SARS-CoV-2-infected lung organoid (Lamers et al., 2020), but the role of TSPAN8 in SARS-CoV-2 infection has not been investigated.

scRNA-seq of SARS-CoV-2-infected organoids showed that *TSPAN8* mRNA reads were present in 64% of single cells positive for SARS-CoV-2 reads (size of the circle in Figure 5B). Fortuitously, the anti-TSPAN8 antibody was included in our Spectral flow to distinguish goblet cells ($MUC5AC^{+}TSPAN8^{-}$) from pre-goblet cells ($MUC5AC^{-}$ $TSPAN8^{+}$) (Figure S2), allowing investigation of the role of TSPAN8 surface protein in SARS-CoV-2 infection. The number of TSPAN8-positive cells increased upon SARS-CoV-2 infection (Figure 5C) but decreased upon H1N1/PR8 infection (Figure S5A). As we observed for ACE2 (Figure 4J), most SARS-CoV-2-infected cells expressed TSPAN8 (Figure 5D), whereas H1N1 infected cells do not (Figure 5E). Furthermore, most SARS-CoV-2 infected cells in our AO co-expressed ACE2 and TSPAN8 on the cell surface (Figures 5F and S5B). Different from ACE2 (Figure 4K), the proportion of TSPAN8+ cells *prior* to infection correlated with levels of eventual infection (Figures 5G and S5C), suggesting TSPAN8 somehow facilitates SARS-CoV-2 infection. These correlations also held true for $TSPAN8^{+}ACE2^{+}$ and $TSPAN8^{+}MUC5AC^{-}$ cells prior to infection (Figures 5H and 5I).

In infected patients, *TSPAN8* expression decreases in airway brushes of acute illness in patients caused by non-SARS-CoV-2 respiratory viruses, while airway brushes from COVID-19 patients revealed preservation of *TSPAN8* levels (Figure 5J), despite depletion in goblet cells (Mick et al., 2020). In addition, in the lungs of two COVID-19 patients, we could detect cells that express ACE2 and TSPAN8 concomitantly (Figure 5K). Collectively, the clinical and 3D airway organoid data suggest that TSPAN8 facilitates SARS-CoV-2 infection.

A reductionist 293T platform to investigate TSPAN8

We generated a panel of six HEK 293T cell lines, stably expressing ACE2, TSPAN8, or TSPAN8 CD9 as a control to enable a reductionist approach to investigate TSPAN8

Figure 4. Susceptibility of airway organoids to SARS-CoV-2 infection is not predicted by ACE2
(A) Table showing donor ID, sex, and age of the lung sample donors.
(B) Gating layout of Spectral flow for dsRNA (MOI = 0.3; analyses at 72 h p.i.).
(C) Quantification of the fraction of dsRNA+ cells in SARS-CoV-2-infected AOs.
(D) Pie charts representing the distribution of cell populations in AO from different donors p.i. Each fraction of the pie charts shows the mean value of three independent experiments with triplicates.
(E and F) % of acTUBA+ cells (E) % of acTUBA-cKit+ cells (F) in all organoids combined. Each dot shows the mean value of three independent experiments for each DDO.
(G) PCA of different variables impacting infection with SARS-CoV-2-WA-1. Each arrow corresponds to one biological descriptor; the longer the arrow, the better the representation (the color displays the cos2). Orange to red parameters (warm colors) contribute to differences between Mock and infected organoids. Parameters that correlate with each other are presented by arrows going in the same direction. Three independent experiments with at least three replicates per experiment were performed per DDO.
(H) Stack bar charts representing the mean value from three experiments of dsRNA+ cells (in red) in infected organoids (left) and the fraction of cell types infected by SARS-CoV-2 WA-1 for each DDO.
(I) % of ACE2+ cells in Mock condition (non-exposed to the virus), and in SARS-CoV-2 condition. For SARS-CoV-2 condition, the % of ACE2+ cells is shown in the fraction of $dsRNA^{neg}$ (exposed non-infected cells) and in the fraction $dsRNA^{pos}$ (infected cells). In (I) and (J), dots show the mean of the cell population for three experiments for each DDO.
(K) Linear regression modeling the relationship between infection rate and % ACE2+ cells prior to infection. Each point represents the % cell type for the distinct donor. The mean values of each independent infection experiment are depicted and plotted. For this figure, analyses are done 72 h SARS-CoV-2 WA-1 infection at MOI = 0.3. For (E), (F), and (I) Wilcoxon signed-rank test, and for (J) Friedman test were performed, $^{**}p < 0.01$; $^{***}p < 0.001$, ns, non-significant.

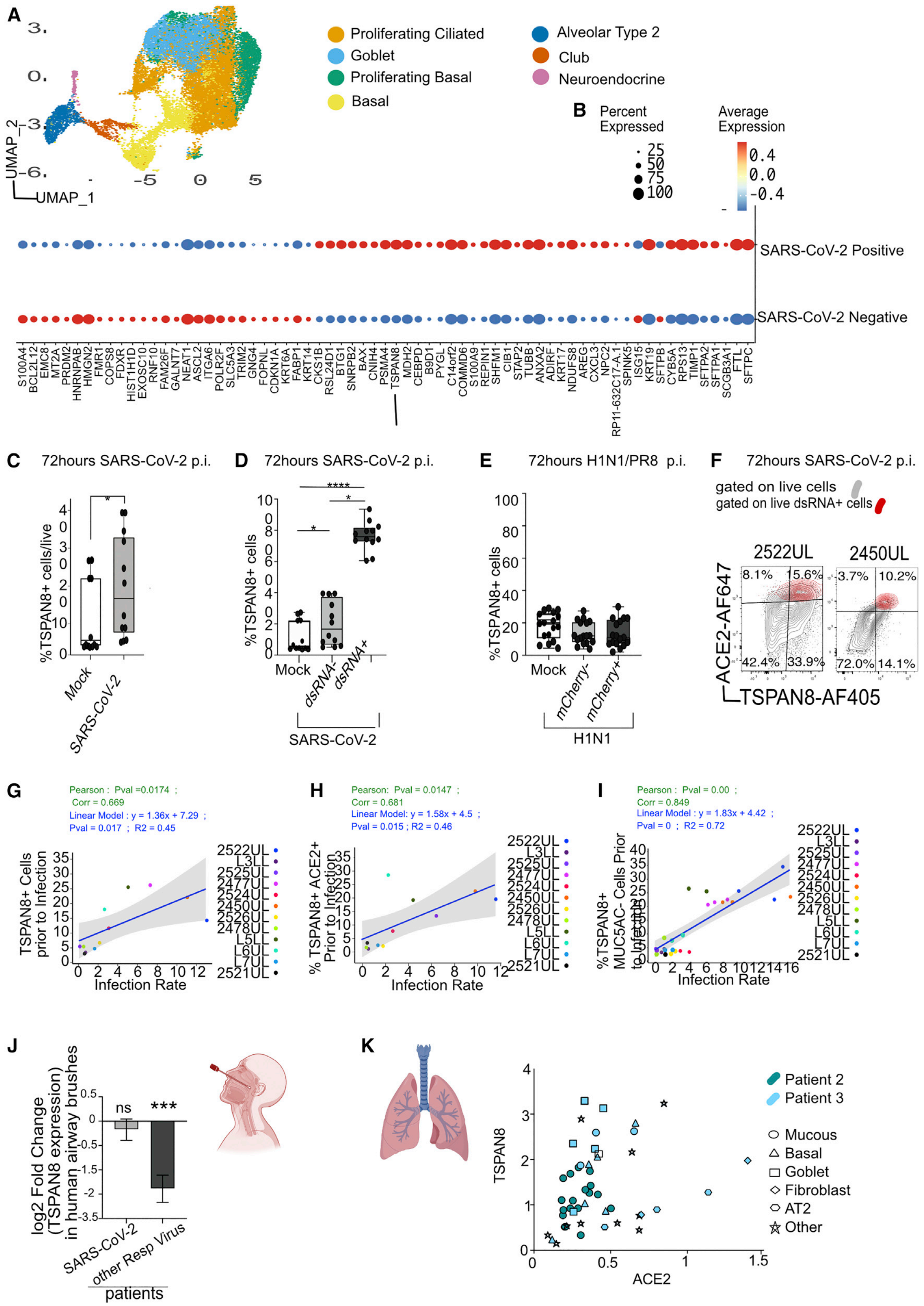

(legend on next page)

(Figure 6A). HEK 293T cells do not express extracellular ACE2, TSPAN8, or CD9. The expression of TSPAN8 or CD9 did not alter ACE2 expression levels (Figures S6A and S6B) or overall subcellular localization (data not shown). We used these cell lines to assess viral entry with designed, replication-deficient, luciferase-expressing pseudo-viruses (Ps-virus) expressing Spike of SARS-CoV-2 WA-1, or Delta, or Omicron, or VSV-G (vesicular stomatitis virus-G) (Figures 6A and S6C). VSV-G does not use ACE2 for the entry and serves as control (Finkelshtein et al., 2013).

Entry of Omicron Spike Ps-virus in ACE2 HEK293T, with or without TSPAN8 or CD9, was relatively inefficient (Figures 6B and S6D), which may reflect the altered usage of proteases by Omicron (Meng et al., 2022). Entry of WA-1 (D614G) Spike-, Delta Spike-, and VSV-G- carrying Ps-virus was robust (Figures 6B and S6D). Expression of TSPAN8 or CD9 with ACE2 in HEK 293T cells resulted in roughly 2-fold increases in luciferase activity, but without specificity for Spike or VSV-G Ps-viruses (Figures 6B and S6D). So, TSPAN8 and CD9 enhance viral entry independently of the Spike/ACE2. These Ps-virus-based results are in line with previous reports that TSPANs promote the entry of multiple viruses (Earnest et al., 2015; Hantak et al., 2019).

We next analyzed live virus infection. Phylogenic and genetic analyses have shown that SARS-CoV-2 variants differ not only in their Spike protein but also in other proteins (Thorne et al., 2022) (Figures 6C and S6E). We propagated SARS-CoV-2 WA-1, Delta, and Omicron variants in Vero E6 cells (Figure S6E) with similar efficiency (Figure S6F) and infected the HEK293T cells (Figure 6D). None of the SARS-CoV-2 variants were able to infect TSPAN8- or CD9-expressing HEK293T without ACE2 (Figures 6F and S6G). So, TSPAN8 or CD9 are not alternative entry receptors. Levels of nucleocapsid+ HEK293T cells were unexpectedly modest for SARS-CoV-2 Delta but at expected levels for Omicron (Figure 6G) even though initial entry (Ps-virus) is inefficient (Figure 6B). The presence of TSPAN8 and CD9 in HEK293T cells increased Nucleocapsid+ cells for Omicron infections (Figure 6G). Only TSPAN8, but not CD9, was responsible for an increase of the Nucleocapsid+ cells for WA-1. Of note, the different SARS-CoV-2 variants did not differentially impact HEK293T viability (Figure S6H). Collectively, the HEK293T approach revealed that TSPAN8 facilitates SARS-CoV-2 infection rates independently of Spike/ACE2 interaction.

AOs reveal conserved use of TSPAN8 by SARS-CoV-2 AW-1, Delta, and Omicron variants

Last, we investigated the infection characteristics of SARS-CoV-2 WA-1, Delta, and Omicron variants in the context of 3D AO with their diverse cell-type composition and subsequently related results to TSPAN8. We used organoids 2522UL and 2450UL in head-to-head comparisons, which demonstrated highest infection with the WA-1 variant at 72 h p.i. (Figure 7A). SARS-CoV-2 variants elicited distinct effects on cell composition (Figures 7B and S7A–S7E). A selective increase in MUC5AC+ cells with Omicron (Figure 7C), as well as in cKit+ cells with WA-1 infection

Figure 5. Discovery of TSPAN8 in SARS-CoV-2-infected airway organoids

(A) UMAP reduction on the merged cell data with overlaid clusters and identified cell types.

(B) Top differentially upregulated genes, including *TSPAN8* (arrow), within a representative random subsample of SARS-CoV-2-positive (top) versus SARS-CoV-2 negative (bottom) cells. Circle sizes indicate the % of cells within the total cell population that the specific gene is expressed.

(C) Box and whisker plots representing the % of TSPAN8-positive cells in AOs for Mock and SARS-CoV-2 condition at 72 h p.i. (MOI = 0.3).

(D) Box and whisker plots representing the % of TSPAN8+ cells in AOs for Mock and SARS-CoV-2-infected conditions. For the SARS-CoV-2 condition, the % of TSPAN8+ cells is quantified in non-infected (dsRNA−) and infected (dsRNA+) cells.

(E) Same as (D) but for mCherry H1N1/PR8-infected organoids.

(C–F)Dots show the mean value of three independent experiments with three replicates each for each DDO. Wilcoxon signed-rank test, $^{*}Pv < 0.05$ was performed.

(F) Representative Spectral flow plots of overlay between dsRNA cells (in gray) and SARS-CoV-2-infected, dsRNA+ cells (in red) in AOs for 2,522 organoid. The x axis represents TSPAN8 expression intensity and the y axis represents ACE2 expression intensity.

(G–I) ScatterPlot showing the relationship among (G) % of TSPAN8+ cells, (H) TSPAN8+ ACE2+, (I) TSPAN8+ MUC5AC− prior to infection and the infection rate. Pearson correlation statistical significance stated on graph (Pval.).

(J) Differential expression of TSPAN8 in nasal swabs of adult patients with acute respiratory illness (ARI) due to COVID-19 (n = 93) or other viral infection (n = 41), in comparison to patients with ARI due to non-viral etiology (n = 100). Pearson's chi-squared test, $^{***}Pv < 0.001$, ns, non-significant.

(K) Single-cell sequencing was performed to analyze AOs from two different donors from patients undergoing lobectomy for focal airway tumors (Travaglini et al., 2020). The graph shows cells expressing TSPAN8 and ACE2 from different patients. Of the 60,993 cells derived from airway tissue of three patient donors in this dataset, 48 cells were found with at least one unique molecular identifier (UMI) for both genes. Only one cell derived from patient 1, which had fewer cells sequenced overall and so we excluded it. The expression values represent ln(UMI-per-10K + 1) in each of the 47 cells from patients 2 and 3. Cell-type designations were determined by Travaglini et al. (2020).

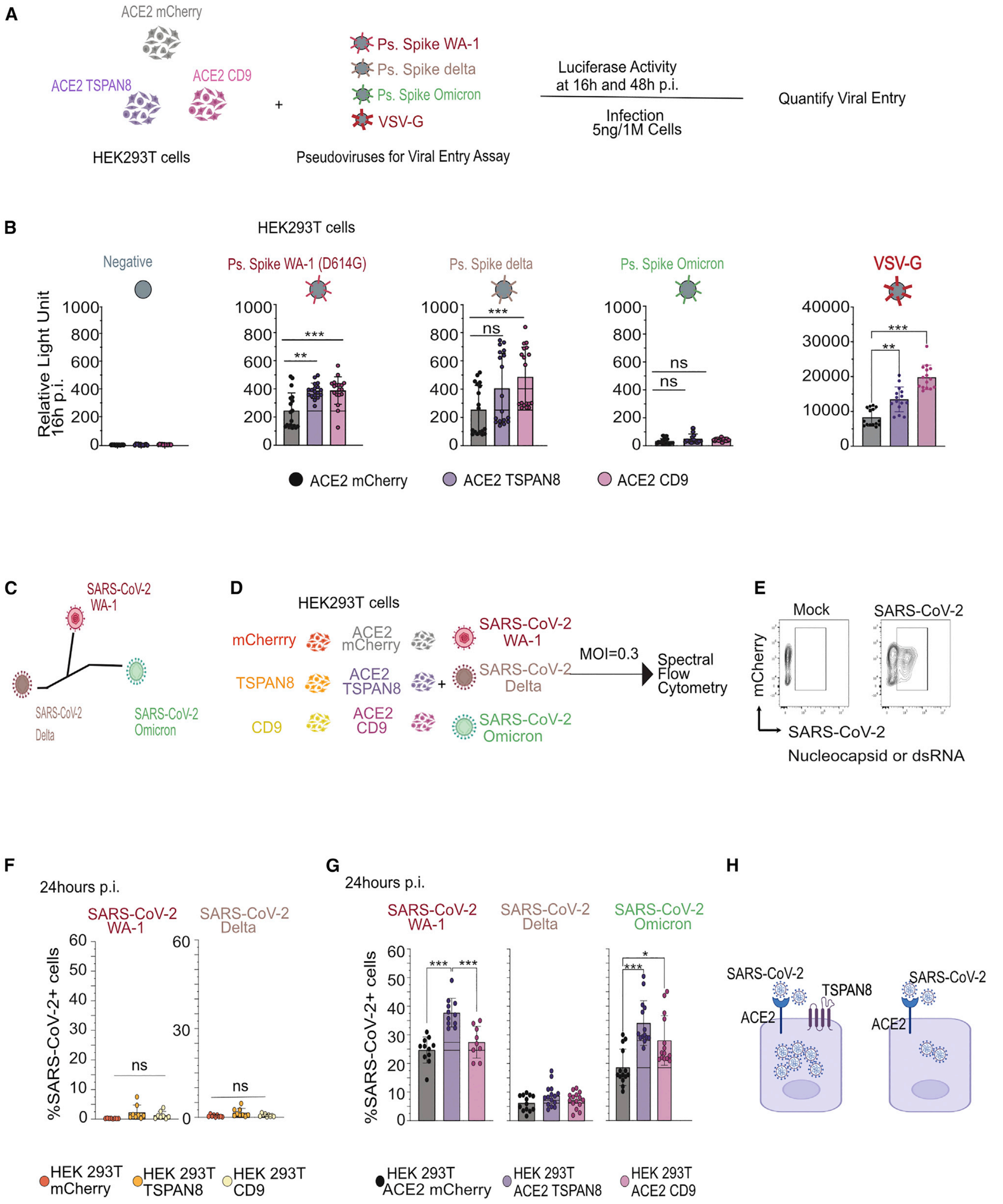

(legend on next page)

(Figure S7C) was noted. Analysis of functional markers demonstrated stepwise increases in ACE2+ and CD86^{+} cells for the WA-1, Delta, or Omicron variants (Figures 7D and S7F). CD86 (Corbiere et al., 2011) and ACE2 (Ziegler et al., 2020) are interferon-stimulated genes implying that SARS-CoV-2 VOC trigger increasing strengths of interferon responses in airway epithelial organoids. The fraction of TSPAN8+ cells increased following all SARS-CoV-2 variant infections, but there was no difference between the VOCs (Figure 7E).

Our AOs do not contain immune cells. With the notion that our organoids are a purely epithelial cell platform, it is striking that the percentage of infected cells in AOs decreased stepwise, in head-to-head comparisons of WA-1, to Delta, to Omicron (Figure 7F). Analyzing the dsRNA-positive cells with replicating virus, SARS-CoV-2 AW-1, Delta, and Omicron all displayed clear tropism for acTUBA+ (ciliated) and MUC5AC+ (goblet) cells (Figures 7G and S7G). For all SARS-CoV-2 variants, close to 100% of infected cells (dsRNA+) displayed ACE2 expression (Figures 7H and S7H), highlighting the role of ACE2 as an entry receptor (Hoffmann et al., 2020b). Roughly 80% of all SARS-CoV-2-infected cells expressed TSPAN8 (Figures 7I and S7I), indicating that TSPAN8 is critical as well. While AO cell infection rates decreased sequentially in SARS-CoV-2 from WA-1 to Delta to Omicron variants (Figure 7I), the presence of TSPAN8 in infected cells remained conserved (Figure 7J). The conserved role of TSPAN8 for all SARS-CoV-2 VOCs imply that TSPAN8 is an attractive therapeutic target to limit COVID-19, which we explored with a TSPAN8-blocking antibody approach developed in the oncology field (Bonnet et al., 2019; Kim et al., 2015). Infection levels of 2522UL and 2450UL organoids with SARS-CoV-2 WA-1 were reduced by 60% upon TSPAN8-blocking antibody treatment (Figures 7K, S7J, and S7K), demonstrating that TSPAN8 plays a functional role in SARS-CoV-2 infection.

DISCUSSION

SARS-CoV-2 research has focused on the immune cells (Schultze and Aschenbrenner, 2021; Sette and Crotty, 2021), but many questions regarding airway epithelial responses to SARS-CoV-2 infection have remained unanswered. Clonal cell lines are efficient discovery tools but lack variations in genetic and proteomic traits. Lung epithelial organoids contain diverse cell types (Sachs et al., 2019). Here, we characterized AO from many subjects and capitalized on the diversity and cell-type-complexity in our large panel of AOs to (1) understand the underpinnings of epithelial cell infection by SARS-CoV-2 WA-1, Delta, and Omicron variants, and (2) discover TSPAN8 as a conserved mediator of infection with all three variants.

Our study with a comprehensive panel of AOs capitalized on the unique diversity in AOs derived from different subjects to understand airway epithelial characteristics that impact SARS-CoV-2 infection. Essential here was the Spectral flow approach that allows cell-type characterization. Our AOs from our model are embedded in Matrigel as we cannot obtain the high throughput for the study presented here with organoids in air-liquid interface (Sachs et al., 2019). SARS-CoV-2 WA-1, Delta, and Omicron variants shared highest tropism for ciliated and goblet cells. The three SARS-CoV-2 variants elicited distinct cell-type composition effects in AOs; selective increases in MUC5AC+ cells for Omicron and cKit+ cells for WA-1 were striking. MUC5AC and cKit are both suggested to play a role in airway epithelium regeneration (Letuve et al., 2019; Xian and McKeon, 2012), indicating different regeneration responses in the AOs triggered by different strains. Interferons upregulate CD80, CD86 (Kaneko et al., 2000), CEACAM5, and CEACAM6 (Lambrecht and Hammad, 2010) and ACE2 itself (Ziegler et al., 2020). ACE2, CD80, CD86, and CAECAM6 (Figures S3H–S3L and 4I) expression levels were upregulated in infected cells

Figure 6. TSPAN8 serves as a facilitator for SARS-CoV-2 WA-1, Delta, and Omicron variants

(A) Scheme of workflow for infection HEK293T cell lines with the Ps-virus expressing Spike protein of SARS-CoV-2 WA-1 (USA-WA1/2020) or SARS-CoV-2 Delta (SARS-CoV-2 B1.1617.2) or SARS-CoV-2 (Omicron BA1.1.529) or VSV-G virus (as control). Viral entry is quantified 16 h or 48 h postinfection by measuring luciferase activity.

(B) Luminescence (relative light unit) measured as a function of Ps-virus entry for the backbone Ps-virus, WA-1, Delta, Omicron Ps-virus and VSV-G at 16 h p.i.

(C) Scheme of SARS-CoV-2 phylogenetic tree representing SARS-CoV-2 WA-1, Delta, and Omicron.

(D) Workflow of HEK293T cell lines infection with live SARS-CoV-2.

(E) Flow cytometry plot of Nucleocapsid+ cells post SARS-CoV-2 infection.

(F) SARS-CoV-2 Nucleocapsid+ fraction in mCherry, TSPAN8, and CD9 HEK293T cell at 24 h post SARS-CoV-2 WA-1 or Delta infection (MOI = 0.3).

(G) SARS-CoV-2 Nucleocapsid+ cells fraction in ACE2 mCherry-, ACE2 TSPAN8-, and ACE2 CD9-expressing HEK293T cells at 24 h post SARS-CoV-2 WA-1, Delta, or Omicron infection (24 h p.i., MOI = 0.3).

(F–H) Three independent experiments with four replicates. Nonparametric ANOVA tested corrected by Geisser Greenhouse Correction was performed to compare between different conditions. *Pv < 0.05; ***Pv < 0.001.

(H) Scheme showing TSPAN8 as a facilitator of infection.

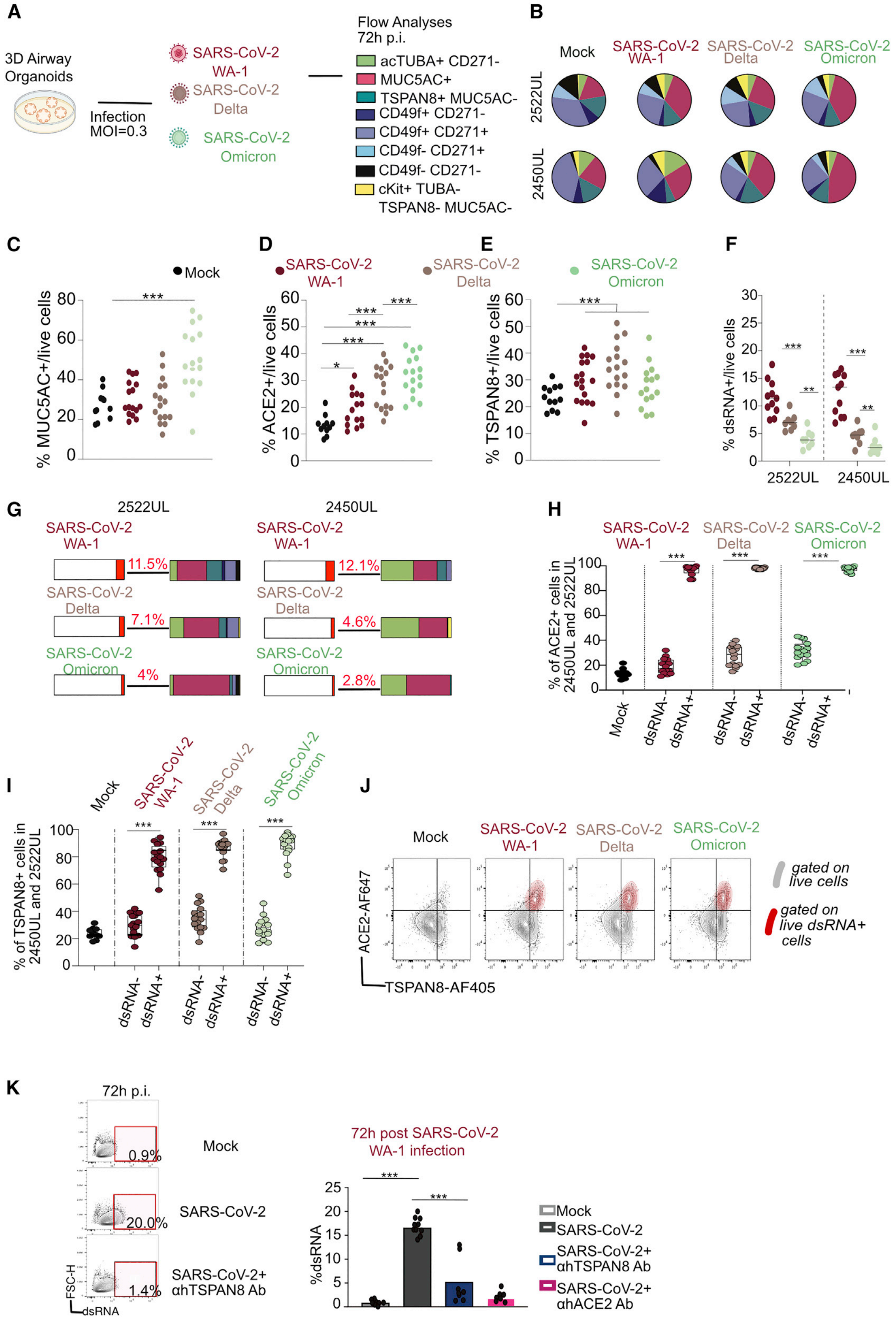

(legend on next page)

(dsRNA-positive), but not in all cells in the epithelial AO, arguing against an organoid-wide interferon response. With that notion, it was remarkable that WA-1, Delta, or Omicron variants yielded stepwise increases in ACE2- and CD86-positive cells, suggesting that SARS-CoV-2 VOC trigger stronger interferon responses in airway epithelial organoids than Ancestral WA-1.

Neutralizing antibodies from COVID-19 patients have multiple targets (Brouwer et al., 2020; Chi et al., 2020), suggesting that these protective antibodies may block interactions of cell surface molecules other than the Spike protein-ACE2 receptor pair. The WA-1, Delta, or Omicron variants all led to increases in TSPAN8-positive cells in AOs. In addition, more than 80% of all SARS-CoV-2-infected cells expressed TSPAN8.

TSPAN8 proteins have four *trans*-membrane domains that form lateral associations with multiple molecular partners and with each other, organizing the surface membrane proteins in a dynamic microdomain (Hantak et al., 2019; Kummer et al., 2020). TSPAN8s promote the entry of multiple viruses, including influenza A virus, human cytomegalovirus (HCMV), human papillomavirus, etc (Hantak et al., 2019). We demonstrated that neither TSPAN8, nor CD9, can function as an alternative entry receptor in HEK293T cells. TSPAN8 and CD9 increase entry of Ps-viruses in HEK293T cells, but not via the ACE2 receptor.

We have previously reported in cancer cell lines that TSPAN8 increases extracellular vesicle or exosome (EV) numbers and influences their composition (Nazarenko et al., 2010; Voglstaetter et al., 2019). EV from virus-infected cells can infect or prime to neighbor cells (Martins and Alves, 2020). Circulating EVs have been implicated in SARS-CoV-2 infection (Barberis et al., 2021). We think that TSPAN8 contributes to the spreading of infection through EVs in the case of SARS-CoV-2 infection. Future work is required to understand how TSPAN8, CD9, or other TSPAN8s are involved in SARS-CoV-2 infection. We demonstrated that the addition of a TSPAN8-blocking antibody to the AO prior to infection decreased SARS-CoV-2 infection. VOCs escape from the therapeutic antibody neutralization (Planas et al., 2022; Sigal, 2022; VanBlargan et al., 2022), it will be of value to have other avenues of interfering with SARS-CoV-2. In conclusion, our study demonstrates that donor-derived AOs can be used to model the spectrum of the response of the human airway epithelium to airway pathogens and identify novel therapeutic targets.

EXPERIMENTAL PROCEDURES

Resource availability

Corresponding author

jeroen.roose@ucsf.edu.

Materials availability

A list of materials used and detailed methods are found on the Supplementary Materials section. Materials are available upon request.

Data and code availability

Resources: GEO: GSE211562.

SUPPLEMENTAL INFORMATION

Supplemental information can be found online at https://doi.org/10.1016/j.stemcr.2023.01.011.

AUTHOR CONTRIBUTIONS

L.H. and J.P.R.: conceived the study. L.H., S.L., and K.K.: Spectral flow. L.H., K.K., S.L., O.M.G.: organoid biobank. L.H., M.M., and L.R.: BSL3 work. C.A., A.A.R., and A.J.C.: scRNA-seq. J.C.L.: statistical analyses. C.A., L.C.L.: scRNA-seq analyses. L.R.B. and D.J.E.: advice on airway populations. N.K.S. and M.K.: H1N1 virus. L.H., S.L., O.M.G., and K.B.: microscopy. L.H., S.L., K.B.: HEK239T cell line creation. J.Z.L., V.D., S.M., M.M.: patient samples. G.K., D.M.J., M.M., A.J.C.: funding. M.O.: SARS-CoV-2 virus, and

Figure 7. Conserved role of TSPAN8 in SARS-CoV-2 WA-1, Delta, and Omicron

(A) Workflow of organoid infection by SARS-CoV-2 WA-1, Delta, and Omicron infection (MOI = 0.3, 72 h p.i.).

(B) Pie charts of cell populations distribution in Mock, with SARS-CoV-2 WA-1-, -Delta-, or Omicron-infected AOs. Each fraction of the pie chart shows the mean of three independent experiments with triplicates.

(C–F) % of cells fraction in live cells for Mock or SARS-CoV-2 WA-1-, Delta-, or Omicron-infected AO: MUC5AC+ (C), ACE2+ (D), TSPAN8+ (E), dsRNA+ (F). Dots show the data for each replicate. Experiments are repeated three times with at least three replicates. Nonparametric ANOVA tested corrected by Geisser Greenhouse Correction was performed to compare different conditions. ***Pv < 0.001.

(G) Stack bar charts representing the mean value for three independent experiments of dsRNA+ cells (in red) in SARS-CoV-2-infected organoids (left) and the fraction of cell types infected by SARS-CoV-2 WA-1, Delta, or Omicron (dsRNA+).

(H) Plots representing the % of ACE2+ cells analyzed by Spectral flow in live cells for Mock, SARS-CoV-2 WA-1, Delta, or Omicron conditions. For SARS-CoV-2-infected organoids, the fraction of ACE2+ cells is shown in exposed, non-infected cells (dsRNA−) and infected cells (dsRNA+).

(I) As in (H) but for TSPAN8-positive cells.

(J) Representative Spectral flow plots of overlaid dsRNA− cells (in gray) and dsRNA+ cells (in red) of infected organoids with SARS-CoV-2 WA-1, Delta, or Omicron. The x axis represents TSPAN8 expression intensity and y axis represents ACE2 expression intensity.

(K) Spectral flow of dsRNA+ cells in organoids pre-treated with Mock (TSPAN8 isotype control), TSPAN8, and/or ACE2 blocking antibody (50 μg/mL) at 72 h post SARS-CoV-2 WA-1 infection (MOI = 0.3).

SARS-CoV-2 pseudo-viruses. S.L., M.P.: pseudo-virus infections. J.R.K.: surgery airway samples, clinical data discussion. L.H., S.L., J.P.R.: manuscript writing. J.P.R.: funding. G.K.F., D.M.J., M.M., M.O., M.M., A.J.C., D.E., A.N.S., and J.R.K.: edits on the manuscript.

ACKNOWLEDGMENTS

We thank Drs. L. Ferreira, V. Arce-Gorvel, J-P. Gorvel, and the Roose lab for discussions; Drs. H. Clevers, R. Vries, B. Ramezanpour, and S. Boj for help on organoid technology, CYTEK Aurora for the Spectral flow analyses; Garcia lab (Stanford) for the R-spondin; Dr. S. Memet for help on transfections. Most of the work was supported by an Administrative COVID-19 Supplement 3P01AI091580-09S1 (to J.P.R.) on the parent NIH/NIAID P01-AI091580 (Weiss). Organoids were generated in the Roose Organoid D2B unit, started through a UCSF PBBR TMC (Technologies, Methodologies, and Cores) grant and a gift from the Pathology Department and now, in part, funded via a Mark Foundation for Cancer Research Endeavor Program (all to J.P.R.). The UCSF CoLabs are in part supported by the Bakar ImmunoX Initiative. J.R.K. is funded by the UCSF Bakar ImmunoX Initiative, the UCSF Helen Diller Family Cancer Center, and the American Association for Thoracic Surgery Foundation. D.M.J. is funded by a private endowment fund. Human Frontier Science Program Fellowship LT000061/2018-L to N.K.S. Additional funds gave a grant from the Innovative Genomics Institute and UCSF PBBR (M.O.). M.O. thanks the Rodenberry Foundation, NIH grant R37AI083139, and the Gladstone Institutes for their support. We thank the Core Facility Signaling Factory of the University Freiburg for the cloning service of the plasmids. D.J.E. and L.R.B. are supported by NIH grants U19 AI077439 and R35 HL145235. A.S. is supported by the Bill and Melinda Gates award INV-018944, National Institutes of Health award R01 AI138546, and South African Medical Research Council award 6084-CO-AP-2020.

CONFLICT OF INTERESTS

The authors have no competing interests. A patent "*INHIBITORS OF SARS-COV-2 INFECTION AND OTHER CORONA VIRUS INFECTIONS*" SF-2021-201-2-PCT-0 has been filed.

Received: December 12, 2022
Revised: January 23, 2023
Accepted: January 24, 2023
Published: February 23, 2023

REFERENCES

Barberis, E., Vanella, V.V., Falasca, M., Caneapero, V., Cappellano, G., Raineri, D., Ghirimoldi, M., De Giorgis, V., Puricelli, C., Vaschetto, R., et al. (2021). Circulating exosomes are strongly involved in SARS-CoV-2 infection. Front. Mol. Biosci. *8*, 632290.

Bastard, P., Gervais, A., Le Voyer, T., Rosain, J., Philippot, Q., Manry, J., Michailidis, E., Hoffmann, H.H., Eto, S., Garcia-Prat, M., et al. (2021). Autoantibodies neutralizing type I IFNs are present in ~4% of uninfected individuals over 70 years old and account for ~20% of COVID-19 deaths. Sci. Immunol. *6*, eabl4340.

Becht, E., McInnes, L., Healy, J., Dutertre, C.A., Kwok, I.W.H., Ng, L.G., Ginhoux, F., and Newell, E.W. (2018). Dimensionality reduction for visualizing single-cell data using UMAP. Nat. Biotechnol. *37*, 38–44.

Bonnet, M., Maisonial-Besset, A., Zhu, Y., Witkowski, T., Roche, G., Boucheix, C., Greco, C., and Degoul, F. (2019). Targeting the tetraspanins with monoclonal antibodies in oncology: focus on Tspan8/Co-029. Cancers *11*, 179.

Bonser, L.R., Koh, K.D., Johansson, K., Choksi, S.P., Cheng, D., Liu, L., Sun, D.I., Zlock, L.T., Eckalbar, W.L., Finkbeiner, W.E., and Erle, D.J. (2021). Flow-cytometric analysis and purification of airway epithelial-cell subsets. Am. J. Respir. Cell Mol. Biol. *64*, 308–317.

Brouwer, P.J.M., Caniels, T.G., van der Straten, K., Snitselaar, J.L., Aldon, Y., Bangaru, S., Torres, J.L., Okba, N.M.A., Claireaux, M., Kerster, G., et al. (2020). Potent neutralizing antibodies from COVID-19 patients define multiple targets of vulnerability. Science *369*, 643–650.

Chi, X., Yan, R., Zhang, J., Zhang, G., Zhang, Y., Hao, M., Zhang, Z., Fan, P., Dong, Y., Yang, Y., et al. (2020). A neutralizing human antibody binds to the N-terminal domain of the Spike protein of SARS-CoV-2. Science *369*, 650–655.

Chua, R.L., Lukassen, S., Trump, S., Hennig, B.P., Wendisch, D., Pott, F., Debnath, O., Thürmann, L., Kurth, F., Völker, M.T., et al. (2020). COVID-19 severity correlates with airway epithelium-immune cell interactions identified by single-cell analysis. Nat. Biotechnol. *38*, 970–979.

Co, J.Y., Margalef-Català, M., Li, X., Mah, A.T., Kuo, C.J., Monack, D.M., and Amieva, M.R. (2019). Controlling epithelial polarity: a human enteroid model for host-pathogen interactions. Cell Rep. *26*, 2509–2520.e4.

Cobey, S., Larremore, D.B., Grad, Y.H., and Lipsitch, M. (2021). Concerns about SARS-CoV-2 evolution should not hold back efforts to expand vaccination. Nat. Rev. Immunol. *21*, 330–335.

Corbière, V., Dirix, V., Norrenberg, S., Cappello, M., Remmelink, M., and Mascart, F. (2011). Phenotypic characteristics of human type II alveolar epithelial cells suitable for antigen presentation to T lymphocytes. Respir. Res. *12*, 15.

Earnest, J.T., Hantak, M.P., Li, K., McCray, P.B., Jr., Perlman, S., and Gallagher, T. (2017). The tetraspanin CD9 facilitates MERS-coronavirus entry by scaffolding host cell receptors and proteases. PLoS Pathog. *13*, e1006546.

Earnest, J.T., Hantak, M.P., Park, J.E., and Gallagher, T. (2015). Coronavirus and influenza virus proteolytic priming takes place in tetraspanin-enriched membrane microdomains. J. Virol. *89*, 6093–6104.

Fang, S., Wei, J., Pentinmikko, N., Leinonen, H., and Salven, P. (2012). Generation of functional blood vessels from a single c-kit+ adult vascular endothelial stem cell. PLoS Biol. *10*, e1001407.

Fiege, J.K., Thiede, J.M., Nanda, H.A., Matchett, W.E., Moore, P.J., Montanari, N.R., Thielen, B.K., Daniel, J., Stanley, E., Hunter, R.C., et al. (2021). Single cell resolution of SARS-CoV-2 tropism, antiviral responses, and susceptibility to therapies in primary human airway epithelium. PLoS Pathog. *17*, e1009292.

Finkelshtein, D., Werman, A., Novick, D., Barak, S., and Rubinstein, M. (2013). LDL receptor and its family members serve as the cellular receptors for vesicular stomatitis virus. Proc. Natl. Acad. Sci. USA *110*, 7306–7311.

Gonzalez, H., Mei, W., Robles, I., Hagerling, C., Allen, B.M., Hauge Okholm, T.L., Nanjaraj, A., Verbeek, T., Kalavacherla, S., van Gogh, M., et al. (2022). Cellular architecture of human brain metastases. Cell *185*, 729–745.e20.

Gray, T., Coakley, R., Hirsh, A., Thornton, D., Kirkham, S., Koo, J.-S., Burch, L., Boucher, R., and Nettesheim, P. (2004). Regulation of MUC5AC mucin secretion and airway surface liquid metabolism by IL-1β in human bronchial epithelia. Am. J. Physiol. Lung Cell Mol. Physiol. *286*, L320–L330.

Han, Y., Duan, X., Yang, L., Nilsson-Payant, B.E., Wang, P., Duan, F., Tang, X., Yaron, T.M., Zhang, T., Uhl, S., et al. (2021). Identification of SARS-CoV-2 inhibitors using lung and colonic organoids. Nature *589*, 270–275.

Hantak, M.P., Qing, E., Earnest, J.T., and Gallagher, T. (2019). Tetraspanins: architects of viral entry and exit platforms. J. Virol. *93*, 014299-17.

Harvey, W.T., Carabelli, A.M., Jackson, B., Gupta, R.K., Thomson, E.C., Harrison, E.M., Ludden, C., Reeve, R., Rambaut, A., et al.; COVID-19 Genomics UK COG-UK Consortium (2021). SARS-CoV-2 variants, spike mutations and immune escape. Nat. Rev. Microbiol. *19*, 409–424.

Hoffmann, M., Kleine-Weber, H., and Pöhlmann, S. (2020a). A multibasic cleavage site in the spike protein of SARS-CoV-2 is essential for infection of human lung cells. Mol. Cell *78*, 779–784.e5.

Hoffmann, M., Kleine-Weber, H., Schroeder, S., Krüger, N., Herrler, T., Erichsen, S., Schiergens, T.S., Herrler, G., Wu, N.H., Nitsche, A., et al. (2020b). SARS-CoV-2 cell entry depends on ACE2 and TMPRSS2 and is blocked by a clinically proven protease inhibitor. Cell *181*, 271–280.e8.

Iketani, S., Liu, L., Guo, Y., Liu, L., Chan, J.F.W., Huang, Y., Wang, M., Luo, Y., Yu, J., Chu, H., et al. (2022). Antibody evasion properties of SARS-CoV-2 Omicron sublineages. Nature *604*, 553–556.

Kaneko, Y., Kuwano, K., Kunitake, R., Kawasaki, M., Hagimoto, N., and Hara, N. (2000). B7-1, B7-2 and class II MHC molecules in idiopathic pulmonary fibrosis and bronchiolitis obliterans-organizing pneumonia. Eur. Respir. J. *15*, 49–55.

Kim, T.-K., Park, C.S., Jeoung, M.H., Lee, W.R., Go, N.K., Choi, J.R., Lee, T.S., Shim, H., and Lee, S. (2015). Generation of a human antibody that inhibits TSPAN8-mediated invasion of metastatic colorectal cancer cells. Biochem. Biophys. Res. Commun. *468*, 774–780.

Kummer, D., Steinbacher, T., Schwietzer, M.F., Thölmann, S., and Ebnet, K. (2020). Tetraspanins: integrating cell surface receptors to functional microdomains in homeostasis and disease. Med. Microbiol. Immunol. *209*, 397–405.

Lambrecht, B.N., and Hammad, H. (2010). The role of dendritic and epithelial cells as master regulators of allergic airway inflammation. Lancet *376*, 835–843.

Lamers, M.M., Beumer, J., van der Vaart, J., Knoops, K., Puschhof, J., Breugem, T.I., Ravelli, R.B.G., Paul van Schayck, J., Mykytyn, A.Z., Duimel, H.Q., et al. (2020). SARS-CoV-2 productively infects human gut enterocytes. Science *369*, 50–54.

Letuve, S., Sallon, C., Yang, X., Dumay, A., Bedja, S., Hamidi, F., Guillou, N., Mordant, P., Pretolani, M., and Taillé, C. (2019). Role of DNA Methylation in Muc5AC Hyperexpression in Severe Asthma (Eur Respiratory Soc).

López-Giraldo, A., Cruz, T., Molins, L., Guirao, Á., Saco, A., Cuerpo, S., Ramirez, J., Agustí, Á., and Faner, R. (2018). Characterization, localization and comparison of c-Kit+ lung cells in never smokers and smokers with and without COPD. BMC Pulm. Med. *18*, 123.

Martins, S.d.T., and Alves, L.R. (2020). Extracellular vesicles in viral infections: two sides of the same coin? Front. Cell. Infect. Microbiol. *10*, 593170.

Mason, R.J. (2020). Pathogenesis of COVID-19 from a cell biology perspective. Eur. Respir. J. *55*, 2000607.

Meng, B., Abdullahi, A., Ferreira, I.A.T.M., Goonawardane, N., Saito, A., Kimura, I., Yamasoba, D., Gerber, P.P., Fatihi, S., Rathore, S., et al. (2022). Altered TMPRSS2 usage by SARS-CoV-2 Omicron impacts infectivity and fusogenicity. Nature *603*, 706–714.

Mick, E., Kamm, J., Pisco, A.O., Ratnasiri, K., Babik, J.M., Castañeda, G., DeRisi, J.L., Detweiler, A.M., Hao, S.L., Kangelaris, K.N., et al. (2020). Upper airway gene expression reveals suppressed immune responses to SARS-CoV-2 compared with other respiratory viruses. Nat. Commun. *11*, 5854–5857.

Nazarenko, I., Rana, S., Baumann, A., McAlear, J., Hellwig, A., Trendelenburg, M., Lochnit, G., Preissner, K.T., and Zöller, M. (2010). Cell surface tetraspanin Tspan8 contributes to molecular pathways of exosome-induced endothelial cell activation. Cancer Res. *70*, 1668–1678.

Pastorino, R., Pezzullo, A.M., Villani, L., Causio, F.A., Axfors, C., Contopoulos-Ioannidis, D.G., Boccia, S., and Ioannidis, J.P.A. (2022). Change in age distribution of COVID-19 deaths with the introduction of COVID-19 vaccination. Environ. Res. *204*, 112342.

Planas, D., Saunders, N., Maes, P., Guivel-Benhassine, F., Planchais, C., Buchrieser, J., Bolland, W.-H., Porrot, F., Staropoli, I., Lemoine, F., et al. (2022). Considerable escape of SARS-CoV-2 Omicron to antibody neutralization. Nature *602*, 671–675.

Planas, D., Veyer, D., Baidaliuk, A., Staropoli, I., Guivel-Benhassine, F., Rajah, M.M., Planchais, C., Porrot, F., Robillard, N., Puech, J., et al. (2021). Reduced sensitivity of SARS-CoV-2 variant Delta to antibody neutralization. Nature *596*, 276–280.

Ravindra, N.G., Alfajaro, M.M., Gasque, V., Wei, J., Filler, R.B., Huston, N.C., Wan, H., Szigeti-Buck, K., Wang, B., Montgomery, R.R., et al. (2020). Single-cell longitudinal analysis of SARS-CoV-2 infection in human bronchial epithelial cells. Preprint at bioRxiv. https://doi.org/10.1101/2020.05.06.081695.

Robinot, R., Hubert, M., de Melo, G.D., Lazarini, F., Bruel, T., Smith, N., Levallois, S., Larrous, F., Fernandes, J., Gellenoncourt, S., et al. (2021). SARS-CoV-2 infection induces the dedifferentiation of multiciliated cells and impairs mucociliary clearance. Nat. Commun. *12*, 4354–4416.

Sachs, N., Papaspyropoulos, A., Zomer-van Ommen, D.D., Heo, I., Böttinger, L., Klay, D., Weeber, F., Huelsz-Prince, G., Iakobachvili, N., Amatngalim, G.D., et al. (2019). Long-term expanding human airway organoids for disease modeling. EMBO J. *38*, e100300.

Salahudeen, A.A., Choi, S.S., Rustagi, A., Zhu, J., van Unen, V., de la O, S.M., Flynn, R.A., Margalef-Català, M., Santos, A.J.M., Ju, J., et al. (2020). Progenitor identification and SARS-CoV-2 infection in human distal lung organoids. Nature *588*, 670–675.

Schleimer, R.P., Kato, A., Kern, R., Kuperman, D., and Avila, P.C. (2007). Epithelium: at the interface of innate and adaptive immune responses. J. Allergy Clin. Immunol. *120*, 1279–1284.

Schultze, J.L., and Aschenbrenner, A.C. (2021). COVID-19 and the human innate immune system. Cell *184*, 1671–1692.

Sette, A., and Crotty, S. (2021). Adaptive immunity to SARS-CoV-2 and COVID-19. Cell *184*, 861–880.

Shafiee, A., Moradi, L., Lim, M., and Brown, J. (2021). Coronavirus disease 2019: a tissue engineering and regenerative medicine perspective. Stem Cells Transl. Med. *10*, 27–38.

Sigal, A. (2022). Milder disease with Omicron: is it the virus or the pre-existing immunity? Nat. Rev. Immunol. *22*, 69–71.

Simon-Loriere, E., and Schwartz, O. (2022). Towards SARS-CoV-2 serotypes? Nat. Rev. Microbiol. *20*, 187–188.

Thorne, L.G., Bouhaddou, M., Reuschl, A.-K., Zuliani-Alvarez, L., Polacco, B., Pelin, A., Batra, J., Whelan, M.V.X., Hosmillo, M., Fossati, A., et al. (2022). Evolution of enhanced innate immune evasion by SARS-CoV-2. Nature *602*, 487–495.

Travaglini, K.J., Nabhan, A.N., Penland, L., Sinha, R., Gillich, A., Sit, R.V., Chang, S., Conley, S.D., Mori, Y., Seita, J., et al. (2020). A molecular cell atlas of the human lung from single-cell RNA sequencing. Nature *587*, 619–625.

VanBlargan, L.A., Errico, J.M., Halfmann, P.J., Zost, S.J., Crowe, J.E., Purcell, L.A., Kawaoka, Y., Corti, D., Fremont, D.H., and Diamond, M.S. (2022). An infectious SARS-CoV-2 B. 1.1. 529 Omicron virus escapes neutralization by therapeutic monoclonal antibodies. Nat. Med. *28*, 490–495.

Vieira Braga, F.A., Kar, G., Berg, M., Carpaij, O.A., Polanski, K., Simon, L.M., Brouwer, S., Gomes, T., Hesse, L., Jiang, J., et al. (2019). A cellular census of human lungs identifies novel cell states in health and in asthma. Nat. Med. *25*, 1153–1163.

Voglstaetter, M., Thomsen, A.R., Nouvel, J., Koch, A., Jank, P., Navarro, E.G., Gainey-Schleicher, T., Khanduri, R., Groß, A., Rossner, F., et al. (2019). Tspan8 is expressed in breast cancer and regulates E-cadherin/catenin signalling and metastasis accompanied by increased circulating extracellular vesicles. J. Pathol. *248*, 421–437.

Wang, R., Simoneau, C.R., Kulsuptrakul, J., Bouhaddou, M., Travisano, K.A., Hayashi, J.M., Carlson-Stevermer, J., Zengel, J.R., Richards, C.M., Fozouni, P., et al. (2021). Genetic screens identify host factors for SARS-CoV-2 and common cold coronaviruses. Cell *184*, 106–119.e14.

Xian, W., and McKeon, F. (2012). Adult stem cells underlying lung regeneration. Cell Cycle *11*, 887–894.

Yan, R., Zhang, Y., Li, Y., Xia, L., Guo, Y., and Zhou, Q. (2020). Structural basis for the recognition of SARS-CoV-2 by full-length human ACE2. Science *367*, 1444–1448.

Youk, J., Kim, T., Evans, K.V., Jeong, Y.I., Hur, Y., Hong, S.P., Kim, J.H., Yi, K., Kim, S.Y., Na, K.J., et al. (2020). Three-dimensional human alveolar stem cell culture models reveal infection response to SARS-CoV-2. Cell Stem Cell *27*, 905–919.e10.

Ziegler, C.G.K., Allon, S.J., Nyquist, S.K., Mbano, I.M., Miao, V.N., Tzouanas, C.N., Cao, Y., Yousif, A.S., Bals, J., Hauser, B.M., et al. (2020). SARS-CoV-2 receptor ACE2 is an interferon-stimulated gene in human airway epithelial cells and is detected in specific cell subsets across tissues. Cell *181*, 1016–1035.e19.

Stem Cell Reports, Volume *18*

Supplemental Information

SARS-CoV-2 infection of airway organoids reveals conserved use of Tetraspanin-8 by Ancestral, Delta, and Omicron variants

Lisiena Hysenaj, Samantha Little, Kayla Kulhanek, Melia Magnen, Kriti Bahl, Oghenekevwe M. Gbenedio, Morgan Prinz, Lauren Rodriguez, Christopher Andersen, Arjun Arkal Rao, Alan Shen, Jean-Christophe Lone, Leonard C. Lupin-Jimenez, Luke R. Bonser, Nina K. Serwas, Eran Mick, Mir M. Khalid, Taha Y. Taha, Renuka Kumar, Jack Z. Li, Vivianne W. Ding, Shotaro Matsumoto, Mazharul Maishan, Bharath Sreekumar, Camille Simoneau, Irina Nazarenko, Michael G. Tomlinson, Khajida Khan, Anne von Gottberg, Alex Sigal, Mark R. Looney, Gabriela K. Fragiadakis, David M. Jablons, Charles R. Langelier, Michael Matthay, Matthew Krummel, David J. Erle, Alexis J. Combes, Anita Sil, Melanie Ott, Johannes R. Kratz, and Jeroen P. Roose

SARS-CoV-2 infection of Airway Organoids Reveals Conserved Use of Tetraspanin-8 by Ancestral-, Delta-, and Omicron- Variants

Materials and Methods

KEY RESSOURCES TABLE

**Cell lines**	**Supplier**	**Catalogue**
HEK 293T ACE2	Integral Molecular	C-HA102
HEK 293T	ATCC	CRL-3216
Vero E6 cell lines	ATCC	61262

**Media component**	**Supplier**	**Catalogue number**
R-Spondin 1	Peprotech	120-38
FGF 7	Peprotech	100-19
FGF 10	Peprotech	100-26
Noggin	Peprotech	120-10C
A83-01	Tocris	2939
Y-27632	Abmole	Y-27632
SB202190	Sigma	S7067
B27 supplement	Gibco	17504-44
N-Acetylcysteine	Sigma	A9165-5g
Nicotinamide	Sigma	N0636
GlutaMax 100x	Invitrogen	12634-034
Hepes	Invitrogen	15630-056
Penicillin / Streptomycin	Invitrogen	15140-122
Primocin	Invivogen	Ant-pm-1
Advanced DMEM/F12	Invitrogen	12634-034
Liberase™ TL Research Grade	ThermoFischer	5401020001
EDTA	ThermoFischer	03690-100ML
Cyto-Fast™ Fix/Perm Buffer Set	ThermoFischer	426803

BSA	ThermoFischer	A7030-10G
PBS without calcium, magnesium	UCSF	
Dead Cell Removal Kit		130090101

**Antibodies**	**Supplier**	**catalog #**
TSPAN8 Alexa Fluor 405	R&D systems	FAB4734V-100UG
CD66c Briliant Violet 510	BD	742684
CD86 Briliant Violet 650	BioLegend	305427
EPCAM Briliant Violet 711	BioLegend	324239
ckit (CD117) Briliant Violet 785	BioLegend	313237
CD49f PE	BioLegend	313612
CD80 PE-Cy5	bioLegend	305209
CD271 PE-Cy7	bioLegend	345109
ACE2 Alexa Fluor	R&D systems	FAB933R-100UG
CEACAM5	R&D systems	FAB41281S-100UG
Zombie NIR	Biolegend	--
TrueStain	Innovex Biosciences Inc.	NB309-5S
TUBA	Sigma-Aldrich	sc-23950 AF488
MUC5AC Biotin	Thermo Fischer Scientific	MA5-12175
TMPRSS2	Abcam	ab242384
dsRNA	Scicons	

**Software**		
GraphPad Prism 9		
Fiji/ImageJ (V) software 2.1.0/1.53c		
FlowJO Software V10.1		
Seurat		

EXPERIMENTAL MODEL AND SUBJECT DETAILS

Patient samples

Normal adjacent lung tissue was collected from non-small cell lung cancer patients; 2425LL, 2450UL, 2477UL, 2478UL, 2520LL, 2521UL, 2522UL, 2524UL, 2525UL, 2526UL, 2527UL, 2531UL, 2547UL, 2551ML. Samples were collected under UCSF study CC#00654 (IRB 11-06107) from patients undergoing thoracic surgery.

Donor lungs rejected for transplantation were received from organ procurement organization (Donor Network West) as previously described (Ross et al., 2019); samples L1, L2, L3, L5, L6, and L7. Explicit approval for the use of donor lungs for research was sought from each donor’s family by Donor Network West as part of the standard organ donation process. Local institutional review board approval is not required because research on tissues from deceased organ donors is not considered human subject research, although institutional biosafety approval was obtained from University of California, San Francisco (UCSF) Institutional Review Board.

METHOD DETAILS

Viruses and Cell Lines

Vero E6 cells were cultured in Dulbecco's Modified Eagle Medium (UCSF Media Production) supplemented with 10% fetal bovine serum (Corning), penicillin/streptomycin (UCSF Media Production) and L-glutamine (Corning) in a humidified incubator at 37°C and 5% CO2. SARS-CoV-2 virus (USA-WA1/2020 strain) was propagated in Vero E6 cells. The Vero E6 cells were infected with the SARS-CoV-2 virus (Wuhan-Hu-1; GenBank accession number MN908947.3, Delta variant B.1.617.2, GenBank: OW998779.1, Omicron BA.1/2021, complete genome GenBank: OP090659.1), incubated at 37°C, 5% CO2 and after 72h the supernatant was collected. The virus was aliquoted and stored at -80 °C. This work was done under Biosafety Level 3 (BSL-3) conditions.

Determination of Virus Titers using Plaque Assay

Viral titer was quantified using a plaque assay in Vero E6
cells. 10-fold dilutions of the virus stock were added to Vero E6 cells in a 12-well plate for 1 hour, after which an overlay of 1.25% Avicel RC-591 in DMEM (UCSF Media Production) was added. The cells were incubated at 37°C, 5% CO2 for 72 hours. The cells were fixed with 10% formalin, stained with crystal violet, and washed with water. The plaques were counted to determine the titer of the virus stock. All work was done under BSL-3 conditions.

H1N1/PR8 1 (gift from Dr. Yoshihiro Kawaoka) was propagated in serum pathogen-free fertilized chicken eggs (Charles River) as previously described 2. In brief, freshly fertilized eggs were kept in an automatic egg turner for 10 days before injection of the virus into the allantoic cavity. Embryos were incubated with the virus for 2 days. Allantoic fluid was harvested and snap-frozen in liquid nitrogen. Titers were determined with a hemagglutination assay. This work was done under BSL-2 conditions.

Airway Organoid Cultures

Organoids were generated from non-tumor lung tissue obtained from patients undergoing lung resection or from donor organs, using a protocol adapted from Sachs and colleagues (Sachs et al., 2019). The Roose lab has IRB Exempt certification (IRB #: 12-09467). Airway tissue was washed 2X with DPBS, placed in sterile petri dishes (Corning® Gosselin™) and filled with 10 ml of digestionbuffer (DMEM/F12, Collagenase I 1.5mg/ml (Thermo Fischer Scientific, cat#17100017), HEPES (UCSF cell culture facility), penicillin 10,000 IU/mL (Thermo Fischer Scientific
Cat#15140122). The tissue chunk was minced in 1-3mm pieces using a scalpel blade and transferred with the digestion buffer into a 50 ml Falcon tube. After 1 hour of incubation at 37°C with shaking at 125 rpm, the digested tissue was filtered through a 100μM filter and transferred into a 50 ml Falcon tube. Cells were pelleted (600g, 5 minutes at 4°C) and washed
twice with DPBS (UCSF Cell Culture Facility). Airway cell pellet was then resuspended in 1300 μL Matrigel (Corning Cat#356230) and plated in ~50 μL droplets in a 24 well tissue culture plate. Plates were placed at 37°C with 5% CO2 for 20 min to solidify the Matrigel droplets upon which 550μL of airway organoid media was added to each well.

Plates were incubated in the standard tissue culture incubator at 37°C. Images were taken on Days 10 to 21 using a BZX700 inverted microscope with a CCD cooling camera and BZ-X analysis software (KEYENCE).

Airway Organoid Biobank

After 1 to 3 weeks of growth, organoids were dissociated into single cells using TrypLE express. Cells were washed twice with DPBS, pelleted and resuspended in freezing media (Gibco™ Recovery™ Cell Culture Freezing Medium -1560446) and transferred immediately in the Mr. Frosty™ Freezing Container (Cat 51100-001) in the -80C Freezer. Frozen vials were then transferred to the Liquid Nitrogen freezer. When needed, cells were quickly thawed using the 37°C water bath, and transferred to 15ml tubes filled with DPBS. Cells were pelleted (600g, 5 minutes at 4°C) and washed twice with DPBS. Airway cell pellets were then resuspended in 1300 µL Matrigel (Corning) and plated in ~50 µL droplets in a 24 well tissue culture plate. Plates were placed at 37°C with 5% CO2 for 20 min to solidify the Matrigel droplets upon which 550µL of airway organoid media was added to each well. Plates were incubated in a standard tissue culture incubator at 37°C.

TSPAN8 and CD9 plasmid

Plasmids were generated by the Core Facility "Signaling Factory" of the University of Freiburg. CD9 and mCherry sequences were inserted into the pcDNA3.1 Hygro (+) plasmid digested using XhoI. The direction on insert was controlled by sequencing. To generate Tspan8-Cherry, pcDNA3.1 containing hTspan8 and pcDNA3.1-CD9-Cherry were digested using BshTI and NotI and Tspan8 insert was ligated with the Cherry-containing plasmid. Digestion and sequencing analysis were used to select colonies containing correct fusion constructs.

HEK cell lines generation

HEK293T and ACE2+ HEK293Twere transfected with 1ug of either an empty mCherry backbone, TSPAN8-mCherry or CD9 mCherry (provided by Irina Nazarenko) and 1ug of pEF/myc-his B (Addgene) to confer blasticidin (BSD) resistance. Cells were transfected using Fugene (Promega E2691) in Opti-MEM according to the manufacturer's instructions. Following transfection, cells were cultured with BSD

(4ug/ml for HEK293T cells and 8µl/ml for ACE2+ HEK293T cells). Stable clones were sorted for mCherry+ cells and replated in culture media with the appropriate amount of BSD.

SARS-CoV-2 Delta and Omicron Generation

Blood samples were obtained after written informed consent from adults with PCR-confirmed SARS-CoV-2 infection who were enrolled in a prospective cohort study approved by the Biomedical Research Ethics Committee at the University of KwaZulu–Natal (reference BREC/00001275/2020).

BA.1 and BA.1.1 isolate: The Omicron/BA.1 was isolated from a residual swab sample with SARS-CoV-2 isolation from the sample approved by the University of the Witwatersrand Human Research Ethics Committee (HREC) (ref. M210752) as described in Cele *et al* (Cele et al., 2022).

Pseudotyped virus generation

293T cells were transfected with plasmid DNA (per 15cm2 plate: 3.4µg ng of spike mutants, 10 µg pspAX2), and 10 µg Luciferase reporter). Cell culture media was replaced after 16-24hr, then added fresh complete media (DMEM, 10% Serum, P/S, L-Glut). Supernatant containing pseudovirus particles was collected at 48hr after transfection, filtered (0.45 µm). The collected pseudovirus was concentrated on 20% sucrose cushion spinning at 24000RPM for 1hr at 4°C using SW28 rotor. The concentrated was reconstituted in complete DMEM media (5X times concentrated) quantified with a p24 assay (Takara #632200).

Airway Organoid Infection

Airway organoids were removed from Matrigel following 1 minute incubation with Dispase 0.5U/ml (StemCell Technologies, Cat #07913), carefully transferred into a 15 ml tube, washed with DPBS (UCSF Cell Culture Facility) supplemented with 5 mm EDTA (Cellgro, Cat#46-034-CI) and pelleted (200g, 3 minutes at Room Temperature). Organoid pellets were resuspended in 500 µl of airway organoid media. Virus was added at MOI=0.15 for H1N1/PR8 mCherry and MOI=0.3 for SARS-CoV-2. Every organoid well contained 10,000 cells. For mock infections, organoids go through the same process and handling without exposure to viral particles. After 2 hours of incubation, airway organoids were washed twice with PBS and resuspended in 300µL

Matrigel (Corning) and plated in ~50 µL droplets in a 24 well tissue culture plate. Plates were placed at 37°C with 5% CO_2 for 20 min to solidify the Matrigel droplets upon which 550µL of airway organoid media was added to each well. Plates were incubated in the standard tissue culture incubator at 37°C for 92 hours. After 72 hours spectral flow cytometry analyses (CYTEK Aurora) and Confocal Analyses (Zeiss, SP8) were performed.

TSPAN8- and ACE2- blocking antibody assays

Airway organoids were removed from Matrigel following a 1-minute incubation with Dispase 0.5U/ml (StemCell Technologies, Cat #07913), carefully transferred into a 15ml tube, washed with DPBS (UCSF Cell Culture Facility) supplemented with 5 mm EDTA (Cellgro, Cat#46-034-CI) and pelleted (200g, 3 minutes at Room Temperature). Organoid pellets were resuspended in 500 µl of airway organoid media with isotype control RatgG2B Isotype control (R&D Systems, Cat#MAB0061), humanTSPAN8 blocking antibody (R&D Systems, #MAB4734-SP) and/or human ACE2 (R&D Systems, #AF933-SP) at 50µg/ml. After 1 hour incubation with the antibody, SARS-CoV-2 was added. After 2 hours of incubation with the virus, airway organoids were washed twice with PBS and resuspended in 300µL Matrigel (Corning) containing human TSPAN8 blocking antibody (R&D Systems, #MAB4734-SP) and/or human ACE2 (R&D Systems, #AF933-SP) at 50µg/ml. and plated in ~50 µL droplets in a 24 well tissue culture plate. Plates were placed at 37°C with 5% CO_2 for 20 min to solidify the Matrigel droplets upon which 550µL of airway organoid media was added to each well. Plates were incubated in the standard tissue culture incubator at 37°C for 24 to 72 hours. Spectral flow cytometry analyses (CYTEK Aurora) and Confocal Analyses (Zeiss, SP8) were performed.

Spectral Flow analysis

Organoids were dissociated into single cells using TrypLE express (ThermoFisher Scientific Cat#12604012). Single Cell suspensions were transferred to 2 ml cryovial tubes and washed twice with FACS buffer (2% FBS, 0,5% BSA, DPBS, 0.1mg Liberase + Y-27632 5mM (Abmole) before incubating for 10 minutes with Ig block (True-Stain Monocyte Blocker™ Innovex Bioscience). Cells were incubated next with Zoombie NiR (Biolegend) and the antibody mix for 35 minutes followed by two washes

with FACS Buffer. Cells were fixed for 30 minutes with Permeabilization Fixation Buffer (eBioscience) washed twice and for 45 minutes with antibodies staining for intracellular targets followed by a 20-minute incubation with secondary conjugated antibodies. Cells were washed with DPBS (UCSF facilities) before acquiring in the CYTEK.

Antibodies used for spectral flow cytometry: TSPAN8 AF405, clone 45811(R&D systems), CD66c (CEACAM6) BV510, clone B6.2 (BD Bioscience), EPCAM BV711, clone 9C4, Biolegend; CD86 BV650, clone IT2.2 (BioLegend), CD117 BV785, clone 104D2, BioLegend, CD49f PE, clone GoH3 (BioLegend), CD80 PE-Cy5, clone 2D10 (BioLegend), CD271 PE-Cy7, clone ME20.4, BioLegend, ACE2 AF647, clone Q9BYF1 (R&D systems), CEACAM5 APC-Fire750, clone 487609 (R&D systems), MUC5AC Biotin, clone 45M1 (ThermoFischer Scientific), Streptavidine APC (eBioscience), anti-TMPRSS2, clone EPR3862 (Abcam), acetylated Tubulin AF488, clone 6-11B-1 (SantaCruz Biotechnologies), anti-dsRNA (Scicons), IGg BV605, clone4053 (Biolegend).

Confocal Imaging

Airway organoids were removed from Matrigel following 1 minute incubation with Dispase 0.5U/ml (StemCell Technologies, Cat #07913), carefully transferred into a 15 ml tube, washed with DPBS (UCSF Facility) and pelleted (200g, 2 minutes at Room Temperature). The organoid pellet was incubated for 30 minutes in 4% PFA 10% FBS Triton 0.1X and TrueStain. Organoids were washed twice with PBS-Triton 0.1X and stained overnight at 4°C with primary antibodies dsRNA Ab (clone J2, Scicons), SARS-CoV-2 Nucleocapsid Ab (Novus Biologicals, Cat#NB100-56576SS). Organoids were then washed and incubated for two hours with secondary antibodies Goat anti mouse IGg AF555, clone4053 (Biolegend), CD49f PE, clone GoH3 (BioLegend), ACE2 AF647, clone Q9BYF1 (R&D systems), and acetylated Tubulin AF488, clone 6-11B-1 (SantaCruz Biotechnologies). After staining, organoids were washed with DPBS- 0.1X Triton and resuspended in Fructose-Glycerol Clearing Solution 60% (vol/vol) glycerol and 2.5 M fructose. Organoids were mounted on coverslips and imaged with Leica SP8 Confocal Microscopy. Confocal Z-stack images were generated using the staining maxima. For the images in the figure panels, Z-stacks with 0.5-μm separation were acquired and all Z-stacks were combined to a z-stack

projection throughout the entire organoid imaged. Alternatively, to highlight detail such as staining of ciliated cells by means of acTUBA, only 3 Z-stacks with most in-focus acTUBA staining were combined to create a z-projections with just 3 z-stacks per image.

Single cell and library preparation for scRNA-sequencing

For the single-cell RNA sequencing experiments each organoid was generated from 4 different donors. Airway organoids were removed from Matrigel following 1 minute incubation with Dispase 0.5U/ml (StemCell Technologies, Cat #07913), carefully transferred into a 15 ml tube, washed with DPBS (UCSF Cell Culture Facility) supplemented with 5 mm EDTA (Cellgro, Cat#46-034-CI) and pelleted (200g, 3 minutes at Room Temperature). Organoid pellets were resuspended in 500 µl of airway organoid media. Virus was added at MOI=0.3 for SARS-CoV-2 and the same amount of DMEM was added in the Mock condition. Every organoid well contained 10,000 cells. The experiments was performed in triplicates for each condition. After 2 hours of incubation, airway organoids were washed twice with PBS and resuspended in 300µL Matrigel (Corning) and plated in ~50 µL droplets in a 24 well tissue culture plate. Plates were placed at 37°C with 5% CO2 for 20 min to solidify the Matrigel droplets upon which 550µL of airway organoid media was added to each well. Plates were incubated in the standard tissue culture incubator at 37°C for 72 hours. After 72 hours organoids were dissociation into single-cell suspension using TrypLE express (ThermoFisher Scientific Cat#12604012), cells were counted, and the same conditions were pooled together (78,000 total cells). Pooled cells were then loaded evenly across two lanes in the Chromium Controller for generating single-cell contained in lipid droplets following the manufacturer's instructions (10X Genomics). A Chromium Single cell 3' Reagent Kit (v3.1) (10X Genomics) was used for reverse transcription, cDNA amplification and library construction of the gene expression libraries following the manufacturer's instructions. All samples were encapsulated, and cDNA was generated within 6 hours after organoid processing. Finally, Pooled libraries were sequenced on an Illumina NovaSeq 6000

QUANTIFICATIONS AND STATISTICAL ANALYSES

Single cell RNAseq analysis

Data pre-processing of 10x Genomics Chromium scRNA-seq data:

Data pre-processing was performed as previously described[16]. Briefly, sequencer-obtained bcl files were demultiplexed into individual samples using the Cellranger (v.3.0.2, 10X Genomics) suite of tools (https://support.10xgenomics.com). Feature-barcode matrices were obtained for each sample by aligning the raw fastqs to GRCh38 reference genome (annotated with Ensembl v85) using the Cellranger count. Raw feature-barcode matrices were loaded into Seurat (v.4.0.3)[17] and genes with fewer than 3 UMIs were dropped from the analyses. Matrices were further filtered to remove events with greater than 30% percent mitochondrial content, events with greater than 50% ribosomal content, or events with fewer than 250 total genes. The cell cycle state of each cell was assessed using a published set of genes associated with various stages of human mitosis.

Inter-sample doublet detection:

Inter-sample doublet detection was performed as previously described[16]. Libraries containing samples pooled prior to loading were processed using Freemuxlet (https://github.com/statgen/popscle) to identify clusters of cells belonging to the same patient via SNP concordance. Cells are classified as singlets arising from a single library, doublets arising from two or more libraries, or as ambiguous cells that cannot be accurately assigned to any existing cluster (due to a lack of sufficient genetic information).

Data quality control and Normalization:

The filtered count matrices were normalized, and variance stabilized using negative binomial regression via the scTransform method offered by Seurat[17]. The effects of mitochondrial content, ribosomal content, and cell cycle state were regressed out of the normalized data to prevent any confounding signal. The scTransformed data from different sequencing libraries were combined and normalized using Harmony integration software[15]. The merged object, after final QC, contained 21,225 single cells.

Intra-sample heterotypic doublet detection:

All libraries were further processed to identify heterotypic doublets arising from the 10X sample loading. Processed, annotated Seurat objects were processed using the DoubletFinder package[12]. Briefly, the cells from the object are modified to generate artificial duplicates, and true doublets in the dataset are identified based on similarity to the artificial doublets in the modified gene space. The prior doublet rate per library was approximated using the information provided in the 10x knowledgebase (https://kb.10xgenomics.com/hc/en-us/articles/360001378811) and this was corrected to account for homotypic doublets using the per-cluster numbers in each dataset.

Differential expression tests and cluster marker genes, cluster annotation:

Differential gene expression (DGE) tests were performed on log-normalized gene counts using the Poisson test (with a latent batch variable to account for multiple library preparations) as implemented in the FindMarkers/FindAllMarkers functions in Seurat. Cluster marker gene lists were generated by applying the Poisson test to identify upregulated genes for one cluster against all other clusters in the dataset. The resulting top ranked genes (sorted by log-fold change) for each cluster and canonical markers[13] were used for cluster annotation (Fig. 3, Supp. Fig. S6).

Data Mining

To examine presence of *ACE2* and *TSPAN8* co-expressing cells in vivo in normal human airway, we analyzed the processed 10X single-cell sequencing data from Travaglini et al(Travaglini et al., 2020)[3]. Of the 60,993 cells derived from airway tissue of 3 patient donors in this dataset, 48 cells were found with at least 1 UMI (unique molecular identifier) for both genes. Only 1 cell derived from patient 1, which had fewer cells sequenced overall and so we excluded it. The expression values represent ln(UMI-per-10K + 1) in each of the 47 cells from patients 2 and 3. Cell type designations were determined by Travaglini *et al*. Differential expression of TSPAN8 in nasal swabs of adult patients with acute respiratory illness (ARI) due to COVID-19 (n=93) or other viral infection (n=41), in comparison to patients with ARI due to non-viral etiology (n=100), was derived from Mick et al(Mick et al., 2020). The differential expression analysis between the 3 viral status groups was performed with the R package limma while controlling for gender and age.

Statistics

Statistical analyses were run in R (version 4.0.2) (quote 1). Paired Samples Wilcoxon Test were performed using Wilcox.Test (stat v4.0.2) (quote 1). Outliers were removed if the value was over Q3+1.5 IQR. We used PCA (FactoMineR v2.4) to perform Principal Component Analysis and fviz_pca_ind or fviz_pca_var (factoextra v1.0.7) (quote 3) for visualization. Community distances were evaluated based on relative abundance of cell populations by PERMANOVA (Bray, *p<0.05). For spearman's and pearson's correlations, we used cor function (stat v4.0.2) to compute the correlation coefficients and lm (stat v4.0.2) to fit linear models. Data management was done using tidyverse (v1.3.0) (quote 4). All graphs were built using ggplot2 (quote 5) (Kassambara and Mundt, 2017; Lê et al., 2008; Team, 2013; Wickham et al., 2016; Wickham et al.). UMAP dimensionality reduction, discovery of upregulated/downregulated genes, and gene expression related plots were constructed in R v 4.0.3 (1) via Seurat v 4.0 provided by the Satija Lab (Hao et al., 2020) and ggplot2 v 3.3.3 Clusters were separated using the Louvain clustering method with a resolution of 0.6, and upregulated differential expression gene scores between clusters were used to establish cell type identities(Wickham et al., 2016).

Bar graph of enrichment analysis up regulated pathway and processes based in cells positive for SARS-CoV-2 reads were generated using Metascape(Zhou et al., 2019).

scRNA-seq specific Statistical Analysis:

All statistical analyses were performed using R. Dot plots were generated using Seurat's 'DotPlot' function. Differentially expressed genes were identified using the Poisson test (with a latent batch variable to account for multiple library preparations) as implemented in the FindMarkers/FindAllMarkers functions in Seurat.

Software

GraphPad Prism 9 was used for DATA visualization. Fiji/ImageJ (V) software 2.1.0/1.53c was used for confocal microscopy analysis. FlowJO and CYTEK Aurora software 10.7.1 was used for spectral flow analyses.

References

Cele, S., Jackson, L., Khoury, D.S., Khan, K., Moyo-Gwete, T., Tegally, H., San, J.E., Cromer, D., Scheepers, C., and Amoako, D.G. (2022). Omicron extensively but incompletely escapes Pfizer BNT162b2 neutralization. Nature *602*, 654-656.

Hao, Y., Hao, S., Andersen-Nissen, E., Mauck, W.M., Zheng, S., Butler, A., Lee, M.J., Wilk, A.J., Darby, C., and Zagar, M. (2020). Integrated analysis of multimodal single-cell data. bioRxiv.

Kassambara, A., and Mundt, F. (2017). Package 'factoextra'. Extract and visualize the results of multivariate data analyses *76*.

Lê, S., Josse, J., and Mazet, F. (2008). Package 'FactoMineR. J Stat Softw [Internet] *25*, 1-18.

Mick, E., Kamm, J., Pisco, A.O., Ratnasiri, K., Babik, J.M., Castañeda, G., DeRisi, J.L., Detweiler, A.M., Hao, S.L., and Kangelaris, K.N. (2020). Upper airway gene expression reveals suppressed immune responses to SARS-CoV-2 compared with other respiratory viruses. Nature communications *11*, 1-7.

Ross, J.T., Nesseler, N., Lee, J.W., Ware, L.B., and Matthay, M.A. (2019). The ex vivo human lung: research value for translational science. JCI Insight *4*.

Sachs, N., Papaspyropoulos, A., Zomer-van Ommen, D.D., Heo, I., Bottinger, L., Klay, D., Weeber, F., Huelsz-Prince, G., Iakobachvili, N., Amatngalim, G.D.*, et al.* (2019). Long-term expanding human airway organoids for disease modeling. EMBO J *38*.

Team, R.C. (2013). R: A language and environment for statistical computing.

Travaglini, K.J., Nabhan, A.N., Penland, L., Sinha, R., Gillich, A., Sit, R.V., Chang, S., Conley, S.D., Mori, Y., and Seita, J. (2020). A molecular cell atlas of the human lung from single-cell RNA sequencing. Nature *587*, 619-625.

Wickham, H., Chang, W., Henry, L., Pedersen, T., Takahashi, K., Wilke, C., Woo, K., Yutani, H., and Dunnington, D. (2016). Springer-Verlag. New York.

Wickham, H., Francois, R., Henry, L., and Müller, K. others. 2015.". Dplyr: A Grammar of Data Manipulation" R Package Version 04 *3*.

Zhou, Y., Zhou, B., Pache, L., Chang, M., Khodabakhshi, A.H., Tanaseichuk, O., Benner, C., and Chanda, S.K. (2019). Metascape provides a biologist-oriented resource for the analysis of systems-level datasets. Nature communications *10*, 1-10.

Supplementary Figures Legends

Supplementary Figure 1: Donor-derived airway organoids display distinct but stable composition.

A) Brightfield images of organoids derived from the upper and lower region of the left airway of individual L2. Scale bars=200µm. Pie charts representing the distribution of cell populations found in airway organoids from the upper and lower lobes of subjects L2 and L7. B) Pie charts representing the distribution of cell populations in airway organoids from different donors analyzed by Spectral flow of different passages. In A and B, each section of the pie chart represents the cell population mean from 3 independent experiments with at least 3 replicates for the distinct donor-derived organoids. C) Table of 20 biobanked airway organoids from 20 different subjects. Indicated are the resource qualities of this biobank with indicated cryovial amount and passage number. Normal adjacent lung tissue was collected from non-small cell lung cancer patients; 2425LL, 2450UL, 2477UL, 2478UL, 2520LL, 2521UL, 2522UL, 2524UL, 2525UL, 2526UL, 2527UL, 2531UL, 2547UL, 2551ML. Alternatively, lung samples were obtained from donor lungs rejected for or left over from transplantation, received from organ procurement organization (Donor Network West) as previously described (Ross et al., 2019); samples L1, L2, L3, L5, L6, and L7.

D) TMPRSS2+ cells fraction in airway organoids based on spectral flow cytometry analyses. Bars represent mean, error bars are SEM. 3 independent experiments with at least 3 replicates for the distinct donor-derived organoids were performed. One way non-parametric ANOVA was performed to compare if there is differences between individual organoids.E) ACE2+ cells fraction in airway organoids based on spectral flow cytometry analyses. Bars represent mean, error bars are SEM. Experiments were repeated 3-5 times with at least 3 replicates each. One way non-parametric ANOVA was performed to compare if there is differences between individual organoids.

F) Spectral flow cytometry gating strategy. Cell populations are defined after excluding ZombieNIR-positive, dead cells and doublets in steps a-c, using the indicated gating strategies. (1) acTUBA + CD271- are considered ciliated cells. Fraction (d) is analyzed for MUC5AS and TSPAN8 and (2) MUC5AC+ acTUBA- cells are considered goblet-like cells. (3) TSPAN8+ MUC5AC- acTUBA- are defined as pre-goblet cells. Fraction (e) is analyzed for cKit and (g) are considered cKit-positive cells. Fraction (f) is analyzed for CD49f and CD271, (4) CD49f+ CD271-, (5) CD49f+ CD271+ and (6)

CD49f- CD271+ are considered basal stem cells and (7) CD49f- CD271- are undefined cells. G) Spectral flow cytometry gating strategy for mCherry+ cell populations. The same gates shown in Supplementary 1 A) were drawn on live, single cell and mCherry+ cells (h).

Supplementary Figure 2. SARS-CoV-2 infection in airway organoids.

A) Pie charts representing distribution of cell populations in organoids Mock, infected with SARS-CoV-2 -WA-1, at 72h p.i. (MOI=0.3) for 5 independent experiments. The pie chart fraction represents the mean of the cell populations for every independent experiment. B) Spectral flow analyses of the percentage of cells positive for CEACAM6, CEACAM5, CD80, CD86 and ACE2 at 72h p.i. (MOI=0.3) in live cells of Mock and SARS-CoV-2 WA-1 infected organoids. For SARS-CoV-2 infected organoids, the fraction of ACE2+ cells (or other marker-positive cells) is shown in non-infected cells (dsRNA-) or infected cells (dsRNA+) condition. Each dot represents the mean value of every experiment. Bars represent mean, error bars are SEM. Paired t-test, *p < 0.05, ****p<0.0001; ns, non-significant.

C) Pie charts representing the distribution of each cell population in Mock and SARS-CoV-2-WA-1 infected organoids at 72h p.i. (MOI=0.3). Each fraction of the pie chart represents the mean value of 3 independent experiments of the specific cell population in distinct donor-derived airway organoids. D-F) Box and whisker plots representing the spectral flow analyses of the % of (D) goblet-like cells (Muc5AC+), (E)CD49f- CD271- , (F) CD49f+ or CD271+in Mock and SARS-CoV-2-WA-1 infected organoids. Each dot represents the mean of % cell type for 3 independent experiments with 3-5 replicates per experiment for the distinct donor. Wilcoxon signed-rank paired test,*p<0.05. G) Quantification of percentage relative decrease in cell yield post-infection as a comparison to the cell number cell prior to the infection; for Mock and infected organoids at 72h post H1N1/PR8 infection (MOI=0.15). H1N1/PR8 virus did not induce increased levels of cell death, compared to mock infection Bars represent mean, and error bars are SEM, n=3 to n=5. Paired t-test, *p＜0.05; ns, non-significant. in mock and SARS-CoV-2-WA-1 infected organoids (MOI=0.3, 72h p.i.). Each dot represents the mean value of the percentage of the cell population in distinct donor-derived airway organoids for every independent experiment. 3 independent experiments with at least 3 replicates for the distinct donor-derived organoids were

performed.

Supplementary Figure 3. Donor-derived airway organoid responses to SARS-CoV-2.

A) Principal component analysis (PCA) using cell populations and infection conditions (all SARS-CoV-2-WA-1). PCA reduced the descriptors into two dimensions as shown in the individual plot. Each point corresponds to an observation in the dataset and were colored according to their mock (blue) or infection (red) state. Community distances were evaluated based on relative abundance of cell populations by PERMANOVA (Bray, $*p<0.05$). The PCA showed that 2525UL components change very minor to SARS-CoV-2 infection, while 2450UL and 2522UL reveal robust component alterations to SARS-CoV-2 infection. 3 independent experiments with at least 3 replicates for the distinct donor-derived organoids were performed. B-G) Scatter Plot showing the relationship between SARS-CoV-2-WA-1 infection rate and fraction B) CD86+ cells, C) cKit+ cells, D) MUC5AC+ cells, E) acTUBA+ cells prior to infection, F) Age of or (G) ACE2 the adult stem cell donor. Each dot represents the mean of % cell type in 3 independent experiments with at least 3 replicates for the distinct donor-derived organoid. Linear regression, R2 value and Pearson Correlation statistical significance are stated on the graph. Every dot shows the mean of the fraction of the specific cell in 3 independent experiments with 3-5 replicates each. Functions of the positive or negative correlations are depicted by the Linear Model with R2 as value to indicate how well the linear model function agrees with the individual data points. If R2=0 then 0% of the data points follow the linear model, if R2=0.5 then 50% of the data points follow the linear model, and if R2=1 then 100% of the data points follow the linear model. H-L) Box and whisker plots representing the percentage of (G) CD86+ cells, (I) CD80+ cells, (K) CEACAM5+ and (L) CEACAM6+cells analyzed by spectral flow cytometry for 12 Mock and SARS-CoV-2-WA-1 infected organoids. Each dot represents the mean of % cell type for the distinct donor. 3 independent experiments with at least 3 replicates for the distinct donor-derived organoids were performed. Wilcoxon signed-rank test, $*p<0.05$, N=12, n=3. Outliers were removed if the value was over Q3+1.5 IQR.

Supplementary Figure 4: scRNAseq analyses in airway organoids

A) Dot plot of canonical and top differentially expressed genes used to determine cluster annotations established in Fig 5A. B) Cells negative for SARS-CoV-2 reads (orange) and positive for SARS-CoV-2 reads (blue) overlay on the UMAP from Fig 5A. A sample of 100 randomly selected cells each for SARS-CoV-2 negative (orange) and SARS-CoV-2 positive (blue) populations selected for subsequent DGE analysis. Image is representative of independent random samples.

C) Cells positive for *TSPAN8, ACE2, TMPRSS2, NRP1, FURIN, CSTL1, CD24, AREG, CD9, CD37*, nCountSARS-CoV-2 reads and log (base 2) SARS-CoV-2 read counts (orange) overlaid on the UMAP seen in Fig 5A. Neutralizing antibodies from COVID-19 patients have multiple targets (Brouwer et al., 2020; Chi et al., 2020), suggesting that protective immune responses occur that may block interactions of molecules other than the S protein-ACE2 receptor pair. In our scRNA data *RALA* and *CD24* reads were present in infected cells. RALA (RAS like Proto-Oncogene A) was identified in the host-coronavirus protein network (Gordon et al., 2020) and infection levels of a hepatoma cell line is reduced when deleted by CRISPR(Wang et al., 2021a). CD24 is a glycosyl-phosphatidyl-inositol (GPI)-anchored membrane protein that can repress the host response to DAMPS (Chen et al., 2009) and EXO-CD24 exosomes are explored in clinical trials as therapy for COVID19 patients (Clinical Trial NCT047477574). Studies using cell lines had also implicated additional mediators of infection, including AXL (Wang et al., 2021b) CD147 (Shilts et al., 2021; Wang et al., 2020) and neuropilin-1 (NRP1) (Cantuti-Castelvetri et al., 2020; Daly et al., 2020).

Top differentially upregulated genes, including *TSPAN8* (arrow), within representative random subsample of SARS-CoV-2 positive (top) versus SARS-CoV-2 negative (bottom) cells. Circle sizes indicate the percentage of cells within the total cell population that the specific gene is expressed in. We did not observe CD9 or any other Tetraspanin enriched in infected cells (See Supplemental Tables).

Supplementary Figure 5: TSPAN8 correlates with infection rate in airway organoids.

A) Box and whisker plots representing the percentage of TSPAN8+ cells analyzed by spectral flow cytometry for each distinct donor in Mock and H1N1/PR8 infected organoids (MOI=0.15, 72h p.i.). Every dot represents the mean of cell percentage for 3 independent experiment in that distinct donor.

B) Spectral flow cytometry plots of overlay between dsRNA- cells (in gray) and

dsRNA+ cells (in red) in the most infected SARS-CoV-2-WA-1 infected organoids at 72h p.i. (MOI=0.3). X axis represents TSPAN8 expression intensity and Y axis represents ACE2 expression intensity.

C) Scatter Plot showing the relationship between SARS-CoV-2-WA-1 infection rate and ACE2+ TSPAN8+ cells prior to infection, prior to infection. Each dot represents the mean of 3 independent experiment for the distinct donor. Linear regression, R2 value and Pearson Correlation statistical significance stated on graph. Mean values, N= 3, n=3 for every donor-derived organoid.

Supplementary Figure 6: TSPAN8 is a facilitator of SARS-CoV-2

A) Spectral Flow histogram plot of ACE2 (left) and mCherry (right) intensity in ACE2 mCherry, ACE2 CD9 and ACE2 TSPAN8 HEK293T cells.

B) Median Fluorescence Intensity (MFI) of ACE2 in ACE2 mCherry, ACE2 CD9 and ACE2 TSPAN8 HEK293T cells measured by Spectral flow.

C) Scheme of pseudo viruses generated and utilized.

D) Luminescence measured as a function of pseudovirus entry for the backbone Ps. PsVirus expressing spike of WA1 (D614G)-, Delta VOC, and Omicron VOC as well as VSV-G were tested at 48hours post Psvirus infection.

E) Mutations found on the Spike protein of Delta and Omicron Strain of SARS-CoV-2 (left) and workflow of airway organoids with SARS-CoV-2 strains (right). In the second half of 2020, SARS-CoV-2 VOCs with a combination of several mutations emerged, including Alpha, first described in southeast England(Volz et al., 2021), and Beta, first identified in South Africa(Tegally et al., 2021). In February–March 2021, Alpha rapidly became the prevailing variant in many regions of the world and a higher reproduction number was inferred from early epidemiological data (Davies et al., 2021; Wagar et al., 2021; Washington et al., 2021). Beyond S(D614G), Alpha has 18 further mutations in its genome compared with the progenitor, with two deletions and six substitutions within the *S* gene The Delta SARS-CoV-2 variant, B.1.617.2 accumulated eight amino acid mutations in the S protein, including T19R, G142D, FR156-157del, R158G, L452R, T478K, P681R, D950N (Harvey et al., 2021).

F) Viral titers generated from Vero E6 cells infected with SARS-CoV-2-WA-1, -Delta and -Omicron Strains (5 days p.i., MOI=0.01).

G) Percentage of SARS-CoV-2 Nucleocapsid+ in live mCherry, TSPAN8 and CD9 expressing HEK293T cell at 48h post SARS-CoV-2 WA-1 or Delta infection. H)

Absolute number of live cells ACE2 mCherry-, ACE2 TSPAN8- and ACE2 CD9-expressing HEK293T cell at 24h post-SARS-CoV-2 WA-, -Delta, or -Omicron infection (MOI=0.3); at least 3 independent experiments with 5 replicates each were performed.

Supplementary Figure 7: Infection of airway organoids by SARS-CoV-2 VOCs

A) Pie charts representing distribution of cell populations in organoids Mock, infected with SARS-CoV-2-WA-1, Delta and Omicron at 72h p.i. (MOI=0.3). The pie chart fraction represents the mean of the cell populations for 3 independent experiments with at least 3 replicates.

B-F) Percentage of B) acTUBA+ cells, C) cKit+ cells, D) TSPAN8+ MUC5AC- , E) CD271+ or CD49f+ cells, F) CD86+ cells in in AO Mock or infected with SARS-CoV-2 -WA-1, -Delta or -Omicron at 72h p.i. (MOI=0.3), 3 independent experiments with 3 replicates for the distinct donor-derived organoids were performed; non parametric ANOVA tested corrected by Geisser Greenhouse Correction *p<0.05, **p<0.001, ***p<0.001.

G) Stack bar-charts representing of dsRNA+ cells (in red) in SARS-CoV-2 infected organoids (left) and the fraction of cell types infected by SARS-CoV-2 WA-1, -delta or -omicron (dsRNA+) at 72h p.i., MOI=0.3 Every fraction of the stack bar chart shows the mean of the distinctive cell population for every single independent experiment for the same donor, 3 independent experiments with 5 replicates each.; non parametric ANOVA tested corrected by Geisser Greenhouse Correction *p<0.05, **p<0.001, ***p<0.001.

H) Percentage of ACE2+ cells in 2522 and 2450 airway organoids Mock or infected with SARS-CoV-2 -WA-1, -Delta or -Omicron at 72h p.i. (MOI=0.3), , 3 independent experiments with 5 replicates each, nonparametric ANOVA tested corrected by Geisser Greenhouse Correction *p<0.05, **p<0.001, ***p<0.001.

I) As in S8H but for TSPAN8+ cells in 2522 and 2450 airway organoids.

J-K) Spectral flow cytometry layouts (J) and quantification (K) at 72h post SARS-CoV-2 -WA-1 infection of dsRNA-positive cells in organoids pre-treated with TSPAN8 and/or ACE2 blocking antibody (50µg/ml); representative data from 2 independent experiment with 4 replicates each.

References

Barberis, E., Vanella, V.V., Falasca, M., Caneparo, V., Cappellano, G., Raineri, D., Ghirimoldi, M., De Giorgis, V., Puricelli, C., and Vaschetto, R. (2021). Circulating exosomes are strongly involved in SARS-CoV-2 infection. Frontiers in molecular biosciences *8*, 29.

Bastard, P., Gervais, A., Le Voyer, T., Rosain, J., Philippot, Q., Manry, J., Michailidis, E., Hoffmann, H.H., Eto, S., Garcia-Prat, M.*, et al.* (2021). Autoantibodies neutralizing type I IFNs are present in ~4% of uninfected individuals over 70 years old and account for ~20% of COVID-19 deaths. Sci Immunol *6*.

Becht, E., McInnes, L., Healy, J., Dutertre, C.A., Kwok, I.W.H., Ng, L.G., Ginhoux, F., and Newell, E.W. (2018). Dimensionality reduction for visualizing single-cell data using UMAP. Nat Biotechnol.

Bonnet, M., Maisonial-Besset, A., Zhu, Y., Witkowski, T., Roche, G., Boucheix, C., Greco, C., and Degoul, F. (2019). Targeting the tetraspanins with monoclonal antibodies in oncology: focus on Tspan8/Co-029. Cancers *11*, 179.

Bonser, L.R., Koh, K.D., Johansson, K., Choksi, S.P., Cheng, D., Liu, L., Sun, D.I., Zlock, L.T., Eckalbar, W.L., and Finkbeiner, W.E. (2021). Flow-cytometric analysis and purification of airway epithelial-cell subsets. American Journal of Respiratory Cell and Molecular Biology *64*, 308-317.

Brouwer, P.J., Caniels, T.G., van der Straten, K., Snitselaar, J.L., Aldon, Y., Bangaru, S., Torres, J.L., Okba, N.M., Claireaux, M., and Kerster, G. (2020). Potent neutralizing antibodies from COVID-19 patients define multiple targets of vulnerability. Science *369*, 643-650.

Cantuti-Castelvetri, L., Ojha, R., Pedro, L.D., Djannatian, M., Franz, J., Kuivanen, S., van der Meer, F., Kallio, K., Kaya, T., and Anastasina, M. (2020). Neuropilin-1 facilitates SARS-CoV-2 cell entry and infectivity. Science *370*, 856-860.

Cele, S., Jackson, L., Khoury, D.S., Khan, K., Moyo-Gwete, T., Tegally, H., San, J.E., Cromer, D., Scheepers, C., and Amoako, D.G. (2022). Omicron extensively but incompletely escapes Pfizer BNT162b2 neutralization. Nature *602*, 654-656.

Chen, G.-Y., Tang, J., Zheng, P., and Liu, Y. (2009). CD24 and Siglec-10 selectively repress tissue damage–induced immune responses. Science *323*, 1722-1725.

Chi, X., Yan, R., Zhang, J., Zhang, G., Zhang, Y., Hao, M., Zhang, Z., Fan, P., Dong, Y., and Yang, Y. (2020). A neutralizing human antibody binds to the N-terminal domain of the Spike protein of SARS-CoV-2. Science *369*, 650-655.

Chua, R.L., Lukassen, S., Trump, S., Hennig, B.P., Wendisch, D., Pott, F., Debnath, O., Thurmann, L., Kurth, F., Volker, M.T.*, et al.* (2020). COVID-19 severity correlates with airway epithelium-immune cell interactions identified by single-cell analysis. Nat Biotechnol *38*, 970-979.

Co, J.Y., Margalef-Catala, M., Li, X., Mah, A.T., Kuo, C.J., Monack, D.M., and Amieva, M.R. (2019). Controlling Epithelial Polarity: A Human Enteroid Model for Host-Pathogen Interactions. Cell Rep *26*, 2509-2520 e2504.

Cobey, S., Larremore, D.B., Grad, Y.H., and Lipsitch, M. (2021). Concerns about SARS-CoV-2 evolution should not hold back efforts to expand vaccination. Nat Rev Immunol *21*, 330-335.

Combes, A.J., Courau, T., Kuhn, N.F., Hu, K.H., Ray, A., Chen, W.S., Clearly, S.J., Chew, N.W., Kushnoor, D., and Reeder, G.C. (2020). Global Absence and Targeting of Protective Immune States in Severe COVID-19. bioRxiv.

Corbiere, V., Dirix, V., Norrenberg, S., Cappello, M., Remmelink, M., and Mascart, F. (2011). Phenotypic characteristics of human type II alveolar epithelial cells suitable for antigen presentation to T lymphocytes. Respir Res *12*, 15.

Daly, J.L., Simonetti, B., Klein, K., Chen, K.-E., Williamson, M.K., Antón-Plágaro, C., Shoemark, D.K., Simón-Gracia, L., Bauer, M., and Hollandi, R. (2020). Neuropilin-1 is a host factor for SARS-CoV-2 infection. Science *370*, 861-865.

Davies, N.G., Abbott, S., Barnard, R.C., Jarvis, C.I., Kucharski, A.J., Munday, J.D., Pearson, C.A., Russell, T.W., Tully, D.C., and Washburne, A.D. (2021). Estimated transmissibility and impact of SARS-CoV-2 lineage B. 1.1. 7 in England. Science *372*, eabg3055.

Earnest, J.T., Hantak, M.P., Li, K., McCray Jr, P.B., Perlman, S., and Gallagher, T. (2017). The tetraspanin CD9 facilitates MERS-coronavirus entry by scaffolding host cell receptors and proteases. PLoS pathogens *13*, e1006546.

Earnest, J.T., Hantak, M.P., Park, J.E., and Gallagher, T. (2015). Coronavirus and influenza virus proteolytic priming takes place in tetraspanin-enriched membrane microdomains. J Virol *89*, 6093-6104.

Escalera, A., Gonzalez-Reiche, A.S., Aslam, S., Mena, I., Laporte, M., Pearl, R.L., Fossati, A., Rathnasinghe, R., Alshammary, H., van de Guchte, A.*, et al.* (2022). Mutations in SARS-CoV-2 variants of concern link to increased spike cleavage and virus transmission. Cell Host Microbe *30*, 373-387 e377.

Fang, S., Wei, J., Pentinmikko, N., Leinonen, H., and Salven, P. (2012). Generation of functional blood vessels from a single c-kit+ adult vascular endothelial stem cell. PLoS Biol *10*, e1001407.

Fiege, J.K., Thiede, J.M., Nanda, H.A., Matchett, W.E., Moore, P.J., Montanari, N.R., Thielen, B.K., Daniel, J., Stanley, E., Hunter, R.C.*, et al.* (2021). Single cell resolution of SARS-CoV-2 tropism, antiviral responses, and susceptibility to therapies in primary human airway epithelium. PLoS Pathog *17*, e1009292.

Finkelshtein, D., Werman, A., Novick, D., Barak, S., and Rubinstein, M. (2013). LDL receptor and its family members serve as the cellular receptors for vesicular stomatitis virus. Proc Natl Acad Sci U S A *110*, 7306-7311.

Firas A. Rabi 1, Mazhar S. Al Zoubi 2, , G.A.K., , D.M.S.a., and 4, A.D.A.-N. SARS-CoV-2 and Coronavirus Disease 2019: What We Know So Far. https://doiorg/103390/pathogens9030231.

Gao, Y., Cai, C., Grifoni, A., Muller, T.R., Niessl, J., Olofsson, A., Humbert, M., Hansson, L., Osterborg, A., Bergman, P.*, et al.* (2022). Ancestral SARS-CoV-2-specific T cells cross-recognize the Omicron variant. Nat Med *28*, 472-476.

Gonzalez, H., Mei, W., Robles, I., Hagerling, C., Allen, B.M., Okholm, T.L.H., Nanjaraj, A., Verbeek, T., Kalavacherla, S., and van Gogh, M. (2022). Cellular architecture of human brain metastases. Cell *185*, 729-745. e720.

Gordon, D.E., Jang, G.M., Bouhaddou, M., Xu, J., Obernier, K., White, K.M., O'Meara, M.J., Rezelj, V.V., Guo, J.Z., Swaney, D.L.*, et al.* (2020). A SARS-CoV-2 protein interaction map reveals targets for drug repurposing. Nature *583*, 459-468.

Gray, T., Coakley, R., Hirsh, A., Thornton, D., Kirkham, S., Koo, J.-S., Burch, L., Boucher, R., and Nettesheim, P. (2004). Regulation of MUC5AC mucin secretion and airway surface liquid metabolism by IL-1β in human bronchial epithelia. American Journal of Physiology-Lung Cellular and Molecular Physiology *286*, L320-L330.

Han, Y., Duan, X., Yang, L., Nilsson-Payant, B.E., Wang, P., Duan, F., Tang, X., Yaron, T.M., Zhang, T., Uhl, S.*, et al.* (2021). Identification of SARS-CoV-2 inhibitors using lung and colonic organoids. Nature *589*, 270-275.

Hantak, M.P., Qing, E., Earnest, J.T., and Gallagher, T. (2019). Tetraspanins: architects of viral entry and exit platforms. Journal of virology *93*.

Hao, Y., Hao, S., Andersen-Nissen, E., Mauck, W.M., Zheng, S., Butler, A., Lee, M.J., Wilk, A.J., Darby, C., and Zagar, M. (2020). Integrated analysis of multimodal single-cell data. bioRxiv.

Harvey, W.T., Carabelli, A.M., Jackson, B., Gupta, R.K., Thomson, E.C., Harrison, E.M., Ludden, C., Reeve, R., Rambaut, A., Consortium, C.-G.U., *et al.* (2021). SARS-CoV-2 variants, spike mutations and immune escape. Nat Rev Microbiol *19*, 409-424.

Heo, K., and Lee, S. (2020). TSPAN8 as a novel emerging therapeutic target in cancer for monoclonal antibody therapy. Biomolecules *10*, 388.

Hoffmann, M., Kleine-Weber, H., and Pöhlmann, S. (2020a). A multibasic cleavage site in the spike protein of SARS-CoV-2 is essential for infection of human lung cells. Molecular Cell.

Hoffmann, M., Kleine-Weber, H., Schroeder, S., Kruger, N., Herrler, T., Erichsen, S., Schiergens, T.S., Herrler, G., Wu, N.H., Nitsche, A., *et al.* (2020b). SARS-CoV-2 Cell Entry Depends on ACE2 and TMPRSS2 and Is Blocked by a Clinically Proven Protease Inhibitor. Cell *181*, 271-280 e278.

https://coronavirus.jhu.edu/map.html) (2022). COVID-19 Cases. John Hopkins University and Medicine.

Iketani, S., Liu, L., Guo, Y., Liu, L., Chan, J.F., Huang, Y., Wang, M., Luo, Y., Yu, J., Chu, H., *et al.* (2022). Antibody evasion properties of SARS-CoV-2 Omicron sublineages. Nature.

Kaneko, Y., Kuwano, K., Kunitake, R., Kawasaki, M., Hagimoto, N., and Hara, N. (2000). B7-1, B7-2 and class II MHC molecules in idiopathic pulmonary fibrosis and bronchiolitis obliterans-organizing pneumonia. European Respiratory Journal *15*, 49-55.

Kassambara, A., and Mundt, F. (2017). Package ‘factoextra’. Extract and visualize the results of multivariate data analyses *76*.

Kim, I.S., Jenni, S., Stanifer, M.L., Roth, E., Whelan, S.P., van Oijen, A.M., and Harrison, S.C. (2017). Mechanism of membrane fusion induced by vesicular stomatitis virus G protein. Proc Natl Acad Sci U S A *114*, E28-E36.

Kim, T.-K., Park, C.S., Jeoung, M.H., Lee, W.R., Go, N.K., Choi, J.R., Lee, T.S., Shim, H., and Lee, S. (2015). Generation of a human antibody that inhibits TSPAN8-mediated invasion of metastatic colorectal cancer cells. Biochemical and Biophysical Research Communications *468*, 774-780.

Kummer, D., Steinbacher, T., Schwietzer, M.F., Thölmann, S., and Ebnet, K. (2020). Tetraspanins: integrating cell surface receptors to functional microdomains in homeostasis and disease. Medical Microbiology and Immunology *209*, 397-405.

Lambrecht, B.N., and Hammad, H. (2010). The role of dendritic and epithelial cells as master regulators of allergic airway inflammation. The Lancet *376*, 835-843.
Lamers, M.M., Beumer, J., van der Vaart, J., Knoops, K., Puschhof, J., Breugem, T.I., Ravelli, R.B., van Schayck, J.P., Mykytyn, A.Z., and Duimel, H.Q. (2020). SARS-CoV-2 productively infects human gut enterocytes. Science.
Lê, S., Josse, J., and Mazet, F. (2008). Package ‘FactoMineR. J Stat Softw [Internet] *25*, 1-18.
Letuve, S., Sallon, C., Yang, X., Dumay, A., Bedja, S., Hamidi, F., Guillou, N., Mordant, P., Pretolani, M., and Taillé, C. (2019). Role of DNA methylation in Muc5AC hyperexpression in severe asthma (Eur Respiratory Soc).
Li, F., Li, W., Farzan, M., and Harrison, S.C. (2005). Structure of SARS coronavirus spike receptor-binding domain complexed with receptor. Science *309*, 1864-1868.
Lopez-Giraldo, A., Cruz, T., Molins, L., Guirao, A., Saco, A., Cuerpo, S., Ramirez, J., Agusti, A., and Faner, R. (2018). Characterization, localization and comparison of c-Kit+ lung cells in never smokers and smokers with and without COPD. BMC Pulm Med *18*, 123.
Martins, S.d.T., and Alves, L.R. (2020). Extracellular vesicles in viral infections: two sides of the same coin? Frontiers in Cellular and Infection Microbiology, 737.
Mason, R.J. (2020). Pathogenesis of COVID-19 from a cell biology perspective. Eur Respir J *55*.
Meng, B., Abdullahi, A., Ferreira, I., Goonawardane, N., Saito, A., Kimura, I., Yamasoba, D., Gerber, P.P., Fatihi, S., Rathore, S*., et al.* (2022). Altered TMPRSS2 usage by SARS-CoV-2 Omicron impacts infectivity and fusogenicity. Nature.
Merad, M., and Martin, J.C. (2020). Pathological inflammation in patients with COVID-19: a key role for monocytes and macrophages. Nat Rev Immunol *20*, 355-362.
Mick, E., Kamm, J., Pisco, A.O., Ratnasiri, K., Babik, J.M., Castañeda, G., DeRisi, J.L., Detweiler, A.M., Hao, S.L., and Kangelaris, K.N. (2020). Upper airway gene expression reveals suppressed immune responses to SARS-CoV-2 compared with other respiratory viruses. Nature communications *11*, 1-7.
Nawijn, M.C., and Timens, W. (2020). Can ACE 2 expression explain SARS-CoV-2 infection of the respiratory epithelia in COVID-19? Molecular Systems Biology *16*, e9841.
Nazarenko, I., Rana, S., Baumann, A., McAlear, J., Hellwig, A., Trendelenburg, M., Lochnit, G., Preissner, K.T., and Zöller, M. (2010). Cell surface tetraspanin Tspan8

contributes to molecular pathways of exosome-induced endothelial cell activation. Cancer research *70*, 1668-1678.

Pastorino, R., Pezzullo, A.M., Villani, L., Causio, F.A., Axfors, C., Contopoulos-Ioannidis, D.G., Boccia, S., and Ioannidis, J.P. (2022). Change in age distribution of COVID-19 deaths with the introduction of COVID-19 vaccination. Environmental research *204*, 112342.

Planas, D., Saunders, N., Maes, P., Guivel-Benhassine, F., Planchais, C., Buchrieser, J., Bolland, W.-H., Porrot, F., Staropoli, I., and Lemoine, F. (2022). Considerable escape of SARS-CoV-2 Omicron to antibody neutralization. Nature *602*, 671-675.

Planas, D., Veyer, D., Baidaliuk, A., Staropoli, I., Guivel-Benhassine, F., Rajah, M.M., Planchais, C., Porrot, F., Robillard, N., Puech, J.*, et al.* (2021). Reduced sensitivity of SARS-CoV-2 variant Delta to antibody neutralization. Nature *596*, 276-280.

Ravindra, N.G., Alfajaro, M.M., Gasque, V., Wei, J., Filler, R.B., Huston, N.C., Wan, H., Szigeti-Buck, K., Wang, B., Montgomery, R.R.*, et al.* (2020). Single-cell longitudinal analysis of SARS-CoV-2 infection in human bronchial epithelial cells. bioRxiv.

Robinot, R., Hubert, M., de Melo, G.D., Lazarini, F., Bruel, T., Smith, N., Levallois, S., Larrous, F., Fernandes, J., and Gellenoncourt, S. (2021). SARS-CoV-2 infection induces the dedifferentiation of multiciliated cells and impairs mucociliary clearance. Nature communications *12*, 1-16.

Ross, J.T., Nesseler, N., Lee, J.W., Ware, L.B., and Matthay, M.A. (2019). The ex vivo human lung: research value for translational science. JCI Insight *4*.

Sachs, N., Papaspyropoulos, A., Zomer-van Ommen, D.D., Heo, I., Bottinger, L., Klay, D., Weeber, F., Huelsz-Prince, G., Iakobachvili, N., Amatngalim, G.D.*, et al.* (2019). Long-term expanding human airway organoids for disease modeling. EMBO J *38*.

Salahudeen, A.A., Choi, S.S., Rustagi, A., Zhu, J., van Unen, V., de la O, S.M., Flynn, R.A., Margalef-Català, M., Santos, A.J., and Ju, J. (2020). Progenitor identification and SARS-CoV-2 infection in human distal lung organoids. Nature *588*, 670-675.

Sarma, A., Christenson, S.A., Byrne, A., Mick, E., Pisco, A.O., DeVoe, C., Deiss, T., Ghale, R., Zha, B.S., Tsitsiklis, A.*, et al.* (2021). Tracheal aspirate RNA sequencing identifies distinct immunological features of COVID-19 ARDS. Nat Commun *12*, 5152.

Schleimer, R.P., Kato, A., Kern, R., Kuperman, D., and Avila, P.C. (2007). Epithelium: at the interface of innate and adaptive immune responses. J Allergy Clin Immunol *120*, 1279-1284.

Schultze, J.L., and Aschenbrenner, A.C. (2021). COVID-19 and the human innate immune system. Cell *184*, 1671-1692.

Sette, A., and Crotty, S. (2021). Adaptive immunity to SARS-CoV-2 and COVID-19. Cell *184*, 861-880.

Shafiee, A., Moradi, L., Lim, M., and Brown, J. (2021). Coronavirus disease 2019: A tissue engineering and regenerative medicine perspective. Stem Cells Transl Med *10*, 27-38.

Shang, J., Wan, Y., Luo, C., Ye, G., Geng, Q., Auerbach, A., and Li, F. (2020). Cell entry mechanisms of SARS-CoV-2. Proc Natl Acad Sci U S A *117*, 11727-11734.

Shilts, J., Crozier, T.W., Greenwood, E.J., Lehner, P.J., and Wright, G.J. (2021). No evidence for basigin/CD147 as a direct SARS-CoV-2 spike binding receptor. Scientific reports *11*, 1-10.

Sigal, A. (2022). Milder disease with Omicron: is it the virus or the pre-existing immunity? Nature Reviews Immunology, 1-3.

Simon-Loriere, E., and Schwartz, O. (2022). Towards SARS-CoV-2 serotypes? Nat Rev Microbiol *20*, 187-188.

Simoneau, C.R., and Ott, M. (2020). Modeling Multi-organ Infection by SARS-CoV-2 Using Stem Cell Technology. Cell Stem Cell *27*, 859-868.

Skevaki, C., Karsonova, A., Karaulov, A., Fomina, D., Xie, M., Chinthrajah, S., Nadeau, K.C., and Renz, H. (2021). SARS-CoV-2 infection and COVID-19 in asthmatics: a complex relationship. Nature Reviews Immunology *21*, 202-203.

Smith, J.C., and Sheltzer, J.M. (2020).

Suryawanshi, R.K., Chen, I.P., Ma, T., Syed, A.M., Brazer, N., Saldhi, P., Simoneau, C.R., Ciling, A., Khalid, M.M., Sreekumar, B.*, et al.* (2022). Limited Cross-Variant Immunity after Infection with the SARS-CoV-2 Omicron Variant Without Vaccination. medRxiv.

Tarke, A., Coelho, C.H., Zhang, Z., Dan, J.M., Yu, E.D., Methot, N., Bloom, N.I., Goodwin, B., Phillips, E., Mallal, S.*, et al.* (2022). SARS-CoV-2 vaccination induces immunological T cell memory able to cross-recognize variants from Alpha to Omicron. Cell *185*, 847-859 e811.

Team, R.C. (2013). R: A language and environment for statistical computing.

Tegally, H., Wilkinson, E., Giovanetti, M., Iranzadeh, A., Fonseca, V., Giandhari, J., Doolabh, D., Pillay, S., San, E.J., and Msomi, N. (2021). Detection of a SARS-CoV-2 variant of concern in South Africa. Nature *592*, 438-443.

Thorne, L.G., Bouhaddou, M., Reuschl, A.-K., Zuliani-Alvarez, L., Polacco, B., Pelin, A., Batra, J., Whelan, M.V., Hosmillo, M., and Fossati, A. (2022). Evolution of enhanced innate immune evasion by SARS-CoV-2. Nature *602*, 487-495.

Travaglini, K.J., Nabhan, A.N., Penland, L., Sinha, R., Gillich, A., Sit, R.V., Chang, S., Conley, S.D., Mori, Y., and Seita, J. (2020). A molecular cell atlas of the human lung from single-cell RNA sequencing. Nature *587*, 619-625.

VanBlargan, L.A., Errico, J.M., Halfmann, P.J., Zost, S.J., Crowe, J.E., Purcell, L.A., Kawaoka, Y., Corti, D., Fremont, D.H., and Diamond, M.S. (2022). An infectious SARS-CoV-2 B. 1.1. 529 Omicron virus escapes neutralization by therapeutic monoclonal antibodies. Nature medicine, 1-6.

Vieira Braga, F.A., Kar, G., Berg, M., Carpaij, O.A., Polanski, K., Simon, L.M., Brouwer, S., Gomes, T., Hesse, L., and Jiang, J. (2019). A cellular census of human lungs identifies novel cell states in health and in asthma. Nature medicine *25*, 1153-1163.

Voglstaetter, M., Thomsen, A.R., Nouvel, J., Koch, A., Jank, P., Navarro, E.G., Gainey-Schleicher, T., Khanduri, R., Groß, A., and Rossner, F. (2019). Tspan8 is expressed in breast cancer and regulates E-cadherin/catenin signalling and metastasis accompanied by increased circulating extracellular vesicles. The Journal of pathology *248*, 421-437.

Volz, E., Mishra, S., Chand, M., Barrett, J.C., Johnson, R., Geidelberg, L., Hinsley, W.R., Laydon, D.J., Dabrera, G., and O'Toole, Á. (2021). Assessing transmissibility of SARS-CoV-2 lineage B. 1.1. 7 in England. Nature *593*, 266-269.

Wagar, L.E., Salahudeen, A., Constantz, C.M., Wendel, B.S., Lyons, M.M., Mallajosyula, V., Jatt, L.P., Adamska, J.Z., Blum, L.K., Gupta, N., *et al.* (2021). Modeling human adaptive immune responses with tonsil organoids. Nat Med *27*, 125-135.

Walls, A.C., Park, Y.J., Tortorici, M.A., Wall, A., McGuire, A.T., and Veesler, D. (2020). Structure, Function, and Antigenicity of the SARS-CoV-2 Spike Glycoprotein. Cell *181*, 281-292 e286.

Walls, A.C., Tortorici, M.A., Bosch, B.-J., Frenz, B., Rottier, P.J., DiMaio, F., Rey, F.A., and Veesler, D. (2016). Cryo-electron microscopy structure of a coronavirus spike glycoprotein trimer. Nature *531*, 114-117.

Wang, K., Chen, W., Zhou, Y.-S., Lian, J.-Q., Zhang, Z., Du, P., Gong, L., Zhang, Y., Cui, H.-Y., and Geng, J.-J. (2020). SARS-CoV-2 invades host cells via a novel route: CD147-spike protein. BioRxiv.
Wang, R., Simoneau, C.R., Kulsuptrakul, J., Bouhaddou, M., Travisano, K.A., Hayashi, J.M., Carlson-Stevermer, J., Zengel, J.R., Richards, C.M., and Fozouni, P. (2021a). Genetic screens identify host factors for SARS-CoV-2 and common cold coronaviruses. Cell *184*, 106-119. e114.
Wang, S., Qiu, Z., Hou, Y., Deng, X., Xu, W., Zheng, T., Wu, P., Xie, S., Bian, W., Zhang, C.*, et al.* (2021b). AXL is a candidate receptor for SARS-CoV-2 that promotes infection of pulmonary and bronchial epithelial cells. Cell Res.
Washington, N.L., Gangavarapu, K., Zeller, M., Bolze, A., Cirulli, E.T., Barrett, K.M.S., Larsen, B.B., Anderson, C., White, S., and Cassens, T. (2021). Emergence and rapid transmission of SARS-CoV-2 B. 1.1. 7 in the United States. Cell *184*, 2587-2594. e2587.
Wickham, H., Chang, W., Henry, L., Pedersen, T., Takahashi, K., Wilke, C., Woo, K., Yutani, H., and Dunnington, D. (2016). Springer-Verlag. New York.
Wickham, H., Francois, R., Henry, L., and Müller, K. others. 2015.". Dplyr: A Grammar of Data Manipulation" R Package Version 04 *3*.
Xian, W., and McKeon, F. (2012). Adult stem cells underlying lung regeneration. Cell Cycle *11*, 887-894.
Yan, R., Zhang, Y., Li, Y., Xia, L., Guo, Y., and Zhou, Q. (2020). Structural basis for the recognition of SARS-CoV-2 by full-length human ACE2. Science *367*, 1444-1448.
Youk, J., Kim, T., Evans, K.V., Jeong, Y.I., Hur, Y., Hong, S.P., Kim, J.H., Yi, K., Kim, S.Y., Na, K.J.*, et al.* (2020). Three-Dimensional Human Alveolar Stem Cell Culture Models Reveal Infection Response to SARS-CoV-2. Cell Stem Cell *27*, 905-919 e910.
Zhou, Y., Zhou, B., Pache, L., Chang, M., Khodabakhshi, A.H., Tanaseichuk, O., Benner, C., and Chanda, S.K. (2019). Metascape provides a biologist-oriented resource for the analysis of systems-level datasets. Nature communications *10*, 1-10.
Ziegler, C.G.K., Allon, S.J., Nyquist, S.K., Mbano, I.M., Miao, V.N., Tzouanas, C.N., Cao, Y., Yousif, A.S., Bals, J., Hauser, B.M.*, et al.* (2020). SARS-CoV-2 Receptor ACE2 Is an Interferon-Stimulated Gene in Human Airway Epithelial Cells and Is Detected in Specific Cell Subsets across Tissues. Cell *181*, 1016-1035 e1019.
Zissel, G., Ernst, M., Rabe, K., Papadopoulos, T., Magnussen, H., Schlaak, M., and Müller-Quernheim, J. (2000). Human alveolar epithelial cells type II are capable of

regulating T-cell activity. Journal of investigative medicine: the official publication of the American Federation for Clinical Research *48*, 66-75.

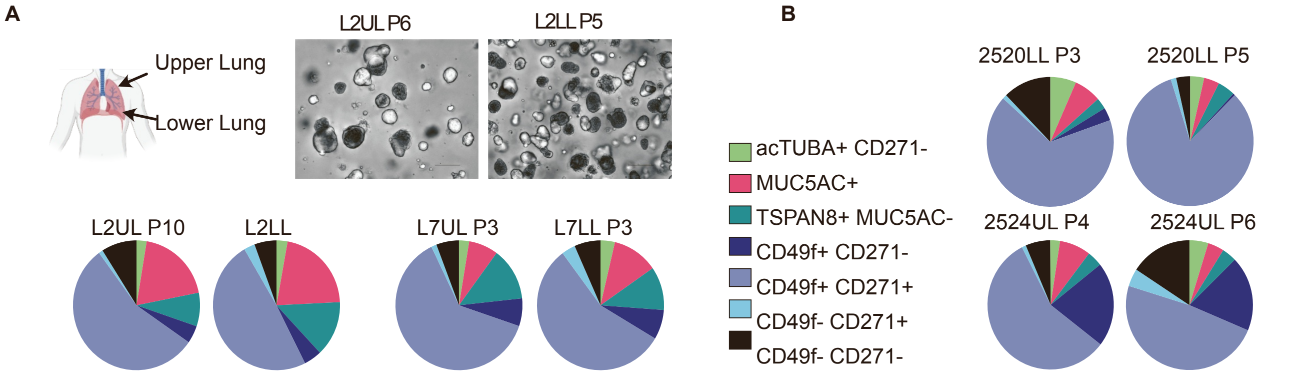

C

Sample ID	Tissue Type	P 1-5	P 6-10	P 11-16	P 17-20
2425	Normal Lung	11	15		
2477	Normal Lung	28	11		5
2478	Normal Lung	14	10	3	
2520	Normal Lung	14			
2521	Normal Lung	20	11	4	5
2522	Normal Lung	15	6	2	2
2523	Normal Lung	12	6	8	
2524	Normal Lung	16	9	9	
2525	Normal Lung	21		8	
2526	Normal Lung	16	2		
2527	Normal Lung	27			
2531	Normal Lung	4	21		
2547	Normal Lung	33			
2551	Normal Lung	16			
2553	Normal Lung	22			
2559	Normal Lung	11			
2561	Normal Lung	16	4		
L3	Normal Lung	5			
L5	Normal Lung	12			
L7	Normal Lung	5			

* Number of organoid cryovials in Organoid D2B Biobank (P = Passage).

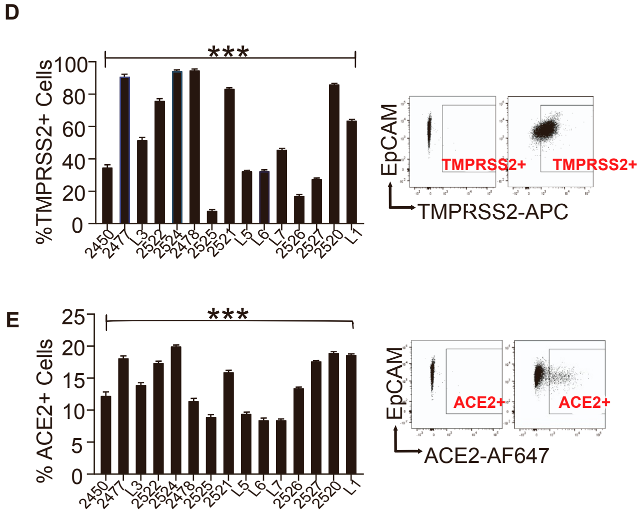

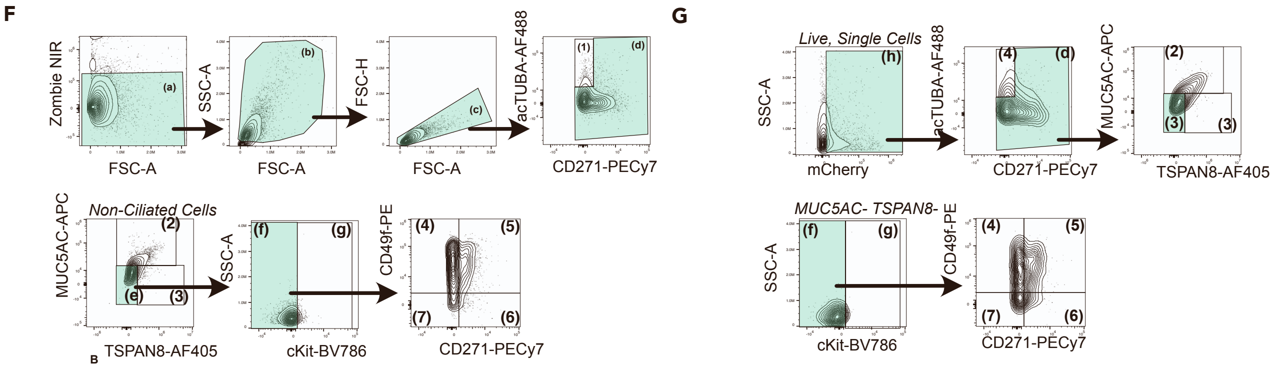

(a) Live Cells
(b) Cells
(c) Single Cells
(1) Ciliated Cells
(d) Non-Ciliated Cells
(2) Goblet-like cells
(e) MUC5AC- TSPAN8-
(3) Pre-Goblet-like cells

(f) cKit-
(g) cKit+
(4) CD49f+ CD271-
(5) CD49f+ CD271+
(6) CD49f- CD271-
(7) CD49f- CD271+
(h) mCherry+ Cells

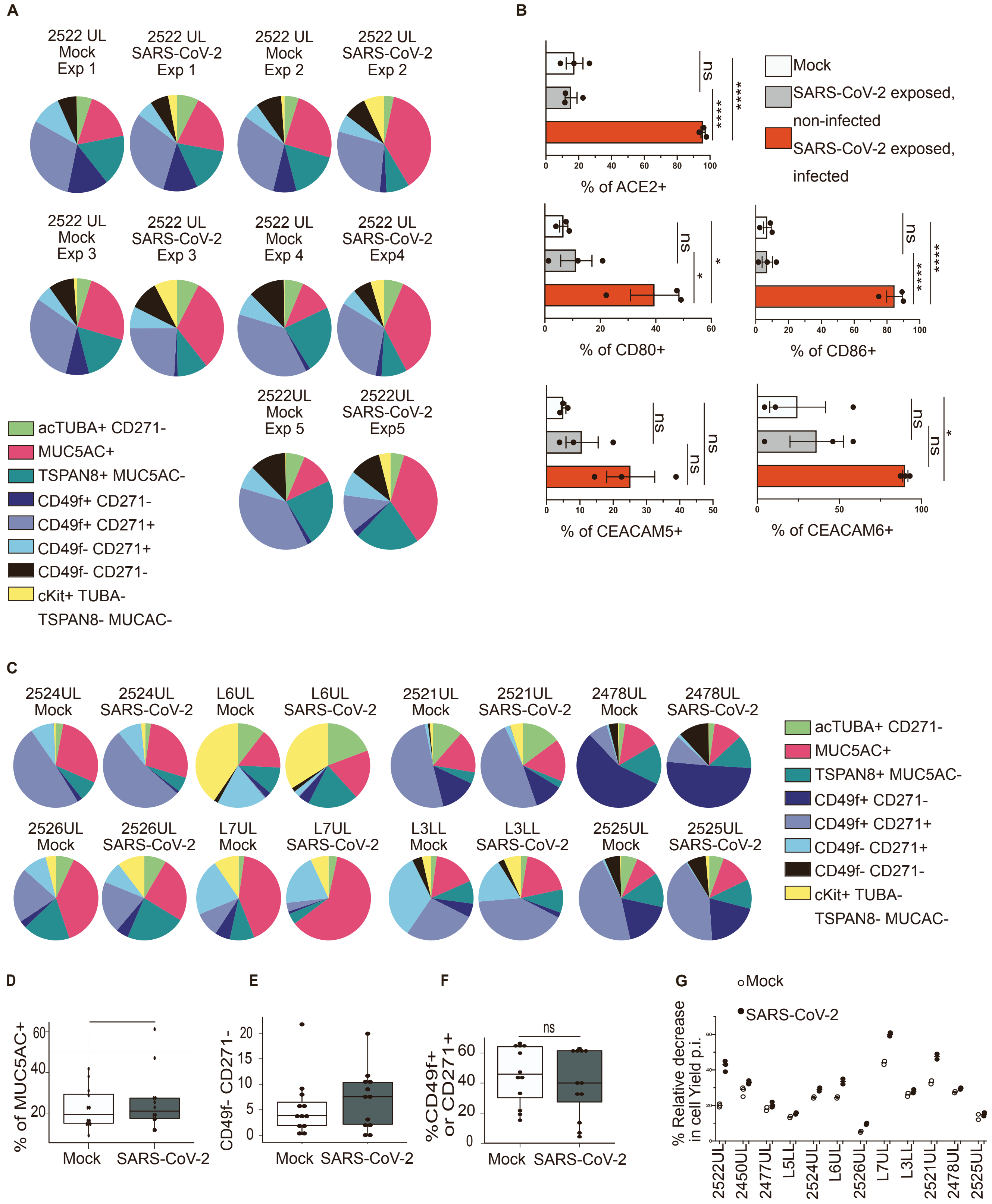
A
2522 UL Mock Exp 1
2522 UL SARS-CoV-2 Exp 1
2522 UL Mock Exp 2
2522 UL SARS-CoV-2 Exp 2
2522 UL Mock Exp 3
2522 UL SARS-CoV-2 Exp 3
2522 UL Mock Exp 4
2522 UL SARS-CoV-2 Exp4
2522UL Mock Exp 5
2522UL SARS-CoV-2 Exp5
acTUBA+ CD271-
MUC5AC+
TSPAN8+ MUC5AC-
CD49f+ CD271-
CD49f+ CD271+
CD49f- CD271+
CD49f- CD271-
cKit+ TUBA- TSPAN8- MUCAC-
B
Mock
SARS-CoV-2 exposed, non-infected
SARS-CoV-2 exposed, infected
% of ACE2+
% of CD80+
% of CD86+
% of CEACAM5+
% of CEACAM6+
ns
****
*
C
2524UL Mock
2524UL SARS-CoV-2
L6UL Mock
L6UL SARS-CoV-2
2521UL Mock
2521UL SARS-CoV-2
2478UL Mock
2478UL SARS-CoV-2
2526UL Mock
2526UL SARS-CoV-2
L7UL Mock
L7UL SARS-CoV-2
L3LL Mock
L3LL SARS-CoV-2
2525UL Mock
2525UL SARS-CoV-2
D
% of MUC5AC+
Mock
SARS-CoV-2
E
CD49f- CD271-
F
% CD49f+ or CD271+
G
Mock
SARS-CoV-2
% Relative decrease in cell Yield p.i.
2522UL
2450UL
2477UL
L5LL
2524UL
L6UL
2526UL
L7UL
L3LL
2521UL
2478UL
2525UL

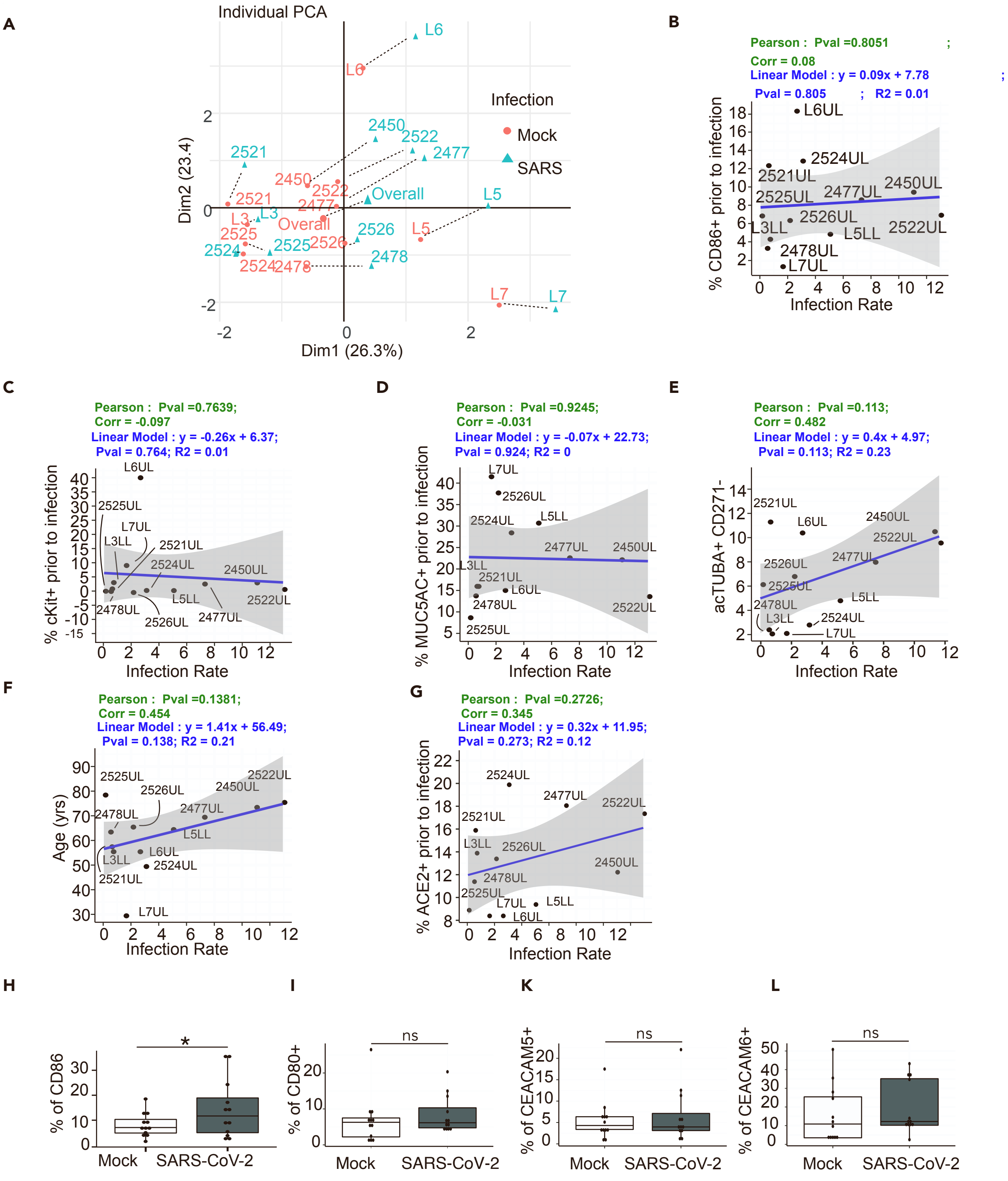
A
Individual PCA
Dim1 (26.3%)
Dim2 (23.4)
Infection
Mock
SARS
L6
2450
2522
2477
2521
Overall
L3
2525
2524
2526
2478
L5
L7
B
Pearson : Pval =0.8051 ;
Corr = 0.08
Linear Model : y = 0.09x + 7.78 ;
Pval = 0.805 ; R2 = 0.01
% CD86+ prior to infection
Infection Rate
L6UL
2524UL
2521UL
2525UL
2477UL
2450UL
2526UL
L3LL
L5LL
2522UL
2478UL
L7UL
C
Pearson : Pval =0.7639;
Corr = -0.097
Linear Model : y = -0.26x + 6.37;
Pval = 0.764; R2 = 0.01
% cKit+ prior to infection
Infection Rate
D
Pearson : Pval =0.9245;
Corr = -0.031
Linear Model : y = -0.07x + 22.73;
Pval = 0.924; R2 = 0
% MUC5AC+ prior to infection
Infection Rate
E
Pearson : Pval =0.113;
Corr = 0.482
Linear Model : y = 0.4x + 4.97;
Pval = 0.113; R2 = 0.23
acTUBA+ CD271-
Infection Rate
F
Pearson : Pval =0.1381;
Corr = 0.454
Linear Model : y = 1.41x + 56.49;
Pval = 0.138; R2 = 0.21
Age (yrs)
Infection Rate
G
Pearson : Pval =0.2726;
Corr = 0.345
Linear Model : y = 0.32x + 11.95;
Pval = 0.273; R2 = 0.12
% ACE2+ prior to infection
Infection Rate
H
% of CD86
*
Mock
SARS-CoV-2
I
% of CD80+
ns
Mock
SARS-CoV-2
K
% of CEACAM5+
ns
Mock
SARS-CoV-2
L
% of CEACAM6+
ns
Mock
SARS-CoV-2

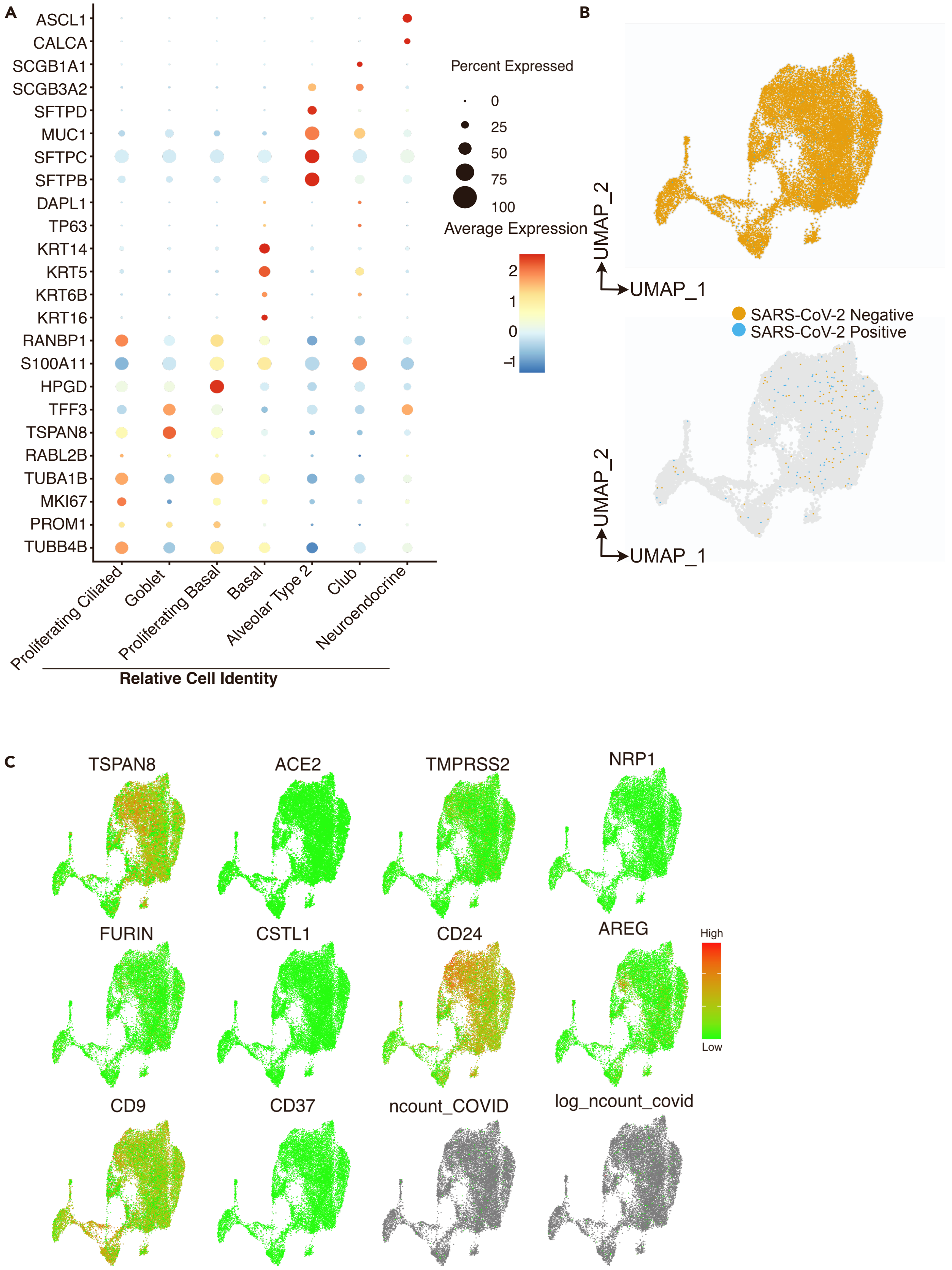
A
ASCL1
CALCA
SCGB1A1
SCGB3A2
SFTPD
MUC1
SFTPC
SFTPB
DAPL1
TP63
KRT14
KRT5
KRT6B
KRT16
RANBP1
S100A11
HPGD
TFF3
TSPAN8
RABL2B
TUBA1B
MKI67
PROM1
TUBB4B
Proliferating Ciliated
Goblet
Proliferating Basal
Basal
Alveolar Type 2
Club
Neuroendocrine
Relative Cell Identity
Percent Expressed
0
25
50
75
100
Average Expression
2
1
0
-1
B
UMAP_2
UMAP_1
SARS-CoV-2 Negative
SARS-CoV-2 Positive
UMAP_2
UMAP_1
C
TSPAN8
ACE2
TMPRSS2
NRP1
FURIN
CSTL1
CD24
AREG
High
Low
CD9
CD37
ncount_COVID
log_ncount_covid

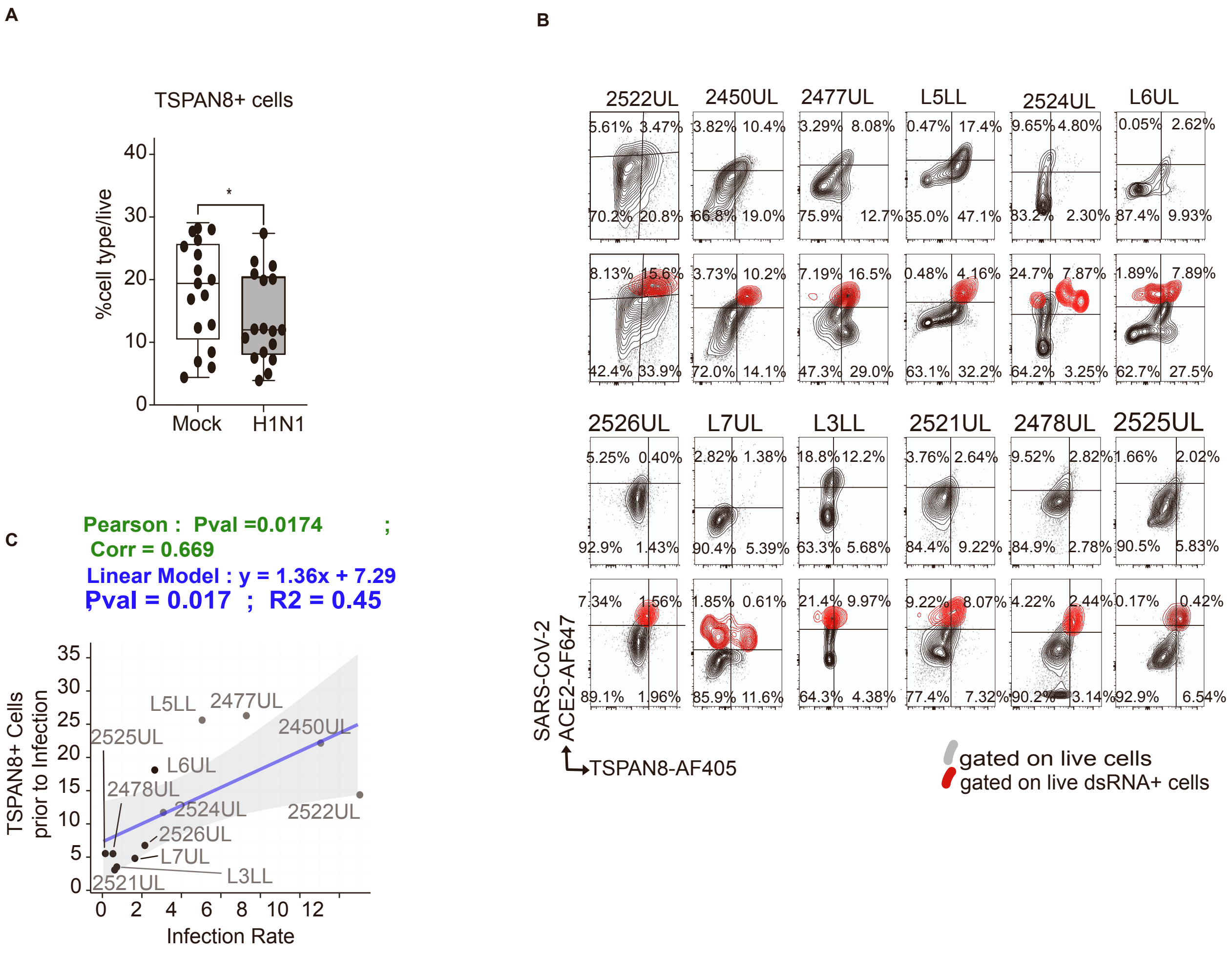
A
TSPAN8+ cells
%cell type/live
40
30
20
10
0
*
Mock
H1N1
B
2522UL
2450UL
2477UL
L5LL
2524UL
L6UL
2526UL
L7UL
L3LL
2521UL
2478UL
2525UL
SARS-CoV-2
ACE2-AF647
TSPAN8-AF405
gated on live cells
gated on live dsRNA+ cells
C
Pearson : Pval =0.0174 ;
Corr = 0.669
Linear Model : y = 1.36x + 7.29
Pval = 0.017 ; R2 = 0.45
TSPAN8+ Cells
prior to Infection
Infection Rate
35
30
25
20
15
10
5
0
0
2
4
6
8
10
12

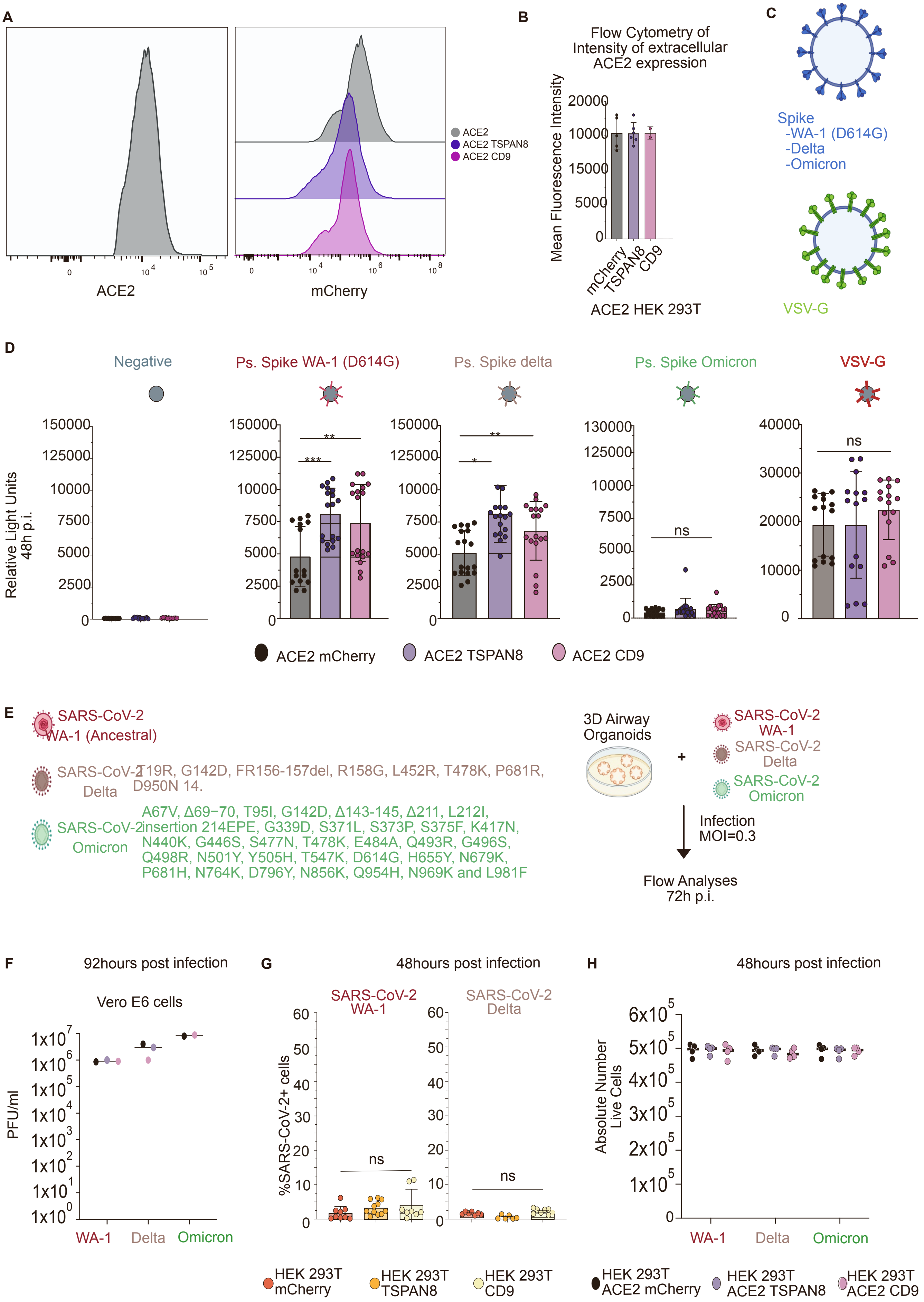
A
ACE2
mCherry
ACE2
ACE2 TSPAN8
ACE2 CD9
B
Flow Cytometry of Intensity of extracellular ACE2 expression
Mean Fluorescence Intensity
20000
10000
15000
5000
0
mCherry
TSPAN8
CD9
ACE2 HEK 293T
C
Spike
-WA-1 (D614G)
-Delta
-Omicron
VSV-G
D
Negative
Ps. Spike WA-1 (D614G)
Ps. Spike delta
Ps. Spike Omicron
VSV-G
Relative Light Units 48h p.i.
ns
ACE2 mCherry
ACE2 TSPAN8
ACE2 CD9
E
SARS-CoV-2 WA-1 (Ancestral)
SARS-CoV-2 Delta
T19R, G142D, FR156-157del, R158G, L452R, T478K, P681R, D950N 14.
SARS-CoV-2 Omicron
A67V, Δ69−70, T95I, G142D, Δ143-145, Δ211, L212I, insertion 214EPE, G339D, S371L, S373P, S375F, K417N, N440K, G446S, S477N, T478K, E484A, Q493R, G496S, Q498R, N501Y, Y505H, T547K, D614G, H655Y, N679K, P681H, N764K, D796Y, N856K, Q954H, N969K and L981F
3D Airway Organoids
+
SARS-CoV-2 WA-1
SARS-CoV-2 Delta
SARS-CoV-2 Omicron
Infection MOI=0.3
Flow Analyses 72h p.i.
F
92hours post infection
Vero E6 cells
PFU/ml
WA-1
Delta
Omicron
G
48hours post infection
SARS-CoV-2 WA-1
SARS-CoV-2 Delta
%SARS-CoV-2+ cells
ns
HEK 293T mCherry
HEK 293T TSPAN8
HEK 293T CD9
H
48hours post infection
Absolute Number Live Cells
WA-1
Delta
Omicron
HEK 293T ACE2 mCherry
HEK 293T ACE2 TSPAN8
HEK 293T ACE2 CD9

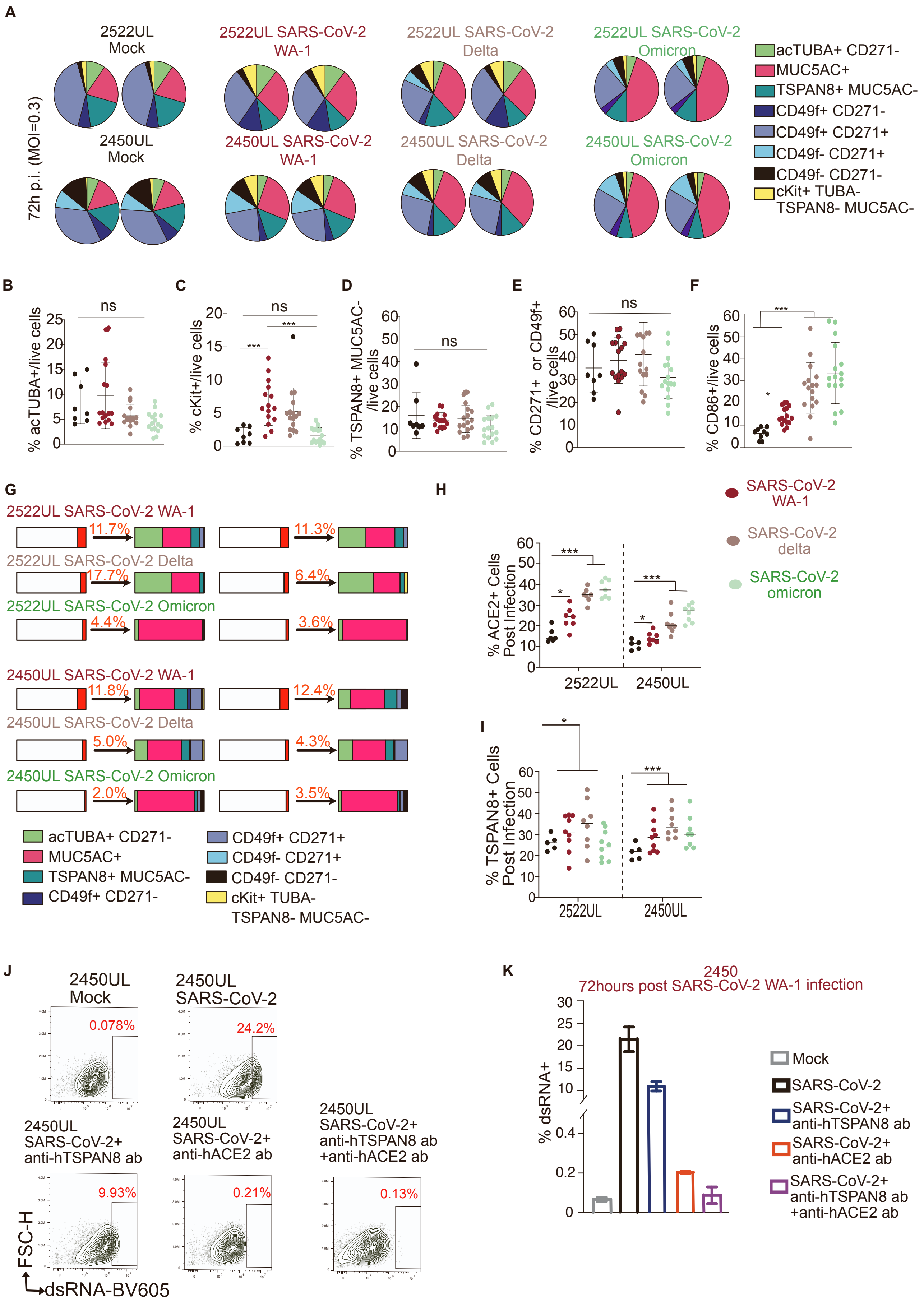

A
2522UL Mock
2522UL SARS-CoV-2 WA-1
2522UL SARS-CoV-2 Delta
2522UL SARS-CoV-2 Omicron
2450UL Mock
2450UL SARS-CoV-2 WA-1
2450UL SARS-CoV-2 Delta
2450UL SARS-CoV-2 Omicron
72h p.i. (MOI=0.3)
acTUBA+ CD271-
MUC5AC+
TSPAN8+ MUC5AC-
CD49f+ CD271-
CD49f+ CD271+
CD49f- CD271+
CD49f- CD271-
cKit+ TUBA- TSPAN8- MUC5AC-
B
% acTUBA+/live cells
C
% cKit+/live cells
D
% TSPAN8+ MUC5AC- /live cells
E
% CD271+ or CD49f+ /live cells
F
% CD86+/live cells
ns
***
*
G
2522UL SARS-CoV-2 WA-1
11.7%
11.3%
2522UL SARS-CoV-2 Delta
17.7%
6.4%
2522UL SARS-CoV-2 Omicron
4.4%
3.6%
2450UL SARS-CoV-2 WA-1
11.8%
12.4%
2450UL SARS-CoV-2 Delta
5.0%
4.3%
2450UL SARS-CoV-2 Omicron
2.0%
3.5%
H
% ACE2+ Cells Post Infection
2522UL
2450UL
SARS-CoV-2 WA-1
SARS-CoV-2 delta
SARS-CoV-2 omicron
I
% TSPAN8+ Cells Post Infection
2522UL
2450UL
J
2450UL Mock
0.078%
2450UL SARS-CoV-2
24.2%
2450UL SARS-CoV-2+ anti-hTSPAN8 ab
9.93%
2450UL SARS-CoV-2+ anti-hACE2 ab
0.21%
2450UL SARS-CoV-2+ anti-hTSPAN8 ab +anti-hACE2 ab
0.13%
FSC-H
dsRNA-BV605
K
2450
72hours post SARS-CoV-2 WA-1 infection
% dsRNA+
Mock
SARS-CoV-2
SARS-CoV-2+ anti-hTSPAN8 ab
SARS-CoV-2+ anti-hACE2 ab
SARS-CoV-2+ anti-hTSPAN8 ab +anti-hACE2 ab
